# Homologous Transcription Factors DUX4 and DUX4c Associate with Cytoplasmic Proteins during Muscle Differentiation

**DOI:** 10.1371/journal.pone.0146893

**Published:** 2016-01-27

**Authors:** Eugénie Ansseau, Jocelyn O. Eidahl, Céline Lancelot, Alexandra Tassin, Christel Matteotti, Cassandre Yip, Jian Liu, Baptiste Leroy, Céline Hubeau, Cécile Gerbaux, Samuel Cloet, Armelle Wauters, Sabrina Zorbo, Pierre Meyer, Isabelle Pirson, Dalila Laoudj-Chenivesse, Ruddy Wattiez, Scott Q. Harper, Alexandra Belayew, Frédérique Coppée

**Affiliations:** 1 Laboratory of Molecular Biology, Research Institute for Health Sciences and Technology, University of Mons, Mons, Belgium; 2 Center for Gene Therapy, Research Institute at Nationwide Children's Hospital, Columbus, OH, United States of America; 3 Laboratory of Proteomic and Microbiology, Research Institute for Biosciences, University of Mons, Mons, Belgium; 4 Pediatric Department, CHRU Montpellier, Montpellier, France; 5 I.R.I.B.H.M., Free University of Brussels, Brussels, Belgium; 6 Laboratory of Physiology and Experimental Medicine, INSERM U1046, Montpellier, France; 7 Department of Pediatrics, Ohio State University College of Medicine, Columbus, OH, United States of America; University of Minnesota, UNITED STATES

## Abstract

Hundreds of double homeobox (*DUX)* genes map within 3.3-kb repeated elements dispersed in the human genome and encode DNA-binding proteins. Among these, we identified DUX4, a potent transcription factor that causes facioscapulohumeral muscular dystrophy (FSHD). In the present study, we performed yeast two-hybrid screens and protein co-purifications with HaloTag-DUX fusions or GST-DUX4 pull-down to identify protein partners of DUX4, DUX4c (which is identical to DUX4 except for the end of the carboxyl terminal domain) and DUX1 (which is limited to the double homeodomain). Unexpectedly, we identified and validated (by co-immunoprecipitation, GST pull-down, co-immunofluorescence and *in situ* Proximal Ligation Assay) the interaction of DUX4, DUX4c and DUX1 with type III intermediate filament protein desmin in the cytoplasm and at the nuclear periphery. Desmin filaments link adjacent sarcomere at the Z-discs, connect them to sarcolemma proteins and interact with mitochondria. These intermediate filament also contact the nuclear lamina and contribute to positioning of the nuclei. Another Z-disc protein, LMCD1 that contains a LIM domain was also validated as a DUX4 partner. The functionality of DUX4 or DUX4c interactions with cytoplasmic proteins is underscored by the cytoplasmic detection of DUX4/DUX4c upon myoblast fusion. In addition, we identified and validated (by co-immunoprecipitation, co-immunofluorescence and *in situ* Proximal Ligation Assay) as DUX4/4c partners several RNA-binding proteins such as C1QBP, SRSF9, RBM3, FUS/TLS and SFPQ that are involved in mRNA splicing and translation. FUS and SFPQ are nuclear proteins, however their cytoplasmic translocation was reported in neuronal cells where they associated with ribonucleoparticles (RNPs). Several other validated or identified DUX4/DUX4c partners are also contained in mRNP granules, and the co-localizations with cytoplasmic DAPI-positive spots is in keeping with such an association. Large muscle RNPs were recently shown to exit the nucleus via a novel mechanism of nuclear envelope budding. Following DUX4 or DUX4c overexpression in muscle cell cultures, we observed their association with similar nuclear buds. In conclusion, our study demonstrated unexpected interactions of DUX4/4c with cytoplasmic proteins playing major roles during muscle differentiation. Further investigations are on-going to evaluate whether these interactions play roles during muscle regeneration as previously suggested for DUX4c.

## Introduction

Repeated DNA elements constitute a large portion of the human genome and were long considered to be “junk DNA”. However, recent high-throughput sequence analyses have shown that RNAs expressed from these repeated regions had been excluded by the previous tools for transcriptomic study [1]. The Double Homeobox genes map to 3.3-kb repeated elements and constitute a family containing hundreds of members dispersed throughout the human genome; they are located on the short arms of all the acrocentric chromosomes, on the centromeric region of chromosome 1 and in the telomeric regions of chromosomes 4 and 10 [2–5]. The *DUX* genes have a highly conserved ORF encompassing one or two homeoboxes (reviewed in [6]). The most studied gene in this family is *DUX4*, which maps to a 3.3-kb element repeated at the *D4Z4* locus in 4q35 [4, 7, 8]. This locus is genetically linked to facioscapulohumeral muscular dystrophy (FSHD), and after over a decade of controversy, activation of the *DUX4* gene is now generally recognized as required to develop FSHD [9–12], reviewed in [13, 14]. In addition, the evolutionary conservation of the *DUX* gene indicates that it has a key functional role [15, 16].

Because the *DUX* genes lie within repeated elements, they were mostly excluded from the Human Genome Project. However, *in silico* analyses identified different loci in the human genome containing *DUX* sequences [17, 18]. The evolution of this gene family is complex because the homeobox sequence (or sequences) of an ancient *Dux* gene has become incorporated into repetitive DNA elements found in both heterochromatin and euchromatin regions. Most of the *DUX4-*like sequences lack introns and are arranged in polymorphic arrays. Other *Dux* genes (*Duxc* and *Duxbl*) are present in some mammals but not in humans [15, 18].

In a previous study, our group first identified the *DUX1* gene, and a related cDNA was detected in the human rhabdomyosarcoma TE671 cell line. As expected based on its homeodomains, the encoded DUX1 protein can bind to a specific DNA sequence and activate the transcription of a linked reporter gene in transient co-expression experiments [4]. Using sequence alignments, we subsequently identified a homologous *DUX4* gene within each repeat unit of the *D4Z4* array in 4q35 and a second one, *DUX4c*, in a single truncated repeat unit located 42 kb from this locus toward the centromere [8, 19]. The *DUX4c* gene, which has the greatest sequence similarity to *DUX4*, was found to be upregulated in FSHD, and its overexpression induced proliferation and impaired differentiation in human muscle cells *in vitro*. Moreover, DUX4c is induced in Duchenne Muscular Dystrophy (DMD) muscle biopsies, suggesting a role in muscle regeneration [19].

Mouse Duxbl is critical for double negative thymocyte development and regulates myogenesis and reproductive development [20–22]. The human ortholog DUXO is a regulator of the gastrula organizer in human embryonic stem cells [23] and points to a role for *DUX4* and repetitive elements in mammalian germline evolution [24]. The expression of DUX4 induces neurogenesis during differentiation of murine embryonic stem cells [25]. DUX4 overexpression is toxic in a majority of proliferating cells and in differentiating myotubes presenting densely packed nuclei [9, 26], however DUX4 expression appears much less toxic in terminally differentiated myotubes [27]. To date, the functional studies of human DUX proteins have focused on DUX1/4 nuclear mobility [28], DUX4 nuclear localization [29], DUX1 and DUX4 DNA-binding sites and DUX4 transcriptional target genes in mouse and human cells [4, 10, 27, 30, 31]. DUX4 was recently reported to strongly inhibit nonsense-mediated mRNA decay (NMD) [32]. Finally, DUX4 overexpression has been found to inhibit protein turnover and cause the aggregation of TDP-43 [33]. To address the functions of DUX proteins in skeletal muscle, we aimed to identify the protein partners of DUX1, DUX4 and DUX4c. Using 4 complementary methods (two yeast two-hybrid assays and GST pull-down and co-purification in mammalian cells), we unexpectedly identified cytoplasmic protein partners. In addition, we detected DUX4 and DUX4c in the cytoplasm of muscle cells following their differentiation and validated several protein partners playing major role during muscle differentiation.

## Materials and Methods

### Ethics Statement

Primary human myoblasts were derived from muscle biopsies performed according to the current ethical and legislative rules of France, and written informed consent was obtained from all subjects, as directed by the ethical committee of CHU de Villeneuve (Montpellier, France) [34] or as described previously [35–37]. In addition, the uses of primary human myoblasts and muscle biopsies have been approved by the ethics committee of the University of Mons (ref # A901) and by the ethics committee of ULB-Erasme (Brussels ref #B2011/003).

### Plasmid constructions

The cDNAs encoding individual DUX proteins or domains (homeodomains or the carboxyl-terminal “tail”) to be used as “baits” in the yeast two-hybrid system were amplified by PCR (see S1 Table for primers with appropriate restriction sites) from *pCIneo-DUX1* ([4], *pCIneo-DUX4* or *pGEMdKpn42* (*DUX4*, [8, 9]) or *pCIneo-DUX4c* [19] and inserted in *pCR4-TOPO* (Invitrogen, Carlsbad, CA). The individual bait-encoding fragments were then excised by restriction and cloned in frame with the Gal4 DNA-binding domain in the *pGBT9* (*Trp1*^*+*^) or pGBKT7 (*Trp1*^*+*^) expression vector.

The *DUX4* ORF was subcloned from *pCR4-DUX4* into *pENTR1A* (Invitrogen) using *Sal*I-*Not*I endonucleases and transferred into *pDEST15* by homologous recombination (Gateway, Invitrogen) to express an amino-terminal protein fused to GST. *The DUX4c* ORF was amplified by PCR from *pENTR1A-DUX4c* [19] and cloned into *pENTR/D/TOPO* (Invitrogen) before being transferred into *pDEST15*.

We received the human *desmin* and *karyopherin 13*/*IPO13* complete cDNAs from Dr. D. Paulin (Institute of Myology, Paris) and Dr. J. E. Ploski (The Mount Sinai School of Medicine, New York), respectively. The *desmin* ORF was subcloned into *pDEST15* and *pGAD424* (Clontech, Mountain View, CA) to express a GST-desmin or a GAL4 AD-desmin fusion protein, respectively. The IPO13 ORF was sub-cloned into *pGEM7Z* (Promega, Madison, WI) downstream from the *SP6* promoter for transcription *in vitro*.

The full eGFP (GenBank Accession #U57608), DUX4c (GenBank Accession #AY500824) and DUX4 (GenBank Accession #AF117653) ORFs and the last 228 nucleotides of the DUX4 ORF were inserted into the pFN21A or the pFC14K vectors according to the manufacturer’s instructions (Promega) to express amino- or carboxyl-terminal HaloTag fusion proteins, respectively, and referred to as HaloTag-eGFP, HaloTag-DUX4c and HaloTag-DUX4 and HaloTag-DUX4term. All fusion proteins contain a TEV cleavage site at the HaloTag carboxyl or amino terminus, respectively.

C-terminal V5-epitope-tagged DUX4 was cloned into a mammalian expression vector, as previously described (ref PMCID: PMC4098764).

The constructs were verified by DNA sequencing (Beckman Coulter, Fullerton, CA).

### Yeast two-hybrid screen

The yeast host strain used for the screening and the reconstruction steps was the pJ69-4 A strain (MAT a, ade 2 trp 1-Δ901 leu 2–3, 112 ura 3–52 his 3–200 Gal4Δ Gal80Δ LYS2::Gal1-HIS3 ADE2::Gal2-ADE2) or AH109 (a derivative of pJ69 4A with the addition of a lacZ reporter gene under the control of MEL1, an endogenous GAL4 responsive element). For the screen, pJ69-4 A or AH109 cells harboring *pGBT9-DUX1*, *pGBT9-DUX4c* or *pGBKT7*-*DUX4* were co-transformed with an adult human skeletal muscle or a mouse embryonic day 8 Matchmaker library in *pACT2* (“prey” cDNAs fused to *GAL4 AD*, *Leu2*^+^, Clontech). The transformants were first selected on αTrp-αLeu-αHis medium with 2 mM 3-aminotriazol (AT) and then on αTrp-αLeu-αAde medium [38]. *pACT2* vectors were isolated from each positive yeast colony to determine the interacting protein encoded by the sequence fused with GAL4 AD.

Reconstructions were performed in yeast using identical media by transforming *pACT2* or a *pGAD424* recombinant vector in combination with the *pGBT9* constructs. No yeast colonies were obtained using *pGBT9* vectors expressing GAL4-DBD alone or fused to an unrelated protein (i.e., a phosphatase subunit).

### Mammalian cell cultures and transfections

Mouse C2C12 and human immortalized [35–37] muscle cells were grown as previously described [9, 19] with 10 or 20% FBS. HEK293 cells were grown at 37°C, 5% CO_2_, and 82% humidity in high glucose DMEM, 10% FBS, 1% pen/strep and 1% L-glutamine. For differentiation, cells were seeded on dishes coated with matrigel (BD Biosciences, San Jose, CA), and, the growth and medium was replaced with DMEM high glucose-L-glutamine supplemented with 2% FBS or with 0.5% insulin and 1% apo-transferrin (Sigma, Gillingham, UK).

Human TE671 (Rhabdomyosarcoma) cells or LHCN-M2 immortalized myoblasts [35] were transfected with the indicated vectors using Fugene6 (Roche Diagnostics, Mannheim, Germany) or Lipofectamine 2000 (Invitrogen), respectively, according to the manufacturer’s instructions.

### Co-immunoprecipitation

C2C12 cells (plated at 3 x10^5^ per 75-cm^2^ flask two days before the transfection) were transfected with 20 μg of the *pCIneo*, *pCIneo-DUX1* or *pCIneo-DUX4* [8] plasmids. Whole cell extracts were prepared 24 h later using sonication in 1.5 ml lysis buffer (Tris 10 mM pH 7.4, NaCl 150 mM, Triton X-100 0.1%) followed by centrifugation for 10 min at 13,000 rpm to remove the cell debris. Immunoprecipitation was performed with 800 μg total extract with rabbit polyclonal SB152, TAR13 or 314 (1:100) directed against DUX proteins [4, 9, 39] in 1 ml immunoprecipitation buffer (1x; Amersham Biosciences) at 4°C O/N (overnight) followed by the addition of 10% v:v protein A-Sepharose (80 mg/ml) for 1.5 h at 4°C. After centrifugation, the immunoprecipitate was heated for 5 min at 95°C in Laemmli buffer without reducing agent and centrifuged for 5 min at 16,000 x g. The supernatant and total extracts (without IP) were separated by 12% SDS-PAGE and electrotransferred to a PVDF membrane. The membrane was blocked in PBS-0.2% Tween, 5% BSA, incubated with a monoclonal anti-desmin antibody (1:1,000, clone D33, Dako, Glostrup, Denmark) followed by incubation with a secondary antibody coupled to HRP, and then visualized using the ECL plus Western Blotting Detection System (Amersham Biosciences, Buckinghamshire, UK).

HEK293 cells were transfected (Life Technologies Lipofectamine 2000 transfection reagent) with Myc-DDK-tagged-human serine/arginine-rich splicing factor 9 (*pCMV6*.*SRSF9*, OriGene), Myc-DDK-tagged-human RNA-binding motif protein 3 (*pCMV6*.*RBM3*, OriGene), Myc-DKK-tagged-human LIM and cysteine-rich domains 1 (*pCMV6*.*LMCD1*, Origene) or *AAV6*.*DUX4*.*V5* expression plasmids and harvested 24 h post-transfection. The cells were lysed in buffer containing 137 mM NaCl, 10 mM TRIS-HCl, pH 7.4, 1% NP40, and protease inhibitor (Life Technologies, 87785). Prior to immunoprecipitation, 250 μg cell lysate was incubated with Protein G Plus Agarose (Calbiochem, IP04) suspension for 1 h at 4°C to remove nonspecific binding partners. To immunoprecipitate tagged protein products, the lysate was incubated with V5 antibody agarose-immobilized conjugate (Bethyl Laboratories, S190-119) or Myc antibody agarose immobilized conjugate (Millipore, 16–219) for 16–20 h at 4°C. Immunoprecipitated protein products and subsequent binding partners were analyzed by western blot using anti-V5 (Invitrogen, R961-25), anti-Myc (Invitrogen, R951-25), and anti-C1QBP (Abcam, ab24733).

### *In vitro* Transcription and translation

Radiolabeled DUX1, DUX4, DUX4-t, IPO13 and luciferase (positive control) proteins were produced by transcription/translation (T/T) *in vitro* (TNT Coupled Reticulocyte Lysate system, Promega) according to the manufacturer’s instructions using *pCIneo-DUX1*, *pCIneo-DUX4*, *pCRT7/NT-DUX4-t*, *pGEM7Z-IPO13* or a luciferase vector in the presence of T7 or Sp6 RNA polymerase and 20 μCi L-^35^S-cysteine (Amersham Biosciences, Roosendaal, The Netherlands). To check the T/T efficiency, the products were boiled for 5 min at 95°C in XT sample buffer (Bio-Rad, Hercules, CA) or SDS loading buffer in the presence of a reducing agent (Fermentas, St. Leon-Rot Germany) and analyzed by SDS-PAGE. The gel was incubated for 30 min in the Amplify solution (Amersham Biosciences), air dried and subjected to autoradiography.

### Purification of GST-fusion proteins and GST pull-down assays with T/T products

*E*. *coli BL21-AI* bacteria (Invitrogen) were transformed with the *pDEST15* vectors, and the expression of the GST-fusion proteins was induced one day later (D.O. 0.4) or not (negative control) by L-arabinose 0.2% for 4 h at 25°C (to avoid the formation of inclusion bodies). The bacteria were centrifuged, and the pellet was resuspended in lysis buffer (Cell lysis buffer, Promega) for 1 min in the presence of protease inhibitors and 5 mM DTE. After 3 freeze-thaw cycles, DNase RQ1 (1:100, Promega) was added, and the reactions were incubated for 30 min at room temperature (RT) on a rotator and then centrifuged at 13,000 rpm for 10 min at 4°C. To verify the GST fusion protein production, the supernatant was mixed 1:1 v:v with lysis buffer and then analyzed by SDS-PAGE and Coomassie blue staining (Simply Blue Safe stain, Invitrogen).

GST-fusion proteins were affinity purified with glutathione (GSH)-Sepharose 4B (Amersham Biosciences) or GSH-linked magnetic beads (MagneGST pull-down system, Promega) according to the manufacturer’s instructions. After gentle shaking at RT for 1.5 h, the mixture of GSH-Sepharose 4B and GST-fusion proteins was centrifuged for 2 min at 13,000 rpm, and the pellets were washed 5 times with 1 ml PBS before resuspension in 0.8 ml of PBS. For the GST pull-down assays, the GST-fusion proteins linked to GSH beads were centrifuged at 13,200 rpm for 2 min, and the pellet was suspended in binding buffer in the presence of 40 μl labeled T/T products, 3 μl L-^35^S-cysteine, or nothing (negative control) and incubated with gentle shaking O/N at 4°C. The beads were harvested by centrifugation for 2 min at 13,000 rpm and washed 4 times with binding buffer. The final pellet was boiled in SDS sample buffer containing reducing agents and analyzed by resolution on two parallel SDS-PAGE gels followed by either Coomassie blue staining or autoradiography.

For the assay using GSH-linked magnetic beads, the incubation with bacterial lysates containing GST-fusion proteins was carried out on a rotator O/N at 4°C, and the beads were harvested with a magnet. For the GST pull-down assay, 5 μl of the beads linked to the GST-fusion proteins was incubated with 20 μl of labeled T/T products for 1 h at RT on a rotator. After 5 successive wash steps, the GST proteins were analyzed as above.

### Purification of GST-DUX4 and GST pull-down with cell lysates

Glutathione-S-transferase (GST)-tagged DUX4 (*pGEX-6p-1*) was purified from BL21 (DE3) competent E. coli (Life Technologies). Protein production was induced at an OD_600_ of 0.6–0.8 by IPTG. All purification steps were performed on ice or at 4°C unless otherwise specified. Soluble lysate was batch purified using Glutathione Sepharose 4B (GE Healthcare) in 50 mM HEPES, pH 7.5, 1 M NaCl, 1 mM DTT, supplemented with protease inhibitor (Pierce). Bound protein was eluted by incubating with binding buffer containing 10 mM reduced glutathione for 30 min at 25°C. Additional purification was performed using a HiTrap Heparin HP column (GE Healthcare). Protein was eluted using a linear gradient from 250 mM to 1000 mM NaCl in 50 mM HEPES, pH 7.5, 1 mM DTT. Peak fractions containing GST-DUX4 were pooled and concentrated using a 30 MWCO centrifugal spin column at 4°C. Concentrated DUX4 was supplemented with 10% glycerol, flash frozen and stored at -80°C.

Total protein lysate was isolated from HEK293 and human myoblasts (clone WS236: 15 unaffected bicep from Wellstone Program). Following DNase treatment, 500 μg of total protein lysate was incubated with GST-DUX4 and Glutathione Sepharose 4B (GE Healthcare) equilibrated in binding buffer containing 150 mM NaCl, 5 mM MgCl2, 1 mM DTT, 25 mM Hepes, pH 7.5, 50 μg/μl BSA and protease inhibitor (Pierce) O/N at 4°C. The resin was washed three times with binding buffer. Bound proteins were eluted by incubating at 95°C for 10 min in binding buffer supplemented with NuPAGE LDS sample buffer, then analyzed by SDS-PAGE. Whole lanes were excised from the gel and submitted for tandem mass spectrometry analysis to identify bound proteins. Non-specific binding partners were omitted based on binding partners of purified GST incubated with total protein lysate, and a threshold of 2 detected peptides was applied.

### HaloTag vectors and purification by affinity chromatography

Twenty-four hours after transfection with a HaloTag expression vector, the cells were harvested in PBS and lysed for 15 min in hypertonic buffer (50 mM HEPES pH 7.5, 500 mM NaCl, 0.5 mM EDTA, 0.005% Igepal and protease inhibitors). Cellular extracts were incubated with the HaloLink resin (Promega) in binding buffer (50 mM HEPES pH 7.5, 150 mM NaCl, 0.5 mM EDTA, 0.005% Igepal and protease inhibitor) for 96 h under rotation at 4°C. The binding buffer and incubation time were optimized following Manufacturer’s instructions and various tests. Following 4- or 24-h incubation, the quantity of HaloTag-proteins was very low on the resin with the majority found in the flow-through. The use of 50 mM DTT in the buffer allowed detection of GFP but not of DUX partners. Several cysteine residues occur in DUX sequences and their involvement in disulfide bridges could be essential for protein folding and interaction with some partner. Protease inhibitors and low temperature were used to avoid protein degradation. EDTA was also added to inhibit metalloproteases and to assist TEV cleavage. IGEPAL, a non-ionic detergent, was used in low concentration to prevent the resin from sticking to plasticware, reduce non-specific binding and increase protein recovery. We eliminated this detergent in the cleavage buffer to be compatible with later mass spectrometry analysis. The resin beads with the bound HaloTag proteins and partners were pelleted and washed five times with wash buffer (50 mM HEPES pH 7.5, 150 mM NaCl, 0.5 mM EDTA and protease inhibitors). Finally, the proteins and their partners were released from the HaloTag by cleavage with the TEV enzyme itself fused to a HaloTag for easy removal. To evaluate the complexity of the pulled-down proteins, we performed an electrophoresis and silver stain of purified complexes (e.g. in S1 Fig) The purified protein extracts containing DUX4, DUX4q, DUX4c or GFP were reduced with 25 mM DTT for 30 min at 60°C and alkylated with iodoacetamide (100 mM) for 30 min at 25°C in the dark. The proteins were digested with 0.1 μg of trypsin O/N at 37°C. The resulting peptides were submitted to LC-MS/MS on an HCT Ultra (Brüker Daltonics, Brussels, Belgium) as previously described [40], and the corresponding proteins were identified using the MASCOT search engine and UNIPROT restricted to human entries.

Proteins interacting with the GFP controls were removed from our analyses, and a threshold of 2 detected peptides was applied. For classification into functional categories, the putative partners were analyzed using bioinformatic tools, including the Database for Annotation, Visualization and Integration (DAVID) and UniProtKB.

### Immunofluorescence

Immunodetection was performed using standard procedures [19]. Briefly, the cells were fixed in 4% paraformaldehyde in PBS (PAF), permeabilized with 0.5% Triton X-100 in PBS, and the nonspecific sites were blocked with 20% FBS in PBS. The cells were subsequently incubated with the appropriate primary antibodies (or the preimmune serum or a non-immunogenic serum as a negative control) for 2 h at RT or O/N at 4°C. The cells were washed with PBS, incubated with the appropriate Alexa-fluor secondary antibodies (Invitrogen), washed again, and mounted with Vectashield mounting medium containing DAPI (Vector Laboratories, Burlingame, Biosciences). The antibodies were diluted in PBS containing 0.5% BSA. The primary antibodies used in this study were rabbit anti-DUX4c serum affinity-purified against an immunogenic peptide (1:50, [19]), mouse [DE-U-10] monoclonal anti-desmin (1:50; Abcam, Cambridge, UK), DUX4 antibodies (rabbit 314 or mAb 9A12, [10]), anti-FUS-TLS (4H11 Santa Cruz; 1:500) or anti-SFPQ (6D7 Sigma; 1:500).

The muscle biopsies were frozen in isopentane and stored at -80°C. Six- to ten-micron sections were fixed in 4% PAF, washed 2 times in 0.5% PBS-BSA, and incubated for 5 min with PBS with 0.5% BSA and 0.5% Triton X-100. The sections were incubated O/N at 4°C with primary antibodies against DUX4c (1:20) or desmin (1:50), the preimmune DUX4c serum (1:20) or a non-immunogenic mouse serum (1:50) (negative controls). The sections were washed in PBS containing 0.5% BSA and then incubated for 1 h at RT with Alexa fluor-conjugated secondary antibodies (1:1,000, goat anti-mouse 555 or goat anti-rabbit 488). The sections were mounted with Mowiol 4.88 (Calbiochem, San Diego, CA) and DAPI (1/1,000, D8417, Sigma).

The images were obtained with a Nikon Eclipse 80i microscope (with fluorescence filters of small range allowing detection of very low abundance proteins) or a confocal Nikon microscope (system C1).

### In situ proximity ligation assay (*in situ* PLA)

A total of 1.2 x10^4^ LHCN-M2 cells were seeded in chamber slides (Thermo Fisher Scientific, Villebon sur Yvette, France). Twenty-four hours later, the myoblasts were transfected with the indicated DUX-expression vector using Nanojuice (Novagen, WI, USA) as described in [41]. After 48 h, the cells were fixed in 4% PAF, permeabilized with PBS with 0.5% Triton X-100 and blocked in PBS with 20% FCS (Fetal Calf Serum). The myoblasts were then incubated with primary antibodies against DUX4 (rabbit 314 or mAb 9A12, [10]), DUX4c (1:50), desmin (1:50) or FUS-TLS (4H11 Santa Cruz; 1:500) or SFPQ (6D7 Sigma; 1:500) at 4°C O/N. PLA (Duolink, Olink Biosciences, Uppsala, Sweden) was performed according to the manufacturer’s instructions. Species-specific secondary antibodies (mouse and rabbit) conjugated to oligonucleotides (PLA probes) were used for hybridization followed by the ligation and amplification steps. The cells were then incubated in the dark with detection medium for 2 min at different concentrations (2x, 1x and 0.2x) and rinsed with 70% ethanol. Finally, the cells were mounted with Gold Antifade reagent with DAPI (Invitrogen). Negative controls were generated by omitting the primary antibodies or by using a single antibody.

## Results

### A genetic screen to identify putative DUX1, DUX4 and DUX4c protein-binding partners in skeletal muscle

**Fig 1:**
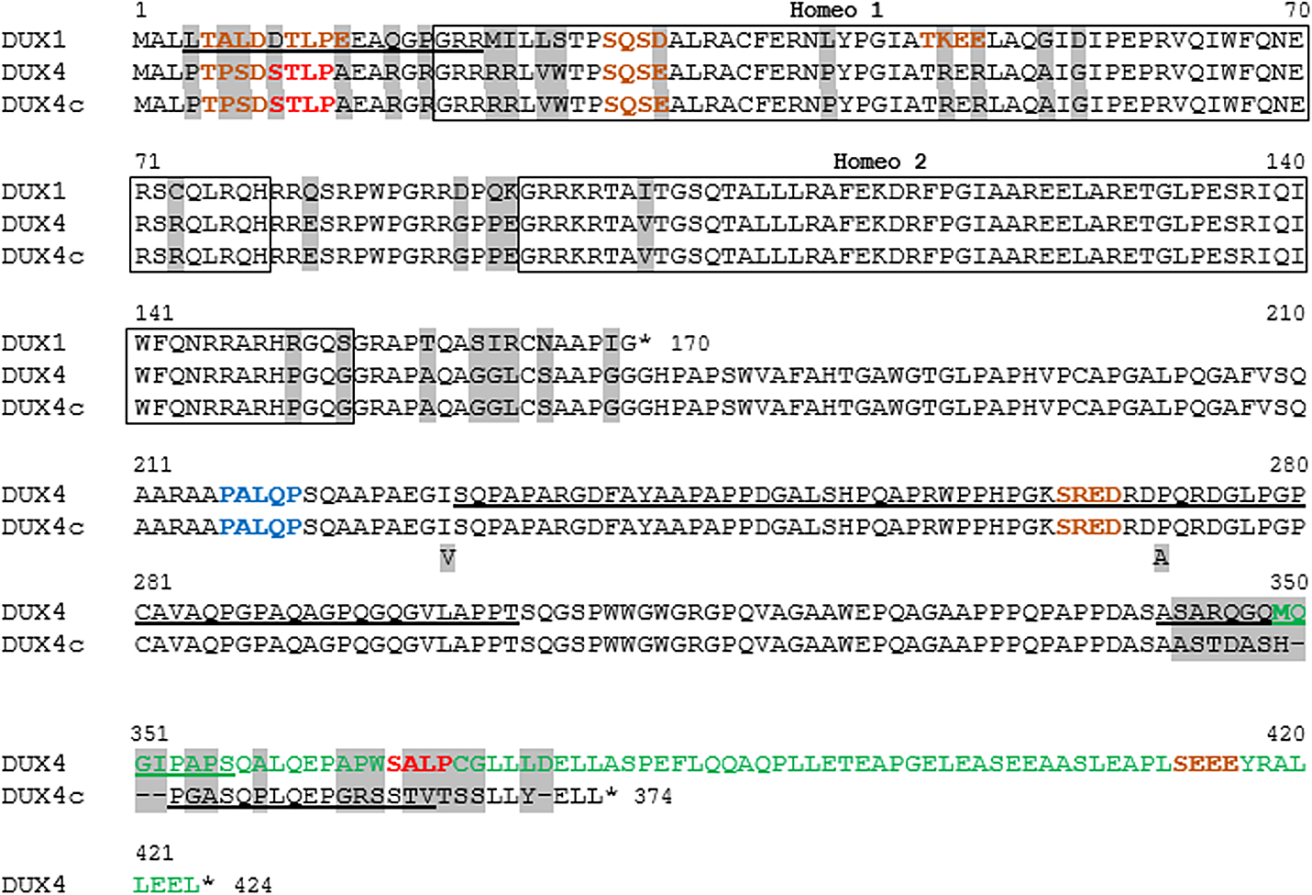
Alignment of the DUX1, DUX4 and DUX4c protein sequences. DUX4c and DUX4 are identical from the NH_2_ terminus to residue 342. The two homeodomains highlighted in boxes and are targets of rabbit TAR13 serum [4]. Residues that differ are highlighted in grey. The positions of putative CKII phosphorylation sites (brown) and putative binding regions for tubulin (red) and of a subset of MYND (blue) proteins (putative DUX partners) are indicated. The region recognized by MAb 9A12 (residues 230–303), which cross-reacts with DUX4 and DUX4c, and the peptides used to generate the specific DUX1, DUX4c or DUX4 rabbit antisera are underlined: the SB152 rabbit serum was directed against DUX1 residues 4 to 21, the 314 rabbit serum was directed against DUX4 residues 342 to 356, and the anti-DUX4c rabbit serum was directed against DUX4c residues 351 to 366. The DUX4tail (DUX4-t) and DUX4term (green residues) domains map to residues 172–424 and 349 (alternative initiator methionine)-424, respectively. Two DUX4c polymorphisms are indicated [19].

To better understand the function of the DUX proteins, we searched for their protein partners in a human skeletal muscle cDNA library using the yeast-two-hybrid method [42]. We investigated DUX1, which is limited to the double homeodomain, and the very similar DUX4 and DUX4c proteins, which contain an additional carboxyl terminal domain. The carboxyl terminal domain of DUX4c is 50 residues shorter than that of DUX4 and differs from DUX4 in its last 32 residues (Fig 1). The DUX proteins were expressed as fusions to the GAL4 DNA-binding domain. DUX4 had a very strong transcriptional activity, precluding its use as a bait in yeast strain pJ69-4 A. In contrast, neither the DUX4c nor DUX1 have such an activity ([6] and data not shown). However, using the AH109 yeast strain containing a weaker GAL4 responsive element allowed the use of DUX4 as a bait.

PJ69-4A yeast cells were co-transformed with a *GAL4DBD-DUX* expression vector and a second vector expressing the GAL4 activation domain fused to a protein encoded by a human skeletal muscle *cDNA* library.

A total of 10^5^ clones from the cDNA library were screened using DUX1 as a bait, yielding 68 positive clones. The nucleotide sequence of the cDNAs present in 42 positive clones could be determined, and 35 of these sequences corresponded to portions of the desmin cDNA. Reconstruction experiments showed that the shortest desmin protein fragment expressed from our positive cDNA clones (amino acids 260–470, corresponding to the COIL2 α-helical segment and tail domains) could interact with a single DUX1 homeodomain 1 (S2 Table). Other confirmed clones corresponded to alpha-actinin-2 and to a subunit of casein kinase II (Table 1).

**Table 1:** Putative DUX1 protein partners based on Y2H analysis.

Number of hits	Genes/encoded proteins	In frame?	Reconstruction/note
35	Desmin	Yes	+
1	Actinin, alpha 2, ACTN2	Yes	+
1	Actinin, alpha 3, ACTN3	Yes	-
1	Casein kinase 2, beta polypeptide, CSNK2B	Yes	+
1	Homo Sapiens centromere protein E, CENP-E	Yes	-Tubulin-associated protein
1	HADHB	No	RNA-binding protein

nd: out-of-frame sequence that may be a result of difficult sequence products or to a translational frameshift of the GAL4AD-fused protein as previously reported in a Y2H screen [43].

The screening of this library (2.8 x 10^5^) using DUX4c as a bait yielded 187 positive clones. Out of the 150 positives clones analyzed, we identified 105 as containing a desmin cDNA fragment.

Different putative DUX4c partners were further identified in the following categories: (i) cytoskeletal proteins (as alpha-actinin-3, actin, myosin), (ii) transcription factors with a zinc finger domain of either the LIM or MYND type, (iii) RNA-binding proteins, including splicing factors. Another positive clone encoded importin/karyopherin 13 (IPO13) (Table 2).

**Table 2:** Putative DUX4c protein partners based on Y2H analysis.

Number of hits	Genes/encoded proteins	In frame?	Rec. /note	Accession number	E-value, Base pair identity
**1. Myofibril/cytoskeleton-associated proteins**					
105	Desmin	Yes	RNA- & MyoD- binding *#	AF521879	0.0
13	Four and half LIM-3, FHL3	Yes	+ MyoD-binding	BC001351.2	0.0
1	LIM-like protein, LIMS2/PINCH2	n.d.		BC065816.1	3e-119 (406/484)
3	Actinin, alpha 3, ACTN3	Yes		NM_001258371.1	0.0
2	Actin, Alpha-1, ACTA1	No	§	NM_001100.3	2e-72 (213/239)
1	Troponin C type 2 (fast), TNNC2	No		BC005323.1	0.0
1	Myosin, heavy chain 2, MYH2	No	§	NM_017534.5	0.0
1	Synaptopodin 2, SYNPO2	No		NM_001128933.2	0.0
1	Glypican 1, GPC1	No	3’UTR	NM_002081.2	6e-139 (390/444)
**2. RNA-binding proteins and splicing factors**					
2	Zing finger (MYND)	Only	+	BC094693.1	0.0
	domain, ZMYND17	MYND			1e-51 (137/150)
1	Splicing factor 1, SF1	Yes		XM_006718685	0.0
1		No			3e-89 (269/309)
1	DEAD box helicase 5, DDX5	Yes	*	NM004396.3	0.0
1	Complement component 1, q subcomponent-binding protein, C1QBP, SF2 P32 subunit	No	*	NM_001212.3	3e-89 (192/197)
1	RNA-binding motif protein 24, RBM24	n.d.	*MYOD* stability §	NM_001143941.1	1e-62 (192/227)
1	Ribosomal protein L4, RPL4	No	*	NM_000968.3	0.0
**3. Others**					
3	Importin 13, IPO13	Yes	+	BC008194.1	0.0
			#		
3	Voltage-Dependent Anion	No	*	NM_001184823.1	0.0
	Channel 2, VDAC2			BC012883.1	
3	Creatine kinase, CKM	No		BC007462.1	0.0
2	Co-enzyme Q10 homolog B, COQ10B	No		NM_025147.3	0.0
1	NDUFB10	Yes	-	NM_004548.2	0.0
1	Phosphatase 1, catalytic subunit, beta isozyme, PPP1CB	No		NM_206876.1	0.0
1	LOC102725482	No	ncRNA	XR_425709.1	8e-43 (121/134)

n.d.: not determined due to poor sequence quality; an out-of-frame sequence may be a result of difficult sequencing products, such as ZMYND17, for which only the MYND region was found in frame, or a translational frameshift of the GAL4AD-fused protein [43].

* also identified as DUX4 or DUX4c partner by Y2H or GST pull-down/co-immunoprecipitation (see Table 3 or S3 and S4 Tables)

^§^ Isoform (or similar function) identified in other approaches (see Tables 2 and 3 or S3 and S4 Tables)

# Validated interaction by *in situ* PLA in human muscle cells

The screening of a mouse embryonic cDNA library using DUX4 as bait also identified desmin as a putative partner. Other putative DUX4 partners included: (i) cytoskeletal proteins (such as myofibril- and microtubule-associated proteins, a LIM-containing protein), (ii) RNA-binding proteins such as C1QBP (also found as a putative DUX4c partner), serine/arginine-rich splicing factor 9 (SRSF9; a known C1QBP interactor) [44] and RNA-binding motif protein 3 (RBM3). RBM3 contains a RRM (RNA recognition motif) domain, similar to that found in RBM24. We found RBM24 in the screen with DUX4c, as well as RPL4, which was previously shown to be a RBM3 interactor [45]. Some of the DUX4 partners are involved in transcription, protein folding or oxidative stress. Others are principally known as extracellular or membrane proteins or involved in vesicle trafficking (Table 3).

**Table 3:** Putative DUX4 protein partners based on Y2H analysis.

Nb of Hits	Genes/encoded proteins	In frame?	Rec./note	Accession number	E-value (Base pair identity)
**1. Cytoskeletal proteins**					
	**Myofibril-associated**				
46	Desmin, Des	Yes	#	NM_010043.2	0.0 (928/928)
1	Thyroid hormone receptor interactor 6, Trip6	Yes		NM_011639.3	0.0 (702/704)
1	adenylate cyclase-associated protein 1, Cap1	Yes		NM_001301067.1	0.0 (661/677)
1	phosphatidylinositol 4-kinase type 2 alpha, Pi4k2a	Yes		NM_145501.2	0.0 (909/931)
1	LIM and cysteine-rich domains 1, Lmcd1	Yes	# Z-lineprotein	NM_144799.2	0.0 (807/820)
	**Microtubule-associated**				
1	tumor protein, translationally-controlled 1, Tpt1	Yes		NM_009429.3	0.0 (796/797)
1	centromere protein A, Cenpa	Yes		NM_001302132.1	0.0 (957/965)
1	Tubulin, gamma complex associated protein 6, Tubgcp6	Yes		NM_001163319.1	0.0 (902/913)
**2. RNA-binding proteins and splicing factors**					
10	RNA-binding motif protein 3, Rbm3	Yes	#	NM_001293658.1	0.0 (755/775)
6	Complement component 1, q subcomponent binding protein, C1qbp	Yes	# * and **	NM_007573.2	0.0 (902/905)
2	Splicing factor, arginine/serine rich 9, Sfrs9	Yes	#	NM_025573.3	0.0 (900/907)
1	MIF4G domain containing, Mif4gd	Yes		NM_001243587.1	0.0 (854/860)
1	EP300-interacting inhibitor of differentiation, Eid1	Yes	Repress MYOD1 transact.	NM_025613.3	0.0 (900/904)
**3. Transcription-Elongation**					
5	Histone acetyltransferase 1, Hat1	Yes	#	NM_026115.4	0.0 (907/924)
3	heart and neural crest derivatives-expressed transcript 2, Hand2	Yes		NM_010402.4	0.0 (909/915)
1	polybromo 1, Pbrm1	Yes		NM_001081251.1	0.0 (955/958)
1	RNA polymerase II DNA-directed polypeptide G, Polr2g	Yes		NM_026329.2	0.0 (813/815)
1	Topoisomerase II alpha, Top2A	Yes		NM_011623.2	0.0 (629/634)
**4. Extracellular and membrane proteins**					
6	EGF-containing fibulin-like ECM protein 1, Efemp1	Yes		NM_146015.2	0.0 (941/945)
4	Placenta-specific 8, Plac8	Yes		NM_139198.2	0.0 (522/522)
4	fibronectin 1, Fn1	Yes		NM_001276410.1	0.0 (893/905)
2	Lymphocyte antigen 6 complex, Ly6a	Yes		NM_001271446.1	6e-139 (271/271)
2	Fibrillin 1, Fbn1	Yes		NM_007993.2	0.0 (917/927)
1	Prosaposin, Psap	Yes		NM_001146124.1	0.0 (920/935)
1	insulin-like growth factor binding-protein 3, Igfbp3	Yes		NM_008343.2	0.0 (883/911)
1	Matrillin 2, Matn2	Yes		NM_016762.2	0.0 (725/738)
1	lysyl oxidase like 1, Loxl1	Yes		NM_010729.3	0.0 (922/931)
1	Latent transforming growth factor beta-binding protein 3, Ltbp3	Yes		NM_008520.2	0.0 (910/912)
1	Fibulin 2, transcript variant 1, Fbln2	Yes		NM_007992.2	0.0 (945/953)
**5. Protein folding**					
1	peptidylprolyl isomerase B, Ppib	Yes		NM_011149.2	0.0 (915/923)
**6. Oxidative stress**					
1	Glutathione peroxidase 3, Gpx3	Yes		NM_008161.3	0.0 (912/925)
**7. Ribosomal proteins**					
1	Ribosomal protein, Large P2, Rplp2	Yes		NM_026020.6	4e-106 (212/212)
**8. Others**					
1	RIKEN cDNA 2610042014 gene (SYS1 Golgi-localized integral membrane protein homolog (S. cerevisiae) (Sys1),)	Yes		NM_025575.3	0.0 (779/785)
1	guanine nucleotide-binding protein (G protein), gamma 12, Gng12	Yes		NM_025278.5	0.0 (601/612)
1	expressed in non-metastatic cells 1, protein, Nme1	Yes		NM_008704.2	0.0 (675/680)
1	Morf5 family associated protein 1, Mrfap1	Yes		NM_026242.3	0.0 (914/929)
1	proteolipid protein 2, Plp2	Yes		NM_019755.4	6e-40 (93/93)
1	DNA segment, Chr 14, ERATO Doi 449, expressed, D14Ertd449e (transmembrane protein 254b (Tmem254b))	Yes		NM_001270496.1	0.0 (716/716)
1	myeloid leukemia factor 2, Mlf2	Yes		NM_001170341.1	0.0 (897/906)
1	chromosome 8 genomic contig, strain C57BL/6J (F6) (Mus musculus predicted gene 2694 (Gm2694), transcript variant 2, long non-coding RNA)	Yes		NR_125722.1	3e-127 (255/257)
1	methylmalonic aciduria (cobalamin deficiency) cblD type, with homocystinuria, Mmadhc	Yes		NM_133839.2	0.0 (929/948)
1	LON peptidase N-terminal domain and ring finger 1 (Lonrf1)	Yes		NM_001081150.1	0.0 (883/886)
1	SHD-domain GRB2-like endophilin B1, Sh3glb1	Yes	May promote membr. fusion	NM_001282042.1	0.0 (937/945)
1	Nuclear factor of kappa light chain gene enhancer in B-cell inhibitor, Nfkbia	Yes		NM_010907.2	0.0 (591/592)
1	dystrophia myotonica-containing WD repeat motif, Dmwd	Yes		NM_010058.2	0.0 (462/464)

# Validated interaction by co-immunoprecipitation in HEK293 and C2C12 cells and by GST pull-down

* also identified by GST pull-down and subsequent MS analysis

** also identified by HaloTag-DUX4 co-purification

Several clones were confirmed by reconstruction experiments in yeast (Tables 2 and 3). These experiments did not reveal interactions between desmin fragments and any of the two individual DUX4/4c homeodomains (S2 Table). However, desmin was identified as the most frequent protein partner of full-length DUX4 and DUX4c (see above), which harbor identical double homeodomains, suggesting that both homeodomains are necessary for this interaction.

### DUX4 interacts with desmin and karyopherin/importin 13

**Fig 2:**
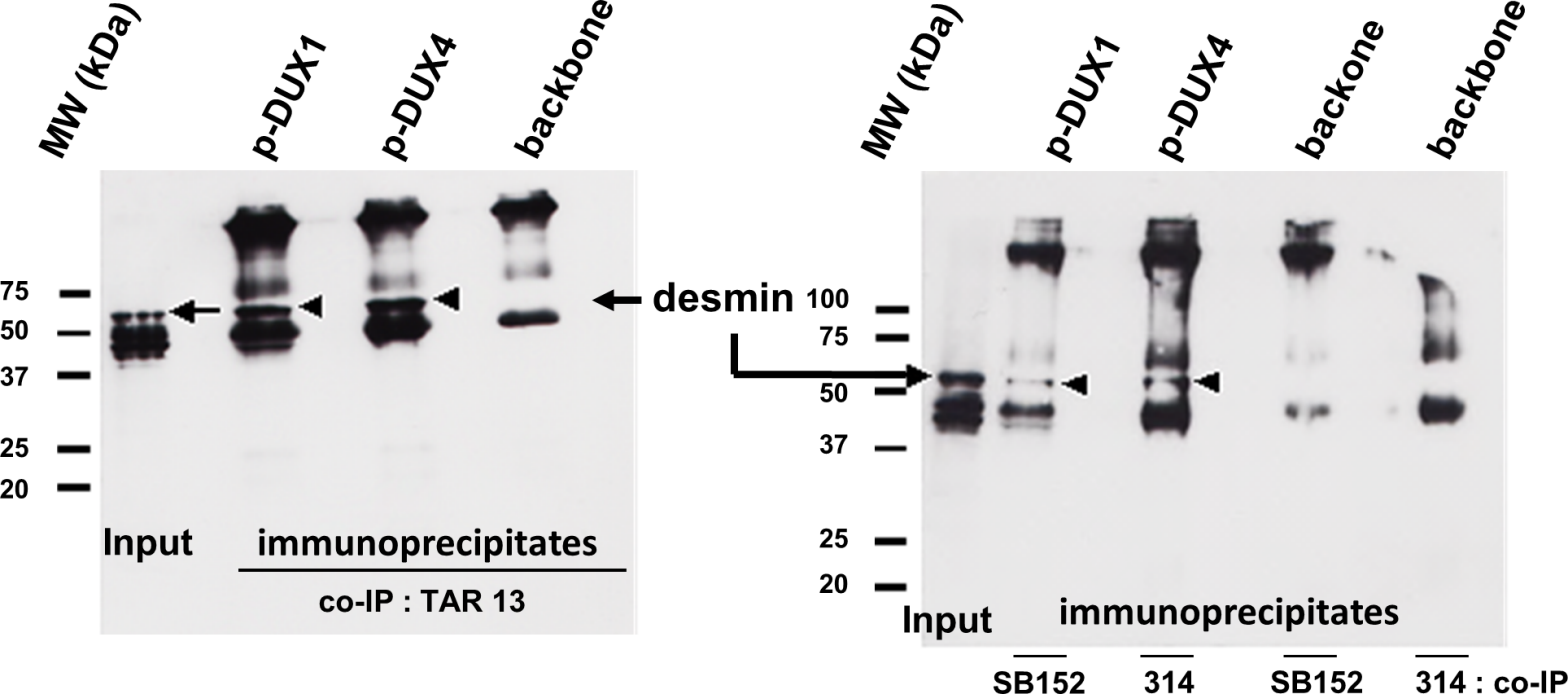
Co-immunoprecipitation of DUX proteins and desmin. C2C12 cells were transfected with the *pCI-neo* expression vector for either DUX4 or DUX1 or with the backbone vector. Total cell lysates were prepared 24 h later and incubated with rabbit sera raised against the double homeodomain common to DUX1 and DUX4 (TAR13) or the amino-terminal domains of DUX1 (SB152) or DUX4 (314). Samples of input (total extracts of cells transfected with the backbone vector) and the immunoprecipitates were separated by SDS-PAGE and electrotransferred to a PVDF membrane for Western blotting. Desmin was detected using a specific antiserum (see Methods for details).

Because we did not expect to identify cytoplasmic partners for this family of nuclear proteins, we wanted to validate the putative interaction between desmin and the DUX proteins using different approaches. We first performed a co-immunoprecipitation assay. Mouse C2C12 myoblasts were transfected with a DUX expression vector or the empty parental vector. One day later, total cell extracts were prepared and incubated with a rabbit serum against the DUX proteins, and the immunoprecipitated proteins were separated by SDS-PAGE and subjected to western blotting. A specific desmin antibody allowed the visualization of a 53-kDa protein only in cells transfected with the DUX expression vector (Fig 2).

We then expressed desmin fused to GST or GST alone in *E*. *coli*. Radiolabeled DUX proteins or luciferase (a negative control) were produced by *in vitro* transcription/translation (T/T; rabbit reticulocyte lysate) in the presence of [^35^S]-cysteine and incubated with purified GST-desmin (or GST as a negative control) bound to GSH-Sepharose. After centrifugation and washing, the GST pull-down products were analyzed by SDS-PAGE followed by Coomassie blue staining (S2A Fig) or by autoradiography (S2B Fig). GST and GST-desmin were detected at 28 and 80 kDa, respectively (S2A Fig). Radioactive DUX1 (20 kDa) and DUX4 (52 kDa) were detected in the GST pull-down products (S2B Fig), confirming their interaction with desmin. DUX4-t, corresponding to the last 255 residues of DUX4 (Fig 1), was not detected in the GST pull-down products, suggesting that the interaction with desmin was mediated by the DUX homeodomain(s).

By a similar procedure, GST-DUX4 and -DUX4c in parallel with negative controls were expressed in *E*. *coli*, affinity purified using GSH-linked magnetic beads, and incubated with radiolabeled IPO13 to confirm the interaction between DUX4 or DUX4c and IPO13 (S3A Fig). Moreover, we detected an IPO13-DUX4/4c interaction at the nuclear periphery of differentiating myoblasts using the *in situ* proximal ligation assay (PLA, see Methods) allowing for the direct observation of co-localized proteins that appear as a single red fluorescent spot [46] (S3B Fig).

### DUX4 interacts with RNA-binding proteins involved in splicing, mRNA export and translation

**Fig 3:**
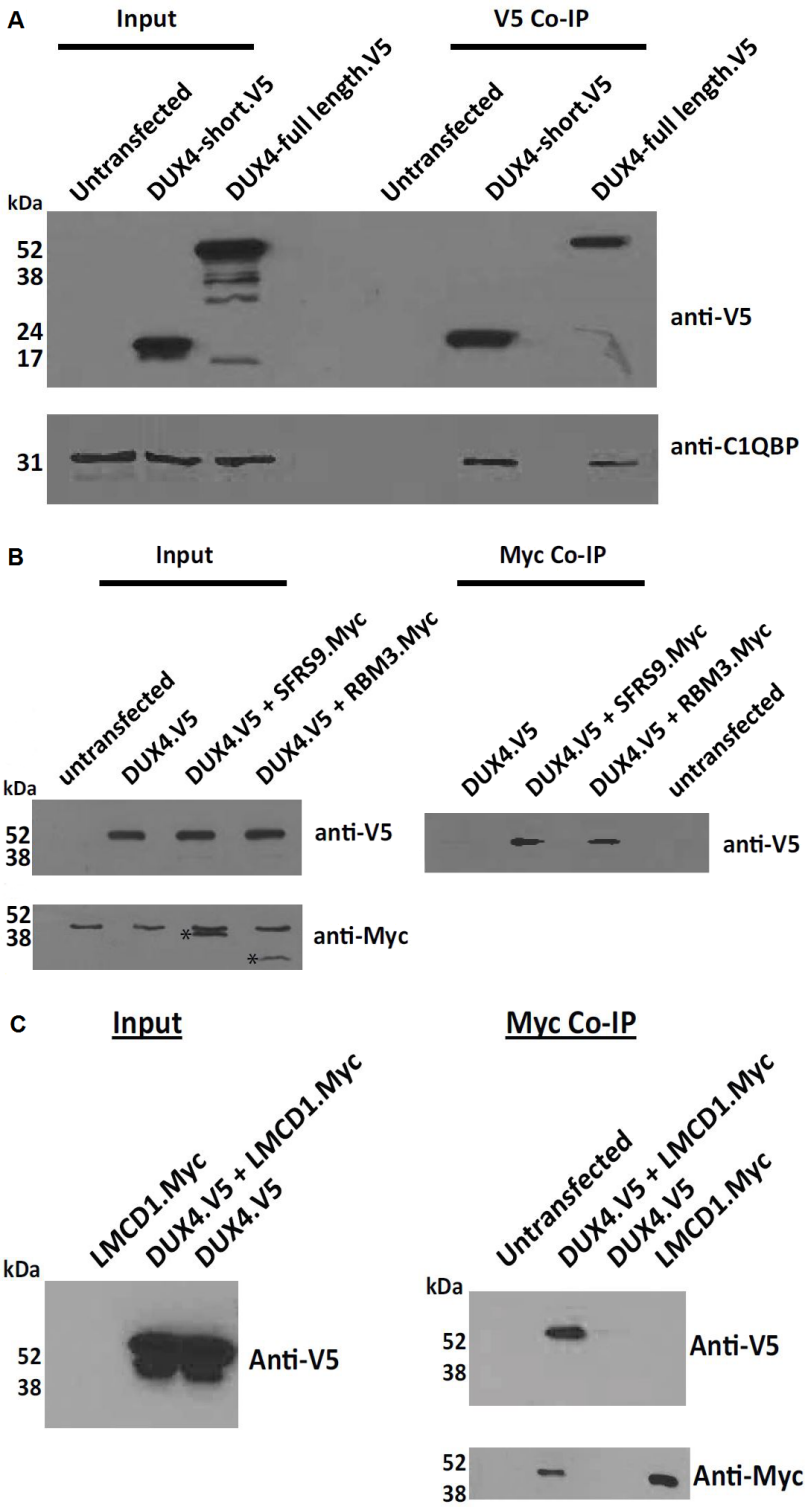
Co-immunoprecipitation of DUX4 with RNA-binding proteins. (A) HEK293 cells were transfected or not (untransfected) with plasmids expressing V5 epitope-tagged DUX4 (full length) or DUX4 limited to the homeodomains (short). Cell protein extracts before (input) or after immunoprecipitation with anti-V5 antibodies (V5 Co-IP) were analyzed by SDS-PAGE, transferred to a western blot and immunoblotted with either anti-V5 (top panel) or anti-C1QBP antibodies (bottom panel). (B) HEK293 cells were transfected with expression vectors for DUX4-V5 as in A, or for Myc epitope-tagged RBM3 and SFRS9 as indicated. Cell protein extracts before (input) or after immunoprecipitation with antibodies against Myc (Myc Co-IP) were analyzed as above with antibodies against either V5 (top panel) or Myc (bottom panel). The results indicate that DUX4 co-immunoprecipitated with SFRS9 and RBM3. (C) HEK293 cells were transfected with expression vectors for DUX4-V5 as in A or expression vectors for Myc epitope-tagged LMCD1 as indicated. Cell protein extracts before (input) or after immunoprecipitation with antibodies against Myc (Myc Co-IP) were analyzed as above with antibodies either against V5 (top panel) or Myc (bottom panel). The result indicate that DUX4 co-immunoprecipitated with LMCD1. The input analysis was on 1/16th of lysate used for the immunoprecipitation experiments.

To validate a number of interactions of DUX4 with the RNA-binding proteins identified above, we transfected human HEK293 cells with a vector expressing either full length DUX4 or DUX4 limited to the homeodomains (residues 1–160, short DUX4) tagged at the carboxyl-terminus with a V5 epitope. The cells were lysed 24 h later and incubated with a V5 antibody. The immunoprecipitated proteins were separated by SDS-PAGE and subjected to western blotting. Incubation of the membrane with an anti-V5 antibody showed DUX4 expression only in transfected cells. Using a C1QBP-specific antibody, we then confirmed DUX4 interaction with endogenous C1QBP present in HEK293 cell extracts. We found that C1QBP was immunoprecipitated only in HEK293 cells transfected with DUX4 but not in untransfected controls that lacked DUX4 expression. Moreover, the DUX4 homeodomains were sufficient to mediate this interaction (Fig 3A) and DNA was not essential for the interaction because the DUX4 mutant defective in DNA binding (Homeodomain I; [47]) could still associate with C1QBP (S3C Fig).

Similar experiments were performed using HEK293 cells transfected with a *DUX4-V5* expression vector as above or co-transfected with vectors expressing myc-tagged SFRS9 (official name SRSF9) or RBM3. The immunoprecipitations with either anti-myc or anti-V5 antibodies confirmed a DUX4 interaction with both proteins, validating these interactions (* in Fig 3B).

### DUX4 interacts with LIM and cysteine-rich domain 1 protein (LMCD1) involved in cardiac hypertrophy

We also validated the interaction of DUX4 with LMCD1 using HEK293 cells transfected with a DUX4-expression vector or co-transfected with vectors expressing myc-tagged LMCD1, as described above. Cell extracts were immunoprecipitated with either anti-Myc or anti-V5 antibodies followed by analysis by SDS-PAGE, Western blotting and immunodetection with antibodies against either V5 or Myc tags to validate this interaction (Fig 3C).

### Protein co-purification from muscle cells extends the range of putative DUX4- and DUX4c-binding partners

To identify DUX protein-binding partners directly in mammalian cells, we used the HaloTag technology. We constructed expression vectors for fusion proteins of the HaloTag (either amino- or carboxyl-terminal) with either DUX4, DUX4c or the last 76 DUX4 residues (DUX4term). An EGFP-HaloTag fusion protein was used as a negative control. We transfected human muscle cells with each expression vector. The cells were lysed the next day, and the HaloTag-fusion proteins were purified by covalent capture on HaloLink resin. The co-purified protein partners were digested with trypsin, and the resulting peptides were identified by LC-MS/MS analysis.

This approach again confirmed the interaction between DUX4 and desmin as well as C1QBP. We found C1QBP in the HaloTag-DUX4c immunoprecipitates. However, surprisingly no interaction between desmin and HaloTag-DUX4c was observed. The major identified proteins were classified into different functional groups (S3 Table). Some of these had been previously identified by Y2H using DUX4 or DUX4c as a bait (Table 2), including (i) proteins involved in myofibrillar and cytoskeletal organization, such as actin- and tubulin-interacting proteins, actin 2 and most of the subunits of the TCP1 complex, and (ii) transcription factors and RNA-associated proteins. Among these partners, desmin, α-actinin, actin, myosin, ribosomal proteins and splicing factors such as DDX5, and C1QBP, as well as other members of the RBM, DDX and SRSF splicing factors families, were previously identified by the Y2H screen (Tables 2 and 3, S3 and S4 Tables).

**Fig 4:**
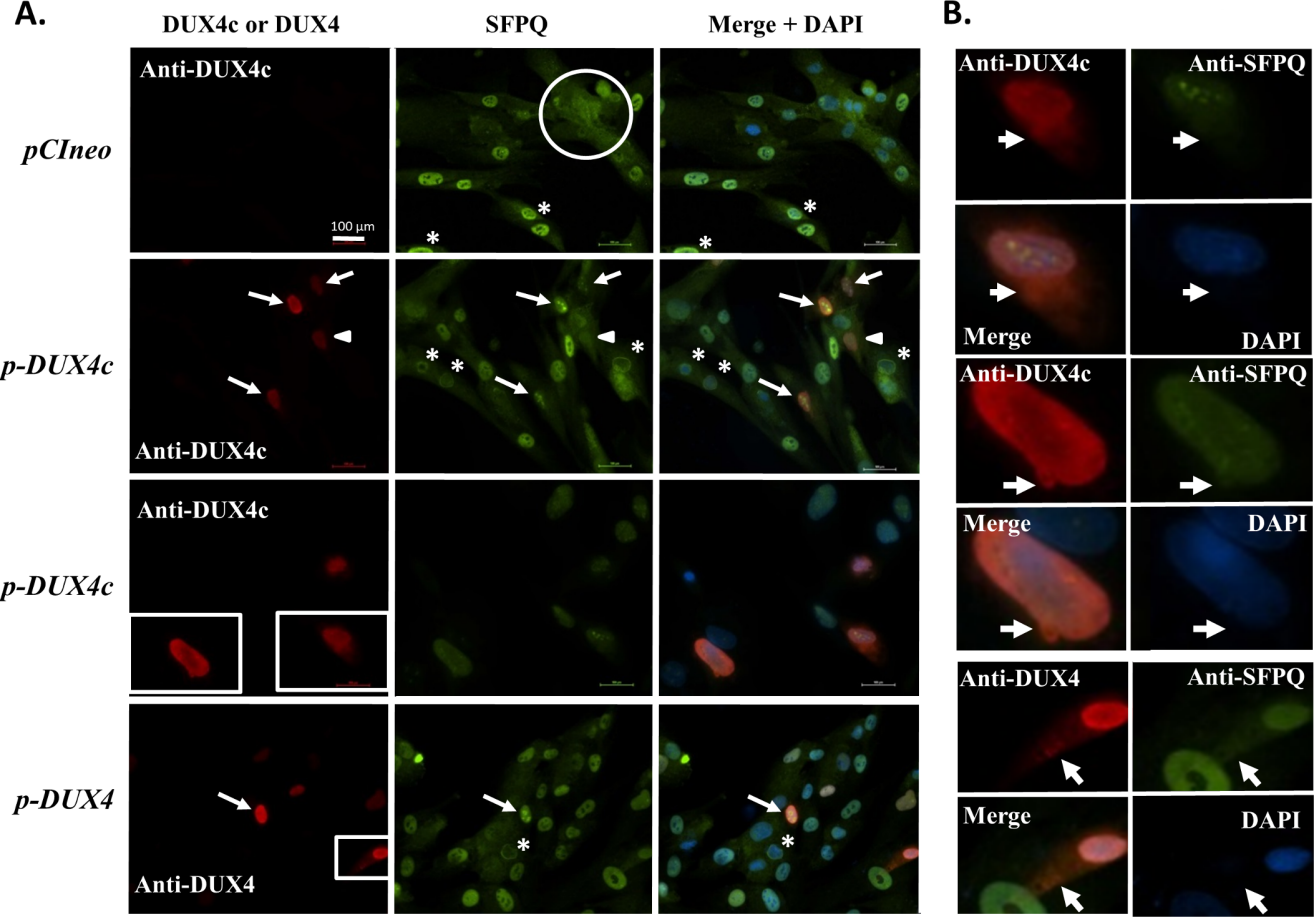
Nuclear and cytoplasmic (co-)localization of DUX4 or DUX4c with SFPQ in myoblasts. LHCN-M2 cells were transfected with a DUX4c or DUX4 expression vector (*p-DUX4*, *p-DUX4c*) or the backbone vector (*pCIneo*). (**A**) In the cells transfected with the backbone vector (top panels), SFPQ detected by immunofluorescence (green) was localized either in the nuclei, excluding the nucleoli, with sometimes a major presence at the periphery (*) or in the entire cell (circle). In DUX4c-overexpressing cells (red), SFPQ was delocalized inside the nuclei and appeared in approximately 3 large spots (arrows) or seemed present in the entire cell (arrowhead) (middle panels). DUX4-overexpressing cells (red) are shown in the bottom panels with a similar SFPQ delocalization inside the nuclei in one DUX4-overexpressing cell. Boxed regions are magnified in B. (**B**) Arrows highlight cytoplasmic localization of DUX4c, DUX4 or SFPQ, sometimes observed as emerging from the nuclei.

**Fig 5:**
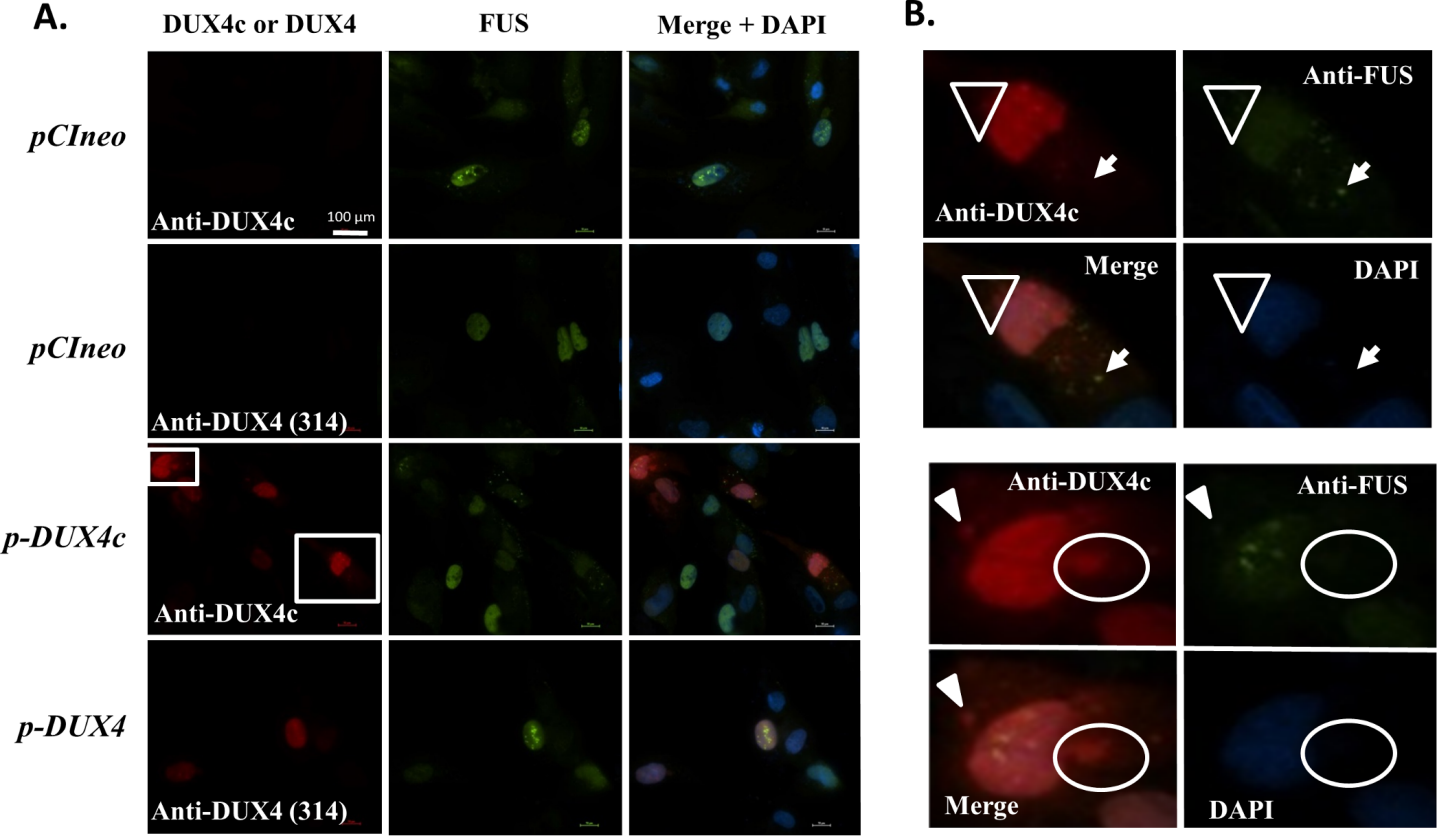
Nuclear and cytoplasmic (co-)localization of DUX4 or DUX4c with FUS in myoblasts. LHCN-M2 cells were transfected with a DUX4c or DUX4 expression vector (*p-DUX4*, *p-DUX4c*) or the backbone vector (*pCIneo*). (**A**) In the cells transfected with the backbone vector (upper panels), FUS detected by immunofluorescence (green) was localized principally in the nuclei but also in the cytoplasm. In DUX4c- (middle panels) and DUX4- (bottom panels) overexpressing cells (red), nuclear and cytoplasmic FUS was generally observed. (**B**) Magnified boxed regions in A. Partial co-localization of cytoplasmic DUX4c or DUX4 with FUS are highlighted by triangles or arrowheads and was sometimes observed as emerging from the nuclei (circles). Arrows point to cytoplasmic spots of FUS.

**Fig 6:**
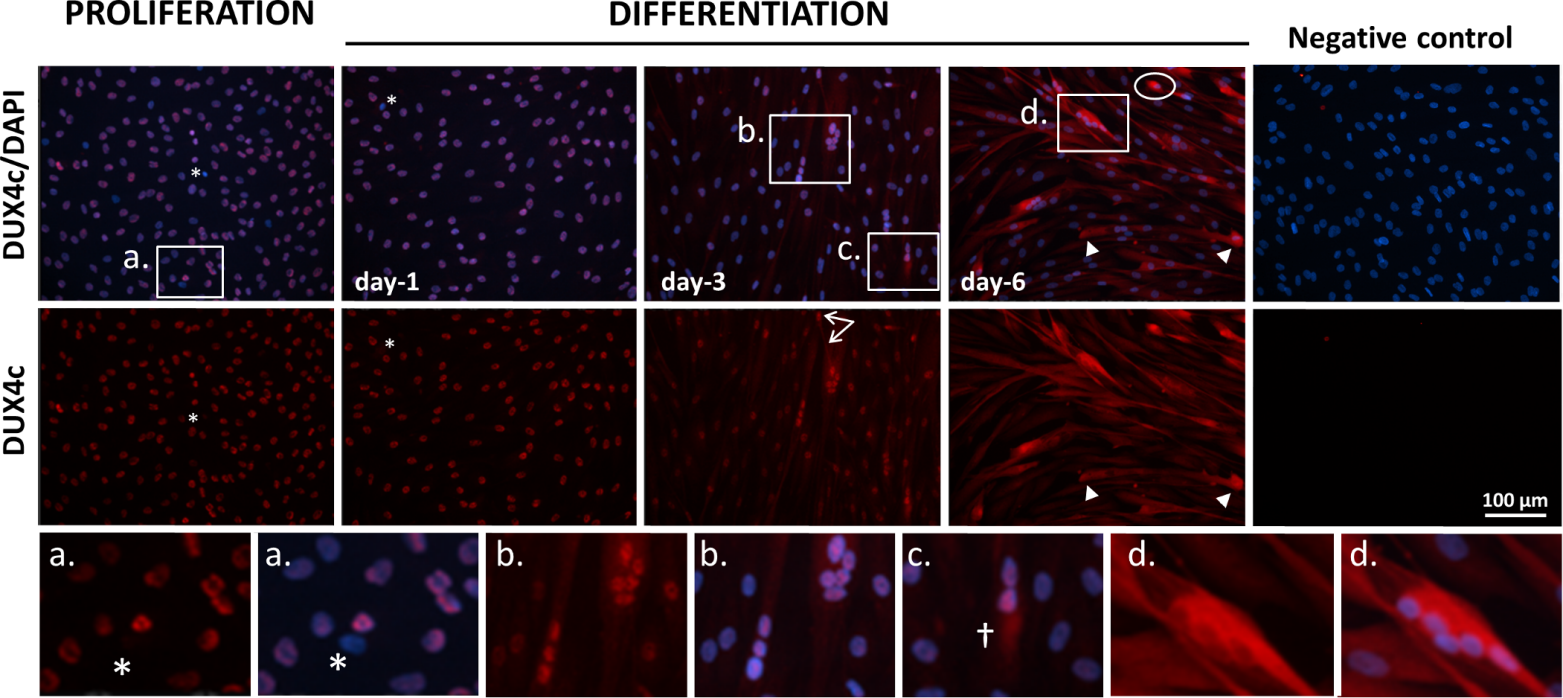
Cytoplasmic detection of endogenous DUX4c in differentiating healthy myoblasts. DUX4c was detected by immunofluorescence (red) in proliferating immortalized myoblasts and during a differentiation time-course. During proliferation and after one day of differentiation, nuclear staining was observed in almost all myoblasts. However, the few nuclei with intense DAPI staining did not present DUX4c immunofluorescence (asterisks). In myoblasts, DUX4c was detected in the nucleoplasm or as 1 to 4 spots at the nuclear periphery (**a**). During differentiation, DUX4c began to be detected in the cytoplasm, and its nuclear labeling decreased. However, stronger nuclear DUX4c staining was still observed in myotubes containing at least 3 nuclei at day 3 (**b**) and in a myoblast presenting a cytoplasmic extension towards a myotube (arrows). In addition, a few muscle cells showed higher cytoplasmic DUX4c staining (the cross in **c**). At day 6, the nuclear staining was completely lost, and some myotubes (**d**) or myoblasts (circles) contained strong cytoplasmic staining, mostly on one side of the cell. Some myotube tips were also stained (arrowheads).

By co-immunofluorescence, we also observed a partial co-localization of the FUS (also known as TLS) and SFPQ splicing factors with DUX4 or DUX4c in DUX4- or DUX4c-overexpressing myoblasts (Figs 4 and 5). Surprisingly, we found not only nuclear but also cytoplasmic staining for all these proteins (see below). *In situ* PLA allowing direct observation of co-localized proteins confirmed an interaction principally in the nuclei for DUX4/FUS, but intriguingly mostly in the cytoplasm or around the nuclei for DUX4/SFPQ and DUX4c/FUS or DUX4c/SFPQ (S4 Fig). Moreover, FUS or SFPQ interaction with DUX4c was more frequently found in the cytoplasm than the interaction of these partners with DUX4. For DUX4c/FUS, the interaction signal was stronger at a tip of few myoblasts (* in S3B). In control/healthy myoblasts, SFPQ was mostly detected in the nucleoplasm, excluding the nucleoli, but also in the nuclei with a major presence at the periphery, in a ring around the nuclei (* in Fig 4) or in the cytoplasm (circle). A phosphorylated form of SFPQ had previously been reported in the cytoplasm [48]. Interestingly, DUX4c also presents such different types of localization (Figs 4 and 5, S5 Fig, see also Fig 6 for endogenous DUX4c). DUX4 exhibited preferentially diffuse nuclear staining (Figs 4 and 5) but was reported as less diffuse than DUX1, suggesting that DUX4 had more interactions sites in the nucleus [28]. In DUX4c-overexpressing myoblasts, SFPQ localization generally changed and was mostly detected in a few nuclear spots (arrows in Fig 4A). This localization was also observed following DUX4 overexpression but only in few myoblasts (arrows in Fig 4A).

### Endogenous DUX4c localizes to the cytoplasm during muscle differentiation

Because these different putative partners play important roles during muscle differentiation, we analyzed DUX4c localization during this process in healthy muscle cells. Almost all the nuclei of proliferating cells showed DUX4c immunofluorescence, except for nuclei with intense DAPI staining (the stars in Fig 6). DUX4c staining was generally localized at the periphery of the nuclei in distinct areas (6a), with no apparent change one day after the switch to differentiation medium. However, DUX4c staining decreased at day 3 (except for some nuclei), and progressive cytoplasmic labeling appeared in elongated myotubes (6b) with occasional spots of higher intensity (6c). The higher intensity of nuclear staining was mainly detected in myotubes harboring a cluster of nuclei (typically observed just after fusion) or in cells that were about to fuse. At day 6, no DUX4c nuclear staining was detected, and cytoplasmic staining was evident, with more intense areas either in the proximity of clusters of nuclei (e.g., 6d) or in non-fused myoblasts (e.g., the circled area). DUX4c staining was often observed close to one or several nuclei in clusters and on one side of the cell. Myotube tips also occasionally exhibited stronger DUX4c staining (e.g., the arrowhead), and the DUX4c staining partially co-localized with desmin in discrete dots (S6 Fig). Poly-A-binding protein 4 (PABPC4, also named PABP4), which is a perinuclear protein and a putative DUX4 partner, was also found in the myotube tips (S7A Fig)

During FSHD myoblast differentiation, DUX4c exhibited similar immunofluorescence patterns as in healthy myoblasts except that variable nuclear intensities were observed and some myoblasts with small nuclei already exhibited a diffuse cytoplasmic staining pattern (S7B Fig). During differentiation, DUX4c nuclear staining decreased, and cytoplasmic DUX4c staining was detected in some myoblasts (circles in S7B Fig) and myotubes. The nuclear and cytoplasmic DUX4c staining was stronger in abnormal myotubes with large clusters of nuclei; these myotubes were described as ‘disorganized’ by [34].

**Fig 7:**
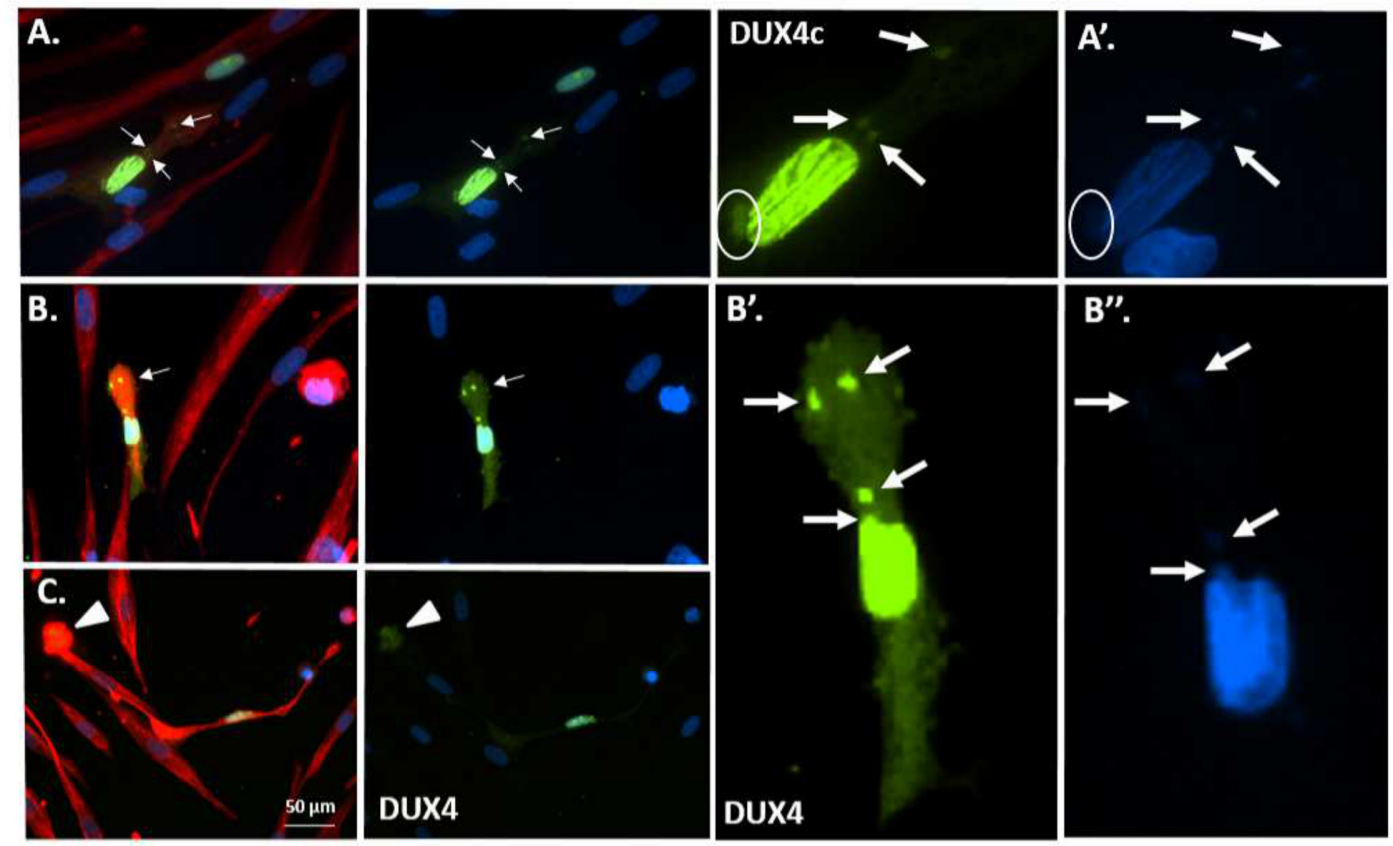
Partial co-localization of DUX4c or DUX4 and desmin in elongating transfected myoblasts. Healthy immortalized myoblasts were transfected with *pCIneo-DUX4c* (**A-C**) or -*DUX4* (**D-I**) expression vectors and switched to the differentiation medium. DUX4c, DUX4 (green), and desmin (red) were immunodetected at day 6, and myoblast nuclei were stained with DAPI. In addition to the strong DUX4/4c nuclear staining, elongating myoblast/myotubes had a few cytoplasmic spots (arrows) with similar or lower intensities; some of these spots were grouped in a myotube tip (**G-I**). The DUX4c nuclear staining (**A**) presented a pattern of linear stripes that may reflect interactions with the cytoskeleton and a nuclear bud (circle) (higher magnification is shown in the right panel and in S9 Fig).

When we overexpressed DUX4 or DUX4c in muscle cells and induced differentiation, we observed intense nuclear immunofluorescence and a few cytoplasmic dots in elongating myoblasts (Fig 7A and 7B and S8 and S9 Figs) or myotubes (Fig 7C, S6 Fig), which also partially co-localized with intense desmin staining (Fig 7B and 7C). We also observed DUX4c-stained areas at the nuclear periphery in DUX4c-expressing myoblasts (S8 and S10 Figs) and a large nuclear bud (DAPI staining) that seemed to emerge from the nuclei and contained DUX4c labeling (circle in S8 Fig). Other DAPI-stained regions in the cytoplasm were also observed in this myoblast (circle), but only one co-localized with DUX4c (arrow in S8 Fig). In the same cultures, some nuclei also exhibited DUX4/4c nuclear labeling corresponding to regions that excluded DAPI staining, which could be nucleoli (S11 Fig and stars in S10 Fig).

### DUX4 and DUX4c interact with desmin *in situ*

Using confocal microscopy, we observed a partial co-localization between DUX4 and desmin in myoblasts by co-immunofluorescence. To confirm that these proteins were sufficiently close to interact, we used the *in situ* PLA in muscle cells transfected with *DUX4/4c*-expressing vectors (S12A–S12F Fig). In some cells, we detected strongly fluorescent red dots, indicating the proximity of the two proteins in the cytoplasm and particularly around the nucleus, confirming that DUX4 or DUX4c could interact with desmin. In *DUX4c*-expressing cells, clusters of nuclei were observed, and DUX4c-desmin interactions were detected around some of them (S12F Fig). Moreover, in differentiated healthy or FSHD non-transfected cells, similar dots were observed using antibodies against DUX4 or DUX4c, showing an interaction between endogenous DUX4/DUX4c proteins and desmin (S12G–S12I Fig).

### DUX4c partially co-localizes with desmin in DMD and FSHD regenerating fibers

**Fig 8:**
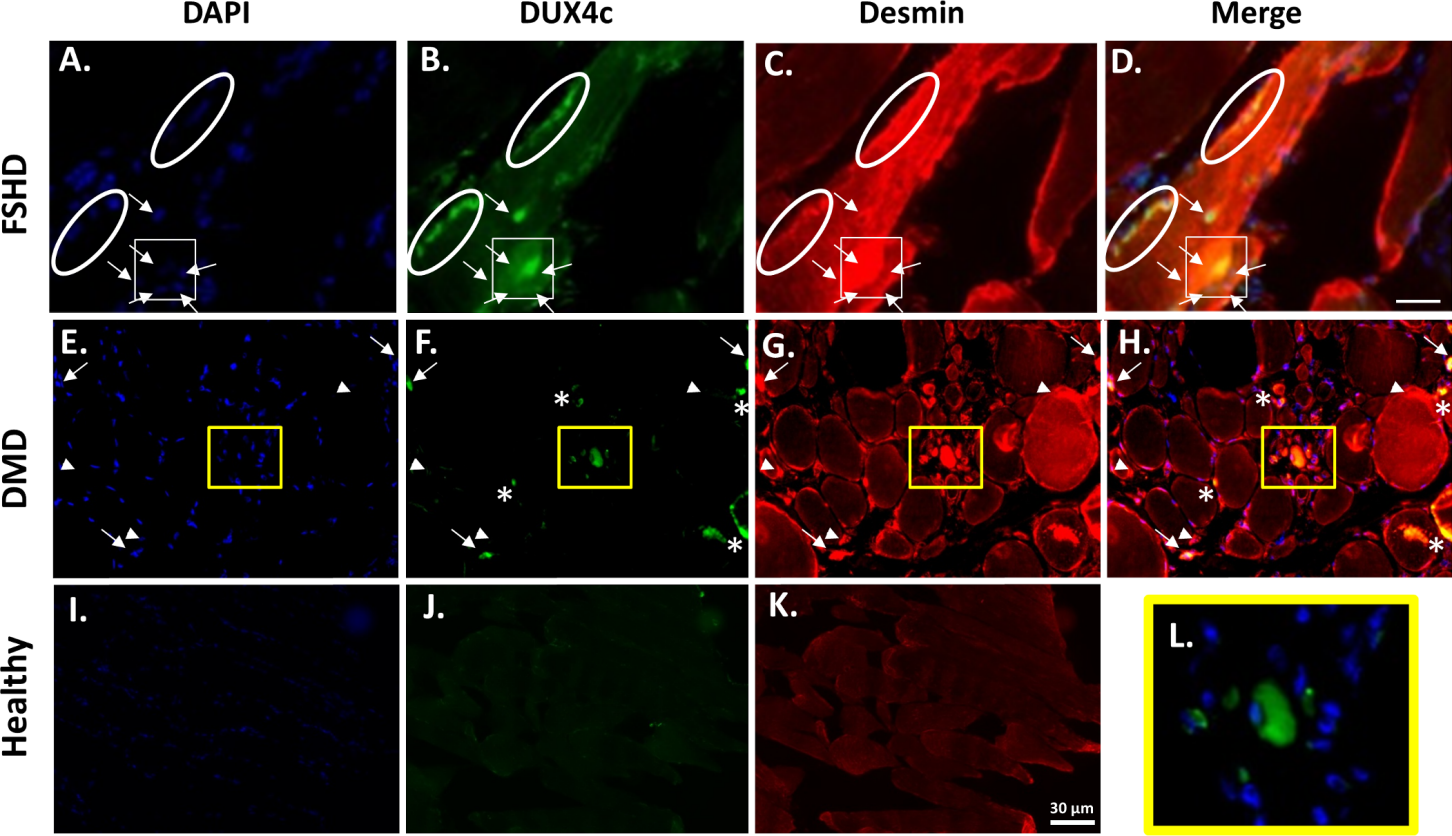
Endogenous DUX4c expression in FSHD and DMD muscle biopsies. DUX4c immunofluorescence (green) was detected in pathological (**A-H, L**) but not in healthy (**I-K**) muscle sections. In FSHD (**A-D**, longitudinal section) and DMD (**E-H, L**, transversal section), the arrows and circles show nuclei (DAPI, blue) harboring DUX4c staining or proximal to such staining. The circles highlight aligned nuclei in the FSHD fiber. Sarcoplasmic DUX4c staining (e.g., the boxed region) corresponds to the areas with intense desmin labeling (red) and partial co-localization (boxed region, arrows, circles, asterisks). The arrow heads indicate areas with intense desmin labeling without DUX4c co-staining. The boxed regenerating region in DMD with strong DUX4c cytoplasmic labeling is magnified in (**L**).

**Fig 9:**
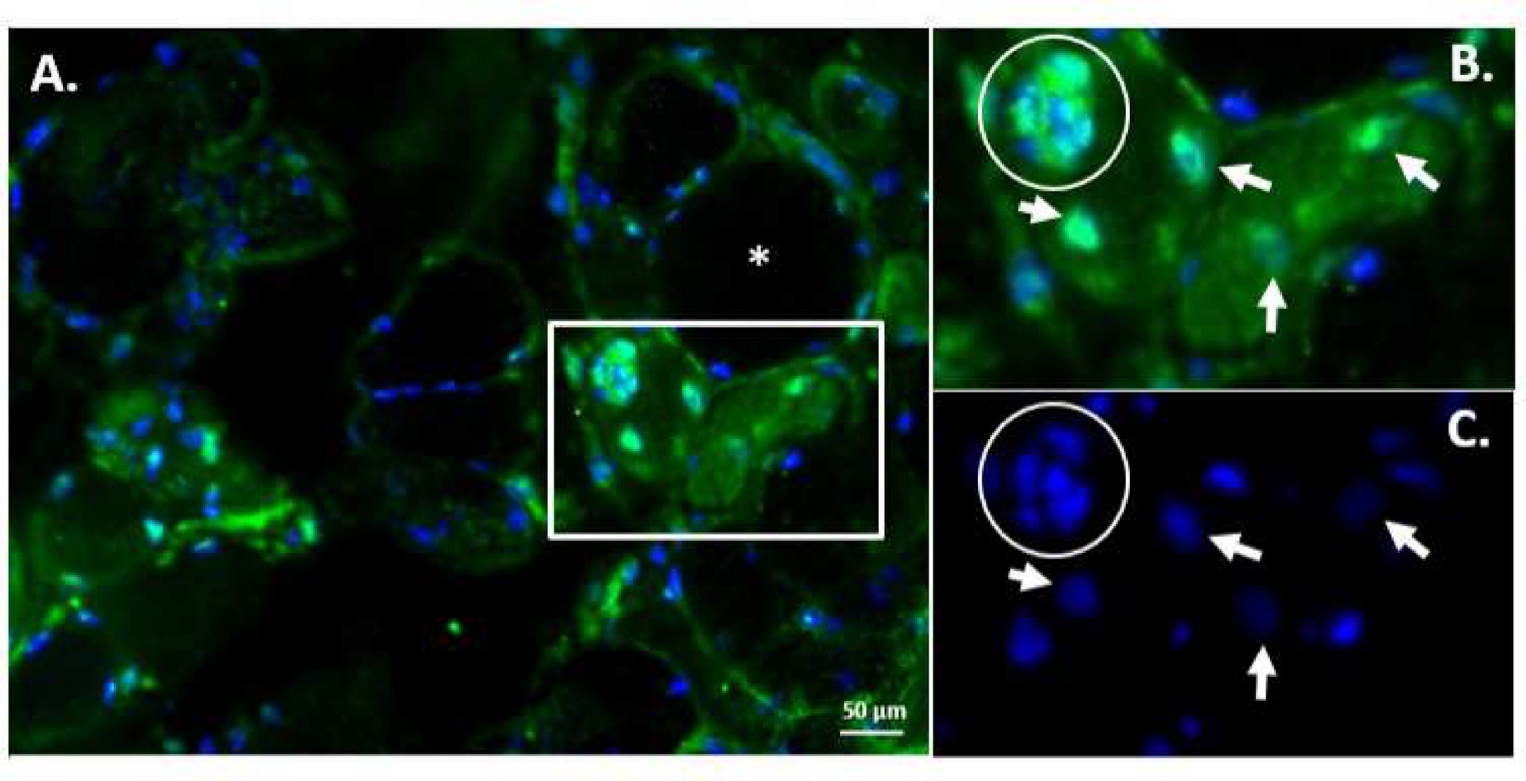
Endogenous DUX4c expression in a cluster of nuclei from pathological FSHD muscle. (**A**) Nuclear and sarcoplasmic DUX4c immunofluorescence (green) is observed in two adjacent fibers (boxed). The nuclei are stained with DAPI (blue). Most of the delocalized nuclei (in the cluster or not) in these two fiber sections show DUX4c staining. In the nuclei cluster (circle), DUX4c immunofluorescence is strong in the nuclei and in the sarcoplasmic space between them. A few other regions in the section also contain nuclear or cytoplasmic DUX4c staining. (**B-C**) Higher magnification of the boxed region.

We immunodetected DUX4c and desmin in muscle sections from healthy individuals or patients with DMD or FSHD. As expected, we did not observe any regeneration in healthy muscle. In contrast, DMD muscle sections presented intense desmin staining in muscle cells and fibers. This labeling was either localized at the fiber periphery, in a large spot inside the sarcoplasm or throughout the entire fiber section (Fig 8C and 8G). In the DMD and FSHD sections only, we detected intense DUX4c staining that always partially co-localized with intense desmin labeling (Fig 8A–8H: boxed region, arrows, stars). The reverse was not true; we could detect strong desmin staining in the absence of DUX4c labeling (arrow heads). In the DMD sections, DUX4c was mostly detected in the sarcoplasm and in localized areas (8F), as well as in some nuclei (arrows), as was previously observed in differentiating muscle cell cultures. We also found this localization in a few FSHD fibers (Fig 8I–8L). FSHD muscles are known to have a weak regeneration ability compared to DMD muscles. In FSHD sections, DUX4c also partially co-localized with intense desmin staining areas, either in the vicinity of dispersed nuclei (arrows) or around aligned nuclei (circles). Interestingly, we occasionally observed large clusters of nuclei in FSHD muscles harboring intense DUX4c labeling both in discrete areas within the nuclei and around them (Fig 9). In the same transversal myofiber as well as in an adjacent one, other large delocalized nuclei (arrowheads) were DUX4c positive and presented intense staining at some poles. These fibers also exhibited weak sarcoplasmic DUX4c staining. A negative staining control carried out in parallel using preimmune serum is depicted in S13 Fig.

## Discussion

### Cytoplasmic detection of DUX4c and DUX4 in differentiating myoblasts

During a time course study of immortalized myoblast differentiation, we surprisingly found that nuclear DUX4c staining progressively disappeared and was replaced by a cytoplasmic one, particularly around clusters of nuclei that are normally found for a limited time after fusion [49]. Myoblast fusion is an event that is rarely observed, which is consistent with the low number of muscle cells exhibiting high DUX4/4c cytoplasmic staining. We had previously missed the time of myoblast fusion and of DUX4c cytoplasmic labeling in the primary cultures (Ansseau 2009) that differentiated faster than immortalized ones. Recently, a differentiation time-course revealed heterogeneity among individual muscle cells within the same culture [50]. The DUX4c cytoplasmic location might also occur at different times in individual cells. In contrast, DUX4c was detected in approximately all of the myoblast nuclei, as was reported for MYF5 [51], which we had previously found to interact with DUX4c [19]. In differentiating myoblasts, Myf5 and MyoD can be observed as patches of cytoplasmic staining [51] similar to those described here for DUX4/4c (Figs 4 and 5 and S5 Fig). Indeed, DUX4 cytoplasmic staining was principally observed in differentiating myoblasts (Fig 5D–5I). The cytoplasmic labeling of DUX4 and DUX4c was observed with the use of two different antibodies (mouse MAb 9A12 and anti-DUX4c rabbit serum) and a microscope allowing for the detection of low abundance proteins (enhanced sensitivity and specificity).

Two different processes might cause DUX4/4c detection in the cytoplasm: either the proteins are retained in the cytoplasm after translation or they are normally transported to the nucleus followed by translocation to the cytoplasm. Consistent with the latter, we have identified and validated by GST pull-down and *in situ* PLA the IPO13 nuclear import protein as a DUX partner. IPO13 is known to interact with paired-type homeodomain (Pax) transcription factors that share high homeodomain sequence identity with DUX4/4c [4, 9, 27, 52]. Although DUX4 nuclear import was recently shown not to be mediated by importins [29], IPO13 also exhibits a nucleus-to-cytosol translocation that is involved in developmental and differentiation processes [53, 54]. Moreover, exportin 2 and RAN were also found as putative DUX4 partners. Homeoprotein nucleocytoplasmic shuttling depends on translational modifications, such as phosphorylation (reviewed in [55]). Several kinases and phosphatases were found among the putative DUX partners, and their roles in nuclear translocation should be investigated.

We have confirmed the cytoplasmic localization of DUX4c (partially co-localized with desmin) in a subset of regenerating fibers in DMD and FSHD muscle sections, in contrast to healthy muscles, which did not exhibit this labeling pattern. In contrast to DUX4, DUX4c was present at low levels in a majority of healthy myoblasts. Its sarcoplasmic localization in regenerating fibers supports a role in muscle regeneration as previously suggested in cell cultures [19, 56].

### DUX4/4c interact with desmin in elongating muscle cells

Using complementary approaches (Y2H screenings, myoblast protein co-purification and HEK293/myoblast GST pull-down), we have unexpectedly identified cytoplasmic proteins associated with the three types of cytoskeletal filaments (intermediate filaments/IF, actin filaments and microtubules) and myosin as binding partners of DUX transcription factors. These cytoskeletal partners were previously suggested to be artifacts because they are intrinsically “sticky” [57]. However, we validated the interaction with type III IF desmin by co-immunoprecipitation, GST pull-down, co-immunofluorescence and *in situ* PLA.

The functionality of this interaction was underscored by the cytoplasmic DUX4/4c detection during myoblast fusion, particularly observed at the tips of myotubes as they elongated and partially co-localized with desmin. There are many known (and unknown) changes during muscle differentiation, including increase in desmin abundance and post-translational modifications (PTM) [58]. Some of these modifications could be necessary to allow for interactions with DUX4/4c and could explain why only a part of the desmin positive staining was co-immunolabelled with DUX4c. A requirement for specific desmin PTM to allow for its interaction with DUX4/4c would be in accordance with the fact we could not find vimentin among DUX4/4c partners. Indeed, vimentin which is expressed in undifferentiated cells might not present similar PTMs. Alterations in desmin abundance or PTMs in rhabdomyosarcoma TE671 cells that have decreased its interaction with DUX4/4c have most probably allowed us to detect other protein partners on these more accessible targets. During myoblast elongation (before fusion), desmin is mostly present at the cell tips, as well as F-actin-enriched structures involved in actin remodeling and microtubule (MT) polymerization [59]. The production and remodeling of F-actin-enriched structures and MTs are key events during myoblast fusion [59–61] and sarcomere formation [59–64]. Several putative DUX4 partners can associate with microtubules and are also known to interact with actin (S3 Table: actin and tubulin-associated proteins). We also found different tubulins as putative DUX4/4c partners (GST pull-down and HaloTag co-purification), some of which were not found with the different negative controls used (S3 and S4 Tables). Moreover, DUX4/4c harbor a sequence related to the SxIP motif known to interact with tubulin [65] (Fig 1). We also found the motor protein dynein as a putative DUX4 partner. Dynein is suggested to mediate capture and tethering of microtubules at the cell cortex (inner face of the plasma membrane) and to enhance the stability of dynamic plus ends [66] where tubulin polymerizes in elongating muscle cells. The different subunits of the TCP1 chaperonin complex, which is involved in actin and tubulin folding [67], also co-purified with DUX4 or DUX4c (S3 and S4 Tables). Different actins were identified as putative DUX4/4c partners by each method employed in this study but they could be artifactual because they were also found with the negative controls (S4 Table). All these putative interactions should be further investigated.

**Fig 10:**
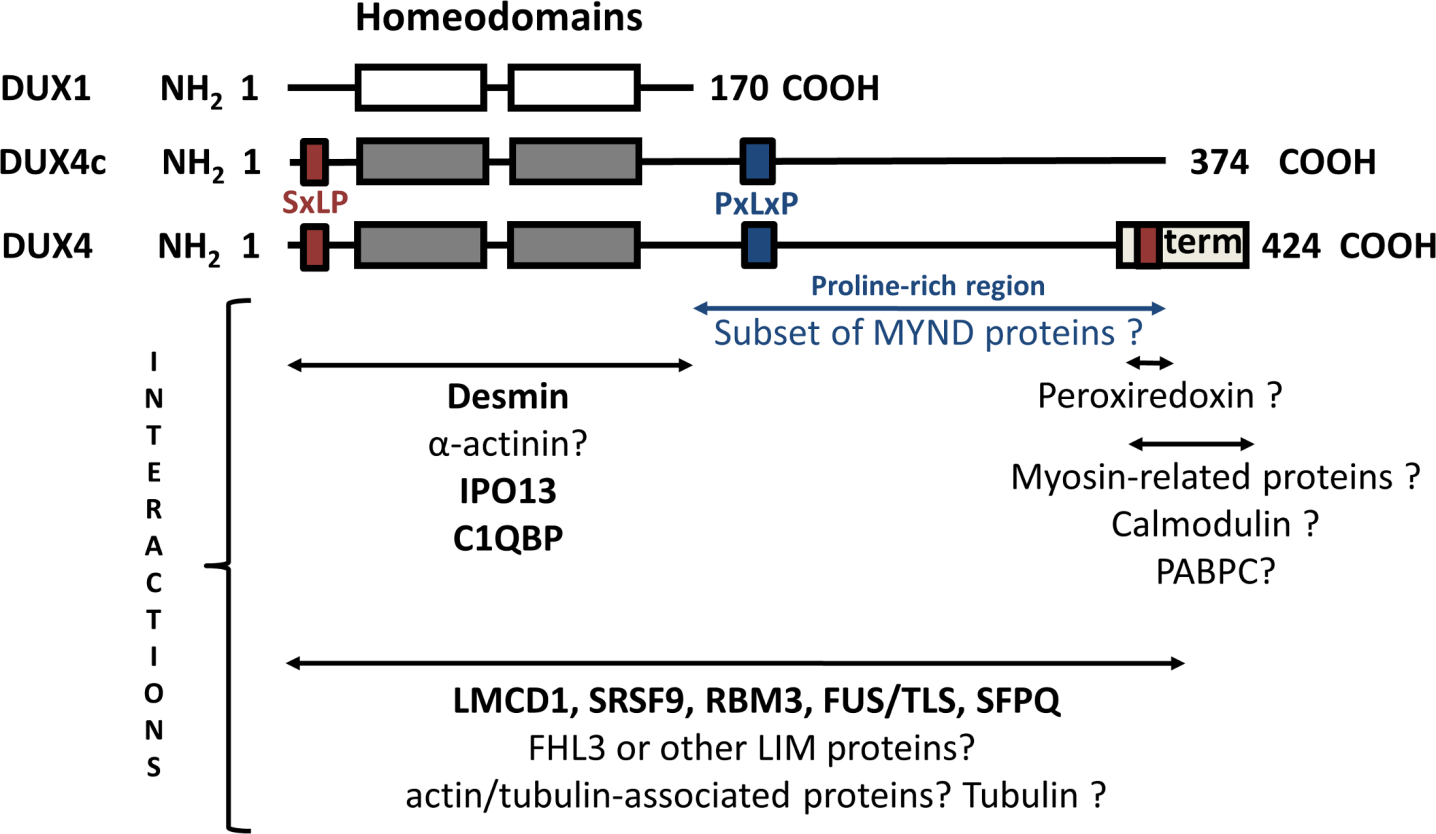
Domains involved in the validated and putative interactions with DUX binding partners. The different domains of the DUX proteins are indicated with their validated protein partners (bold): (i) the double homeodomain can interact with desmin, (ii) the DUX4 double homeodomain (identical to DUX4c) can interact with IPO13 and RNA-binding proteins C1QBP/Splicing factor 2 P32, and (iii) full-length DUX4 can interact with LMCD1, SRSF9, RBM3, FUS/TLS and SFPQ. Question marks indicate putative interactions: (i) the double homeodomain could interact with α-actinins, (ii) the PxLxP motif and proline-rich domain could interact with a subset of MYND proteins (e.g., SMYD1), (iii) the DUX4 terminal (term) region could interact with myosin-related proteins and calmodulin and (iv) the last 32 residues of DUX4c and the homologous DUX4 residues could interact with peroxiredoxin. The interactions with LIM proteins, actin- or tubulin-associated proteins and RNA-binding proteins are supported by Y2H or co-purification experiments and validated for the serine/arginine-rich splicing factor 9, the RNA binding Motif 3 proteins and the LIM and cysteine rich domain 1 protein (LMCD1).

DUX4, and particularly its carboxyl-terminal domain (Fig 10), interacted with different myosin proteins, most of which were non muscle-specific (NMM) and involved in premyofibril formation during differentiation [68, 69], including MYH9 (MYH II-A: identified by two independent co-purifications) and MYH10/NMMH II-B as DUX4 partners (S3 Table).

DUX4c gain-of-function induces cytoskeletal perturbations in muscle cell cultures with troponin T and α-tubulin delocalization (Vanderplanck et al, in preparation). Troponin T and α-tubulin delocalization is also observed in disorganized FSHD myotubes that contain increased relative abundances of cytoskeletal proteins involved in the regulation of the microtubule network organization and of myofibrillar remodeling [70]. The transcriptional target genes of DUX4 (the FSHD causal gene) and of DUX4c (also increased in FSHD muscles) identified to date cannot explain the cytoplasmic alterations observed in FSHD muscle sections, which show a higher number of splitting and branching myofibril bundles, as well as myofibril loss and sarcomere dysfunction [71–73] (Lancelot et al. in preparation).

Several putative partners identified in the Y2H screens contain zinc finger domains of the MYND or LIM type. We validated interaction of DUX4 with the LIM and cysteine-rich domain 1 protein (LMCD1) that is known to play roles in cardiac muscle hypertrophy by targeting NFAT (Nuclear Factor Of Activated T-Cells) [74, 75]. Similar to desmin, and, several other putative DUX4/4c partners (see S1 Text), LMCD1 is a Z-disc LIM-associated protein [75]. The skeletal and cardiac muscle-restricted protein SmyD1 interacts via its MYND domain with skNAC, a NACA isoform. This interaction is mediated by a PxLxP motif and the proline-rich skNAC domain [76–78]. Such domains are present in the carboxyl-terminal region of both DUX4c and DUX4 (but not in other DUX proteins that contain only the double homeodomain, such as DUX1) (Fig 1). Consistent with this finding, we did not identify such zinc finger proteins as putative DUX1 partners. During differentiation, SmyD1 and skNAC are translocated from the nucleus to the cytoplasm, where they play key roles by facilitating the folding and assembly of highly abundant myofibrillar proteins [79, 80]. In this process, actin polymerization modulates the localization of the NFAT transcription factor to the nucleus [81]. Both ILF2 and ILF3 subunits forming a NFAT complex [82], were identified as putative DUX4 partners in two independent experiments. Moreover, these subunits were associated with IGF2BP1-dependent mRNP-granules complex containing untranslated mRNAs [83]. We also found IGF2BP1 as a putative DUX4 partner (HaloTag co-purification and GST pull-down). Additional putative DUX4 partners included IGFBP3, known to heterodimerize with IGF2BP1, and 9 other IGF2BP1-interacting proteins including FUS (one of the validated partners, see below and ^&^-indicated partners in S3 and S4 Tables). IGF2BP1 associates with microtubules and polysomes; in the nucleus, it co-transcriptionally associates with actin (*ACTB*) mRNA before its export to the cytoplasm and its transport along the cytoskeleton to the cell membrane. At the cell membrane, IGF2BP1 is phosphorylated and releases the mRNA, allowing for the assembly of ribosomal 40S and 60S subunits and ACTB protein synthesis. Monomeric ACTB then assembles into the actin cytoskeleton [84].

Accumulating data have demonstrated that many proteins are multifunctional (e.g., [85]). Cytosolic DUX partners have additional functions in the nucleus. For example, desmin was reported to interact with lamins and single-stranded DNA/RNA and has been suggested to form a heterodimeric gene regulation complex with MyoD (reviewed by [86]). Additionally, FHL3, an actin-binding protein (see S1 Text), interacts with and inhibits the activity of MyoD bound to the *CKM* and *MYOG* promoters [87], while skNAC activates the *Myog* promoter [88]. In keeping with these observations, the partners we have identified and validated suggest that besides transcription DUX4/4c might have other roles in the cytoplasm that should be further investigated.

### DUX4/4c interacts with RNA-binding proteins

We identified and validated several RNA-binding proteins as DUX4 partners (see Tables 2 and S3), including splicing-associated factors such as C1QBP (also known as Splicing factor 2 P32), serine and arginine-rich splicing factor SRSF9 (also known as SRp30c), RNA-Binding Motif (RBM) 3, FUS/TLS and proline and glutamine rich splicing factor (SFPQ) also known as PSF (PTB-associated splicing factor). PTB (polypyrimidine track protein) regulates specific exon splicing in mRNAs during myogenesis [89]. C1QBP was found in each independent experiments to identify DUX4/4c partners (total: 10, see Tables 2 and 3, S3 and S4 Tables). C1QBP was also found as a DUX4c partner and interacted with DUX4/4c double homeodomains (Figs 3A and 10). In accordance we do not found C1QBP peptides in HaloTag-DUX4term co-IP (S3 Table). Another RBM protein (RBM24) is a putative DUX4c partner (Y2H). Little is known about the functions of the majority of RBM proteins; however, important developmental roles are suggested [90], such as a role for RBM3 in craniofacial development [91]. RBM3 (found in mES cells) is also involved in cell proliferation by regulating translation and has a reduced expression in terminally differentiated cells [92]. RBM24 (found in mature skeletal muscle) is known to regulate myogenic differentiation [93] and *MYOG* [94] and *p21* mRNA stability [95] and to be required for sarcomere assembly [96] and for normal somitogenesis [90]. Other serine and arginine-rich splicing factors (SRSF5/SRp40 and SRSF3/SRp20) were also found as putative DUX4 partners. C1QBP has been reported to bind SRSF9 and has been suggested to be involved in RNA-protein shuttling from the nucleus to the cytoplasm [44]. C1QBP is also involved in transcriptional regulation, ribosome biogenesis and mitochondrial translation [97, 98].

FUS and SFPQ were always found in 6 independent experiments using HaloTag co-IP to identify DUX4/4c partners. FUS is a multifunctional protein component of the heterogeneous nuclear ribonucleoprotein (hnRNP) complex that is involved in pre-mRNA splicing and the export of fully processed mRNA to the cytoplasm. In the cytoplasm, FUS associates with RNP granules that contain non-translating mRNAs [99]. FUS is also involved in transcription, pri-miRNA maturation and mRNA translation [100]. SFPQ is involved in several nuclear processes, such as transcription and pre-mRNA splicing in association with NONO, which is also a putative DUX4 partner (S3 Table) [101]. SFPQ/NONO have also been reported in cytoplasmic RNP granules [102]. Cytoplasmic localization of SFPQ was reported after phosphorylation of C-terminal tyrosines [48]. In the present study, we also observed a SFPQ ring immunostaining around the nuclei, similar to the DUX4/4c staining pattern in a few myoblasts (see below and S5 Fig). Cytoplasmic interactions with FUS and SFPQ were more frequently observed for DUX4c than DUX4. The interactions of desmin with DUX4/4c were cytoplasmic but also found at the nuclear periphery (S12 Fig). DUX4 and DUX4c were observed at the nuclear periphery in myoblasts; however, the ring staining was more frequent for DUX4c and thinner (S5 Fig). Desmin filaments links adjacent sarcomeres at the Z-discs, connects them to sarcolemma proteins, interacts with mitochondria, and also contacts the nuclear lamina and contributes to positioning of the nuclei (reviewed by [86, 103]). Interestingly, myotube nuclei are positioned abnormally following DUX4c overexpression (Vanderplanck et al, in preparation).

Among the different helicases identified, DDX5 and DDX17 were identified as putative DUX4/4c partners by two independent co-purifications (also by Y2H for DDX5). These helicases are involved in a wide range of cellular processes, including transcription activation, pre-mRNA splicing, mRNA export, rRNA and microRNA (miRNA) processing, and ribosome biogenesis (reviewed in [104, 105]). DDX5/17 are also β-catenin co-activators in the nucleus [104] and can associate with RBM4 [106, 107], which promotes the expression of many muscle-specific mRNAs from individual genes by modulating alternative splicing [108]. During muscle differentiation, RBM4 transiently translocates to the cytoplasm, where it participates in translation control [108]. DDX5/17 initiate and maintain specific mRNA splicing programs, resulting in protein forms that are involved in actin cytoskeletal dynamics [107], and help to activate MYOD1 transcriptional activity [89].

Several RNA-binding proteins identified as DUX4/4c partners are involved in the export of spliced mRNAs associated with cytoplasmic RNA granules. These granules are transported by microtubules to reach their correct location for translation (see above) [109]. We often observed that DUX4/4c cytoplasmic labeling co-localized with DAPI staining spots or buds on the nuclei that may correspond to such granules (Fig 7 and S8 and S9 Figs). Recently, the group of Vivian Budnick has discovered that muscle cells export RNP granules by nuclear envelope budding [110]. This mechanism is dependent on Lamin C, another putative DUX4 partner. We also identified other RNA-binding proteins, such as ribosomal proteins, translation factors and co-activators, as putative DUX4/4c partners. The mRNP granules contain numerous translation initiation factors, including small ribosomal subunits and are thought to contribute to translation regulation [111].

Moreover, we occasionally observed DUX4/4c localization in regions in a few differentiating myoblasts that may correspond to nucleoli (S10 and S11 Figs), which has been described for several DUX4/4c binding partners (see S3 and S4 Tables) that regulate rRNA transcription and processing ([112]; Fig 6, S5 Fig). During myoblast fusion, large amounts of RNA and RNPs may translocate from the nucleoli to the cytoplasm [113] and the 3 types of cytoskeletal filaments (IF, MTs and actin microfilaments) are involved in their transport to synthesize active myofibril proteins [114]. We showed that during this process endogenous DUX4c (in healthy and FSHD cells) and DUX4 (in FSHD cells) accumulate in the cytoplasm in association with desmin. Moreover, DUX4c and DUX4 are associated with the RNA-binding proteins FUS and SFPQ in the nucleus and in the cytoplasm and with cytoplasmic nucleic acids (DAPI staining).

In summary, our four independent experiments to identify DUX partners identified mRNA-binding and cytoplasmic proteins. A majority of the DUX4/4c partners found are known to have dual functions, one in the nucleus and the other one in the cytoplasm, particularly in differentiating muscle cells. Several act in common pathways, such as in the regulation of *Myogenin* expression via modulation of MYOD1 activity (RBM24, skNAC, FHL3, DDX5/17) and myoblast differentiation including fusion and myofibrillogenesis (as evidenced by desmin in elongating tips in parallel with actin/tubulin remodeling and folding, see above and S1 Text). Although these proteins are frequently considered experimental artifacts in partner identifications, these interactions could nevertheless be of functional relevance and should be further investigated. Moreover, the Interleukin Enhancer Binding Factor 2 and 3 (ILF2/3) are known to associate with IGF2BP1-dependent mRNP granules. These granules travel along microtubules to bring mRNA to the plasma membrane for translation. The validated partner FUS (hnRNP P2) as well as the putative ones DHX9 (an ATP-dependent RNA helicase A), ELAVL1, HNRNPU, HNRNPH1, PABPC1, PCBP2, PABPC4, SYNCRIP (hnRNP Q), nucleolin and several ribosomal proteins are present in these mRNP granules (Protein-protein interaction databases at UniProtKB; [115]). Other DUX protein partners are also part of known RNP complexes, such as C1QBP/SRSF9, SFPQ/NONO, DDX5/17, RBM3/RPL4. All the mRNP granules have features in common and are dynamic, self-assembling structures that harbor non-translating mRNAs bound by various proteins that regulate mRNA translation, localization, and turnover [111]. Further experiments will be needed to confirm whether DUX4/4c are part of these multifunctional complexes and involved in the regulation of mRNP granules.

DUX4 overexpression is toxic in muscle cells [9, 26, 27] and might cause aberrant cytoplasmic localization of nuclear factors in degenerating myotubes. Nevertheless, endogenous DUX4c (expressed in almost all myoblasts in contrast to DUX4) is detected in the cytoplasm during myoblast differentiation. Moreover, with different antibodies (mouse monoclonal and rabbit antisera) used in several independent experiments (this study and Vanderplanck et al, in preparation), we always observed cytoplasmic DUX4 and DUX4c at the tip of elongating myotubes or next to clusters of nuclei thus arguing against an artifactual localization.

### Proteins involved in muscle differentiation might be disturbed in FSHD by interactions with DUX4/4c

Until now, research has focused on the transcriptional regulation function of DUX4 to explain the physiopathology of FSHD [10, 24, 30, 31, 116]. The DUX4 protein is known to exhibit strong transcriptional activity via its carboxyl-terminal domain [6, 117]. DUX4c contains a shorter carboxyl-terminal domain than DUX4, and it has a weaker activity toward the *PITX1* and *CRYM* gene promoters [10, 41]. This different transcriptional activity was also confirmed by our one-hybrid experiment in yeast (data not shown). DUX4c and DUX4 contain identical homeodomains and may therefore bind to the same target genes. However, the precise DNA target may be influenced by interaction with specific partners, and this question should be addressed in ChIP-seq experiments.

If additional functions were defined for DUX4/4c in association with cytoplasmic protein partners described in this study, they might be linked to the occasional cytoskeletal abnormalities reported in FSHD muscles (see above) [37, 118, 119]. These might be related to the known sporadic DUX4 expression in FSHD muscle cells [37, 118, 119] escaping cell death in conditions where transient DUX4 expression burst is not fatal to the fiber or takes considerably longer to disrupt it [26]. Moreover, we observed DUX4c up-regulation in some FSHD muscles [19], which could be related to the normal regeneration process. However, in our previous study, higher amounts of DUX4c were generally detected in FSHD samples (6- to 15-fold increase compared to controls) containing few regenerating fibers in comparison to the DUX4c level found in highly regenerating DMD muscles (3-4-fold increase) [19]. The larger amount of DUX4c differs between patient muscles, and this difference may contribute to the pathology. Indeed, we also observed the abnormal localization of contractile-associated proteins and mis-localization of nuclei following DUX4c over-expression in muscle cells (Figs 7F, 9 and S4A and Vanderplanck et al in preparation). Moreover, we often observed abnormal intracellular localization of DUX4c compared to healthy muscle cells: FSHD myoblasts already harbor cytoplasmic DUX4c and, during late differentiation, FSHD myotubes still contained nuclear DUX4c (S6B Fig). This was also observed in FSHD muscle fiber sections (Fig 9). We also have preliminary data showing both nuclear and cytoplasmic endogenous DUX4 in FSHD myotubes containing nuclei clusters (after fusion). Interestingly, in the local myopathy model developed by injection of an AAV-DUX4-V5tag expression vector, this protein was specifically immunodetected both in the nuclei and the cytoplasm of degenerating myofibers [47]. These data need further investigation to determine whether abnormal localization of DUX4 or DUX4c plays a role in FSHD physiopathology.

Differentiation defects have been reported in FSHD muscles, such as the inhibition of MYOD1 target genes [120, 121] and genes involved in normal myogenesis, such as those encoding proteins related to muscle structure proteins and stress responses [122]. DUX4 may impact myogenesis at the transcriptional level [27, 30] and by interacting with proteins that regulate these genes, such as *MYOG* (see above), which is upregulated in FSHD myoblasts in comparison to healthy immortalized myoblasts [36]. The cytoskeleton plays critical roles in cellular structure, proliferation, and intra- and inter-cellular signaling. This is especially important in muscle cells, and the disruption of the cytoskeletal network can lead to a broad spectrum of myopathies such as desminopathies (reviewed in [123]). Cytoskeletal perturbations also impact mitochondrial localization and function. Abnormal mitochondrial morphologies and increased sensitivity to oxidative stress have been reported in FSHD muscles [72, 120, 124, 125]. Of note, we also found antioxidant enzymes, such as glutathione peroxidase and peroxiredoxins, among the putative DUX4/4c binding partners (S3 Table, Fig 10).

In desminopathies and other protein-aggregate diseases, a crosstalk has been suggested between protein folding and RNP aggregation [126]. For example, muscles from desminopathies contain cytoplasmic aggregates of the RNA-binding protein TDP-43, a component of stress granules [127]. TDP-43 shares several normal and pathological functions with FUS (a DUX4/4c partner), such as the formation of pathological cytoplasmic aggregates in degenerating motor neurons in ALS that are also associated with mitochondrial damage [128, 129]. Some validated and putative DUX4/4c RNA-binding partners are related to neuronal differentiation (S3 Table), and DUX4 overexpression in mES cells induces neurogenesis [25]. TDP-43 nuclear aggregates were recently shown to be induced by DUX4 expression in muscle cells and suggested as a potential pathological mechanisms in FSHD [33]. In our study, we observed that DUX4c overexpression in myoblast induced delocalization of nuclear SFPQ, which may alter the function of this splicing factor. SRp40, another DUX4 partner, is involved in the specific splicing of Troponin T mRNA that is altered in FSHD muscles, yielding to contraction defects [130]. In FSHD myoblasts, the splicing of muscle-specific mRNAs and mRNA stability are disrupted [131, 132] (reviewed in [133]). Some mRNP are also thought to function in mRNA decay [111], and DUX4 was recently reported to strongly inhibit NMD resulting in a global accumulation of mRNAs normally degraded as NMD substrates [32].

In conclusion, during healthy and pathological muscle differentiation we detected the DUX4 and DUX4c transcription factors in the nucleus but also in the cytoplasm in association with proteins such as desmin and the RNA-binding proteins FUS and SFPQ. Other RNA-binding proteins playing roles during muscle differentiation were also validated as DUX4/4c partners. Additional studies should define the molecular mechanisms of these interactions in muscle differentiation and in neuro-muscular pathologies. Moreover, because a large portion of the DUX4c protein is identical to DUX4, the current development of therapeutic approaches to inhibit DUX4 expression in FSHD should avoid interference with the normal function of DUX4c. Indeed, the inhibition of DUX4c decreases the myoblast proliferation required for regeneration (Vanderplanck et al in preparation).

## Supporting Information

S1 Fig Purification of HaloTag-DUX4 or -DUX4c protein complexes. TE671 cells were transfected with HaloTag-DUX4 or -DUX4c expression vectors. Cells were harvested 24 h later and lysed. The HaloTag protein complexes were then purified by affinity chromatography on Halo-Link resin and released by digestion with TEV protease as described in Material and Method. Twenty-five μg proteins of the purified HaloTag complex were analyzed by SDS PAGE followed by a silver staining to show the complexity of the protein extract and DUX4 (*) abundance. The DUX4c purification was not as efficient.
(TIFF)

S2 Fig GST pull-down assays of DUX proteins with desmin. DUX proteins or luciferase (negative control) were radiolabeled during *in vitro* transcription/translation (T/T) in the presence of [^35^S]-cysteine in a reticulocyte lysate. GST-desmin or GST alone (black and white arrowheads, respectively) expressed in *E*. *coli* were coupled with Glutathione Sepharose beads and incubated with either the indicated radiolabeled proteins (T/T), [^35^S]-cysteine alone or buffer alone. After centrifugation, the T/T products and GST pull-down products were analyzed by SDS-PAGE followed by Coomassie blue staining (A) or by autoradiography (B). The arrows show DUX1 and DUX4 (but not DUX4-t) interaction with GST-desmin but not with GST alone (Luc: luciferase, DUX4-t: DUX4 tail).
(TIFF)

S3 Fig DUX4 and DUX4c interaction with IPO13 and C1QBP. (A) GST pull-down samples of GST-DUX4, GST-DUX4c, GST-B56α (unrelated protein) or GST alone incubated with radiolabeled IPO13 (following in vitro T/T as in **S2 Fig**) were analyzed by SDS-PAGE followed by autoradiography. (B) *In situ* Proximal Ligation Assay (PLA) performed using antibodies against DUX4 (9A12 mAb) and IPO13 in FSHD myoblasts shows a DUX4/IPO13 interaction in a few cells, with several PLA spots at the periphery of the nuclei that were stained with DAPI (blue). (C) HEK293 cells were transfected or not (untransfected) with plasmids expressing V5 epitope-tagged DUX4 (DUX4.V5) or a DUX4 homeodomain mutant defective in DNA binding (HOX1.V5). Cell protein extracts before (input) or after immunoprecipitation with anti-V5 antibodies (V5 Co-IP) were analyzed by SDS-PAGE, transferred to a western blot and immunoblotted with anti C1QBP antibodies.
(TIFF)

S4 Fig DUX4 and DUX4c interaction with splicing factors SFPQ and FUS. *In situ* Proximal Ligation Assay (PLA) using antibodies against DUX4 or DUX4c and SFPQ (A) or FUS (B) was performed in healthy myoblasts transfected with a strong DUX4- or DUX4c-expression vector (p*CIneo-DUX4* or *-DUX4c*), the empty parental vector (p*CIneo*) or a vector with the endogenous promoter (*pENTR-DUX4c*) as indicated. More interaction spots (red) were detected at or near the nuclear periphery or in the cytoplasm in cells expressing DUX4 or DUX4c. The star (*) points to a high PLA spot density showing DUX4c-FUS interactions at the tip of a myoblast. Scale bar: 10 μm.
(TIFF)

S5 Fig DUX4 and DUX4c immunodetection at the nuclear periphery. TE671 cells were transfected with the *pCIneo-DUX4* (top panel) or -*DUX4c* (bottom panel) expression vectors. Confocal microscopy analyses were performed on cells immunostained with rabbit anti-DUX4 serum (#314, top left panel) or anti-DUX4c (bottom left panel) or mouse monoclonal anti-DUX4 (9A12, right panels). The nuclei were stained with DAPI (blue). Arrowheads and circles indicate cytoplasmic DAPI staining; arrows and circles indicate DUX4/4c cytoplasmic staining. Magnifications of the circled regions from the top panels are shown in the middle panels (left and right). The yellow box shows nuclear DUX4 staining in regions with low DAPI staining (magnified in the central panel).
(TIFF)

S6 Fig Partial co-localization of endogenous DUX4c and desmin in myotube tips. DUX4c (rabbit serum, red) and desmin (mouse monoclonal, green) were detected in an immortalized myoblast line by immunofluorescence. Desmin was concentrated at the tips of an early myotube after 1 day of differentiation (A). This myotube exhibited nuclear as well as cytoplasmic DUX4c staining (B; D). The nuclei were stained with DAPI (C). The accumulation of DUX4c spots was denser in the elongating myotube tips and partially co-localized with desmin (A). Two arrows point to intense DUX4c spots in the boxed myotube tip that was enlarged in (A’,B’,D’). Merged pictures are shown (D,D’). Scale bar: 50 μm.
(TIFF)

S7 Fig (A) PABPC4 (a putative DUX4/DUX4c partner) expression in elongating myotubes. PABPC4 (red) and desmin (green) were stained by immunofluorescence in healthy primary myotubes after 4 days in the differentiation medium. PABPC4 was detected in elongating myotubes around the aligned nuclei but also close to a tip, where it partially co-localized with desmin. Other desmin-positive cells were not labeled for PABPC4. The nuclei were stained with DAPI. (B) Endogenous DUX4c in differentiating FSHD myoblasts. DUX4c was immunodetected in proliferating immortalized myoblasts and during a differentiation time-course. Nuclear staining was observed in almost all nuclei in myoblasts and after one day in the differentiation medium, as in healthy cells but with variable intensities; the more intense nuclear signals are observed in myoblasts showing weak cytoplasmic staining and small nuclei (arrows). Higher cytoplasmic labeling on one side of a cell was also observed in the proliferation medium (circle). During differentiation, DUX4c was progressively detected in the cytoplasm, and the nuclear labeling decreased at day 3. DUX4c nuclear staining was generally lost at day 6, and some myotubes or myoblasts (circles) presented strong cytoplasmic staining. A cluster with a high number of nuclei (boxed) had strong DUX4c labeling in and around the nuclei. This is similar to the DUX4c immunostaining that was observed in FSHD muscle biopsies (**Figs 8** and **9**) and to the cluster observed in **S8 Fig** (desmin-DUX4c interaction in DUX4c-overexpressing cells).
(TIFF)

S8 Fig Localization of DUX4c at the nuclear periphery and in a nuclear bud. Healthy immortalized myoblasts were transfected with a DUX4c expression vector, and detection of DUX4c (red) and Alpha-tubulin (green) by immunofluorescence was carried out after 6 days in the differentiation medium (as in **Fig 7**). The nuclei were stained with DAPI. DUX4c was observed at the nuclear periphery (as in S5 Fig). The circle surrounds a nuclear bud containing DUX4c and DAPI staining as well as cytoplasmic DUX4c that co-localized with DAPI staining (indicates by the arrow).
(TIFF)

S9 Fig Nuclear and cytoplasmic localization of DUX4c in an elongating transfected myoblasts. Higher magnification of **Fig 7**. DUX4c (green) and desmin (red) were detected by immunofluorescence at day 6, and myoblast nuclei were stained with DAPI. Nuclear budding could be observed (circle), and a few cytoplasmic spots (arrows) were stained for DUX4c. The DUX4c nuclear staining presented a pattern of linear stripes that might reflect interactions with the cytoskeleton above the nucleus.
(TIFF)

S10 Fig Nuclear and cytoplasmic DUX4c detection in transfected muscle cells. Healthy immortalized myoblasts were transfected with a DUX4c expression vector, and immunodetection was carried out after 6 days in the differentiation medium (as in **Fig 7**). (Top) A myoblast (boxed) shows nuclear and weak cytoplasmic DUX4c labeling (green). Clusters of nuclei in myotubes (desmin in red) also exhibit a stronger DUX4c staining in regions unstained for DAPI that could be nucleoli (as in S7 Fig*). (Bottom) Magnification of the boxed myoblast showing DUX4c labeling at a lower exposure time in distinct areas of the nuclei, several of them located at the nuclear periphery.
(TIFF)

S11 Fig Nuclear localization of DUX4/DUX4c in regions unstained for DAPI and in nuclear buds. Healthy immortalized myoblasts were transfected with DUX4c (A) or DUX4 (B) expression vectors, and immunofluorescence detection was carried out after 6 days in the differentiation medium (as in **Fig 7**). In a few nuclei, stronger DUX4 and DUX4c staining was observed in regions unstained for DAPI (arrowheads) that could be nucleoli. Middle panels: magnification of the boxed nucleus (left: DUX4c and DAPI staining) and the circled nuclear bud with strong DUX4 staining (right: DAPI and DUX4 staining).
(TIFF)

S12 Fig Desmin interaction with DUX4 and DUX4c shown by proximity ligation assay. (A-F) Healthy immortalized myoblasts were transfected with vectors expressing DUX4 (A-B) or DUX4c (C-F) under control of the *CMV* promoter (C-D) or their endogenous promoter (*pDUX*: A-B, E-F) and fixed either 24 h post transfection (A) or after 4 days in differentiation medium (B-F). (G-J) Untransfected immortalized healthy (G-I) or FSHD (J) myoblasts expressing endogenous DUX4c were differentiated and fixed 4 days later. *In situ* PLA was performed using the 9A12 mouse MAb to detect DUX4/DUX4c (A-C, F,J) or anti-DUX4c rabbit serum (D-E, G-I) and rabbit or mouse antibodies against desmin, respectively (A-J). The red spots indicate a desmin/DUX interaction, and the nuclei were stained with DAPI. The arrows point to high spot densities close to nuclei.
(TIFF)

S13 Fig Negative controls in FSHD muscle biopsies. A rabbit preimmune serum was used in place of the anti-DUX4c serum. (**A**) Longitudinal section adjacent to the one used in **Fig 7A–7D**. (**B**) Transverse section adjacent to the one used in **Fig 9**.
(TIFF)

S1 Table (PDF)

S2 Table (PDF)

S3 Table (PDF)

S4 Table (PDF)

S1 Text Supporting Information Text. (PDF)
