# Supplementary figures and images for: Homologous Transcription Factors DUX4 and DUX4c Associate with Cytoplasmic Proteins during Muscle Differentiation
Source: PLoS One. 2016 Jan 27;11(1):e0146893. doi: 10.1371/journal.pone.0146893 (PMC4729438; doi:10.1371/journal.pone.0146893)

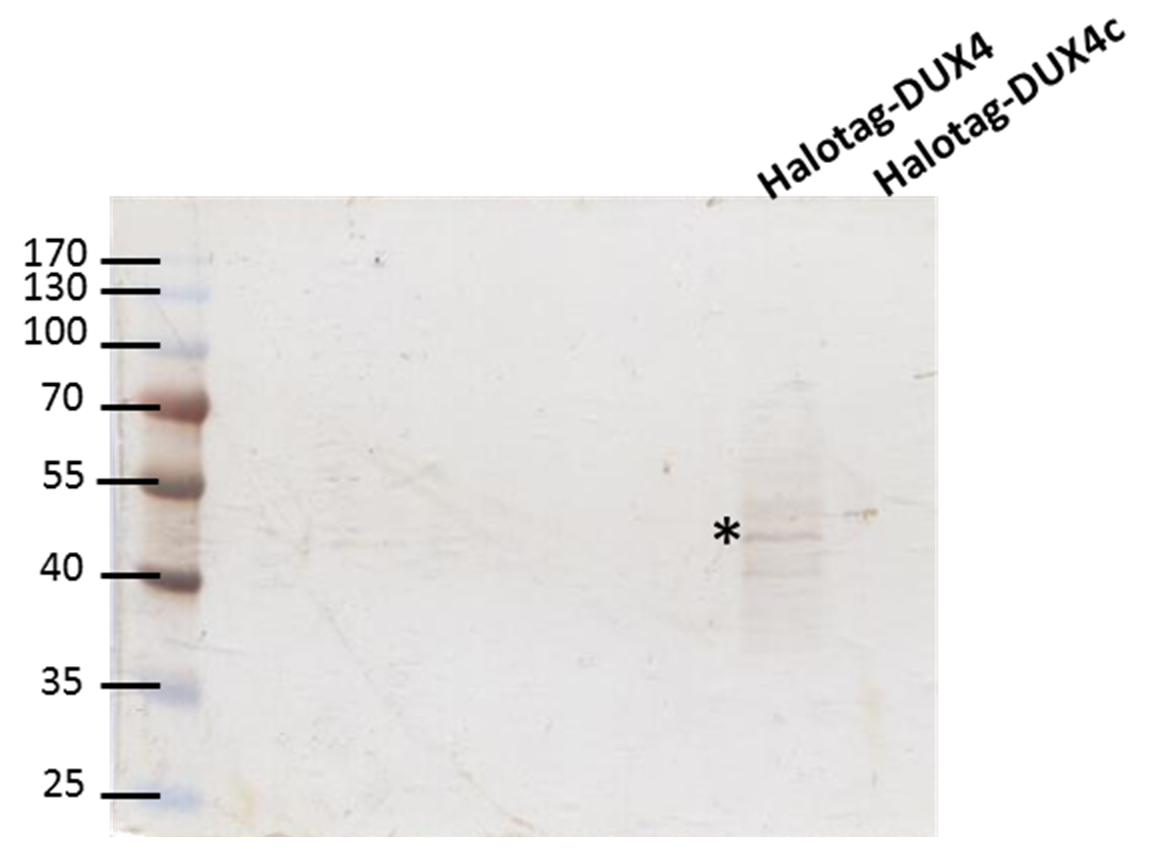

Supplement: S1 Fig — TE671 cells were transfected with HaloTag-DUX4 or -DUX4c expression vectors. Cells were harvested 24 h later and lysed. The HaloTag protein complexes were then purified by affinity chromatography on Halo-Link resin and released by digestion with TEV protease as described in Material and Method. Twenty-five μg proteins of the purified HaloTag complex were analyzed by SDS PAGE followed by a silver staining to show the complexity of the protein extract and DUX4 (*) abundance. The DUX4c purification was not as efficient. (TIFF) [file pone.0146893.s001.tiff]

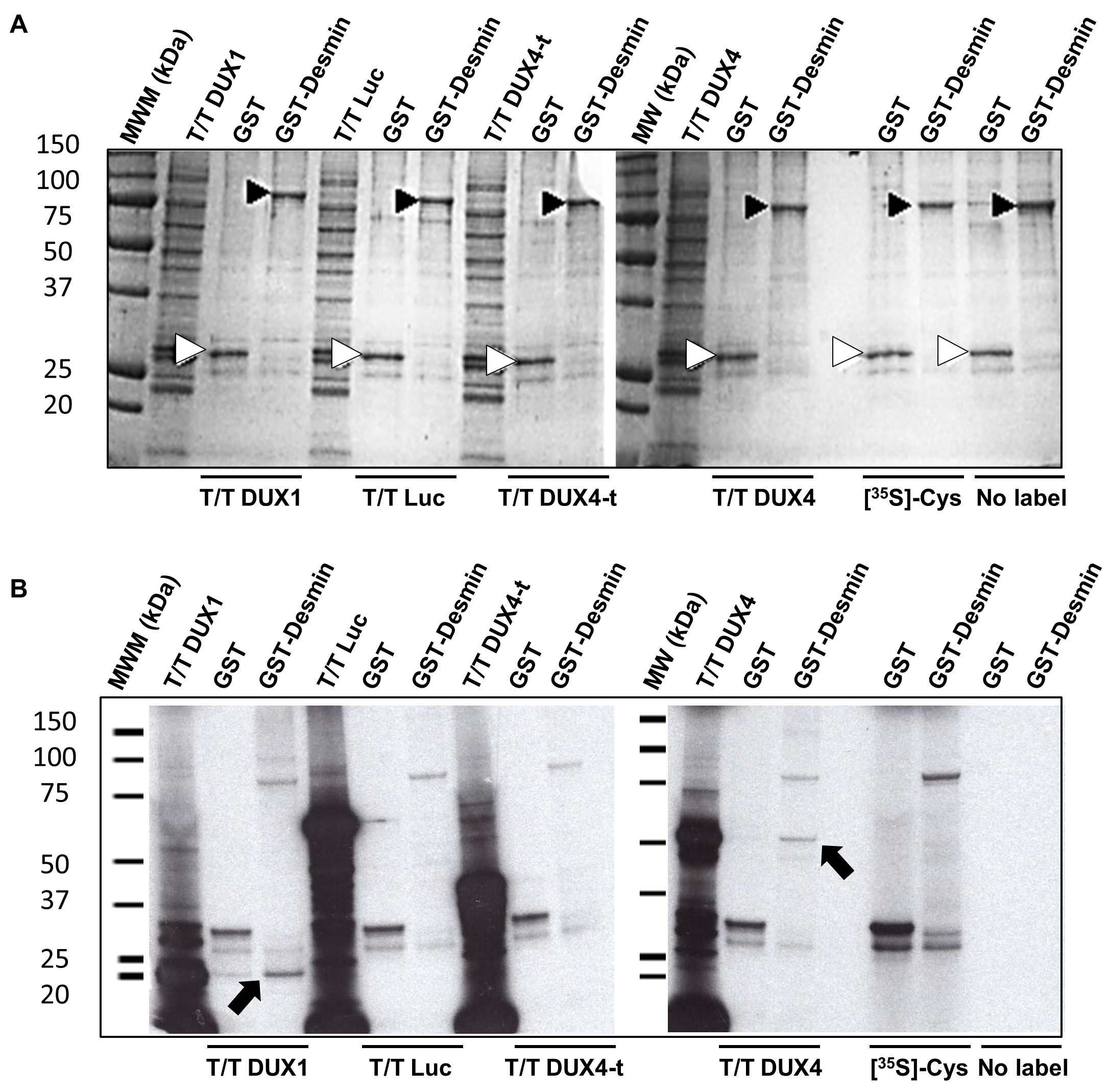

Supplement: S2 Fig — DUX proteins or luciferase (negative control) were radiolabeled during in vitro transcription/translation (T/T) in the presence of [35S]-cysteine in a reticulocyte lysate. GST-desmin or GST alone (black and white arrowheads, respectively) expressed in E. coli were coupled with Glutathione Sepharose beads and incubated with either the indicated radiolabeled proteins (T/T), [35S]-cysteine alone or buffer alone. After centrifugation, the T/T products and GST pull-down products were analyzed by SDS-PAGE followed by Coomassie blue staining (A) or by autoradiography (B). The arrows show DUX1 and DUX4 (but not DUX4-t) interaction with GST-desmin but not with GST alone (Luc: luciferase, DUX4-t: DUX4 tail). (TIFF) [file pone.0146893.s002.tiff]

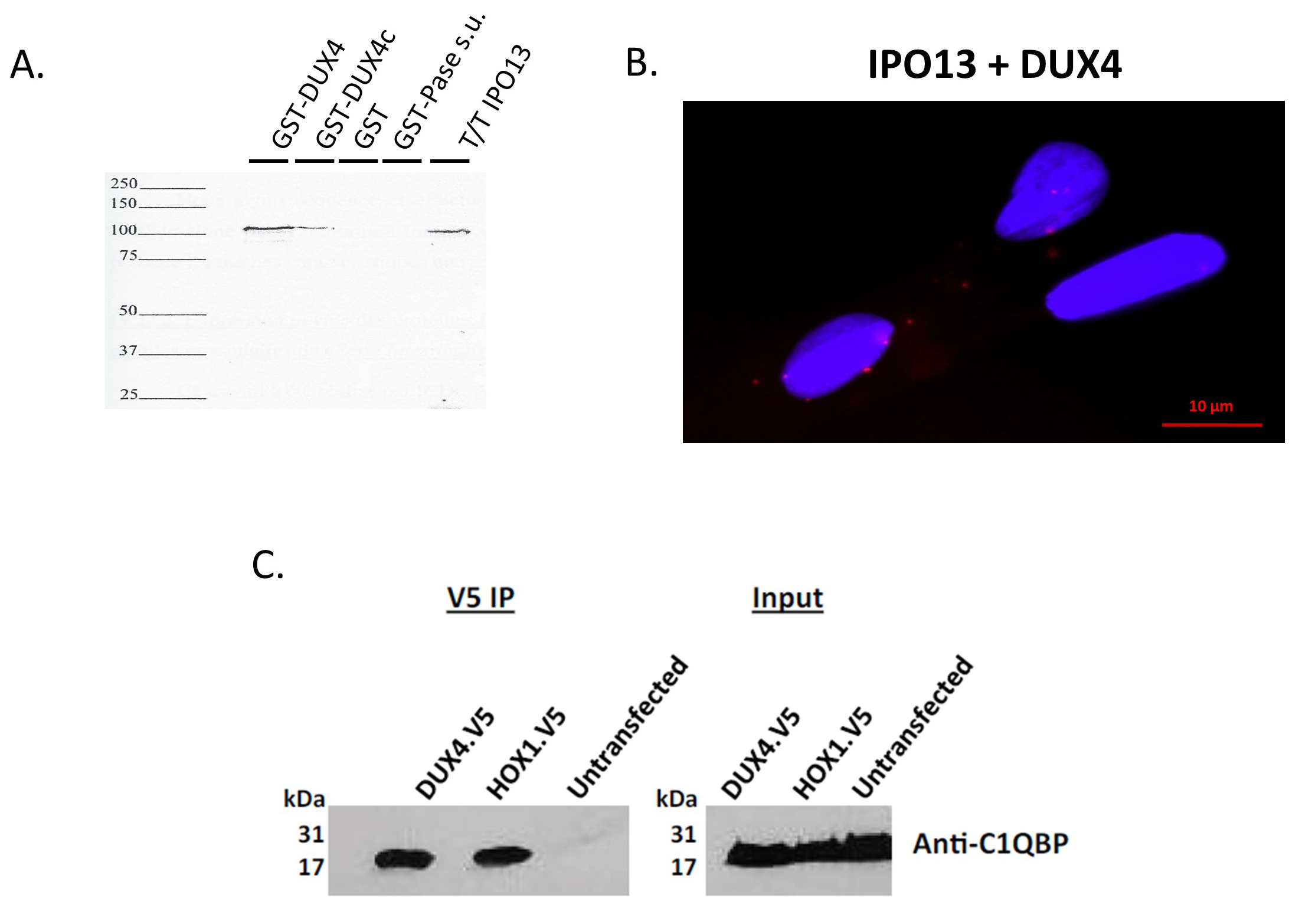

Supplement: S3 Fig — (A) GST pull-down samples of GST-DUX4, GST-DUX4c, GST-B56α (unrelated protein) or GST alone incubated with radiolabeled IPO13 (following in vitro T/T as in S2 Fig) were analyzed by SDS-PAGE followed by autoradiography. (B) In situ Proximal Ligation Assay (PLA) performed using antibodies against DUX4 (9A12 mAb) and IPO13 in FSHD myoblasts shows a DUX4/IPO13 interaction in a few cells, with several PLA spots at the periphery of the nuclei that were stained with DAPI (blue). (C) HEK293 cells were transfected or not (untransfected) with plasmids expressing V5 epitope-tagged DUX4 (DUX4.V5) or a DUX4 homeodomain mutant defective in DNA binding (HOX1.V5). Cell protein extracts before (input) or after immunoprecipitation with anti-V5 antibodies (V5 Co-IP) were analyzed by SDS-PAGE, transferred to a western blot and immunoblotted with anti C1QBP antibodies. (TIFF) [file pone.0146893.s003.tiff]

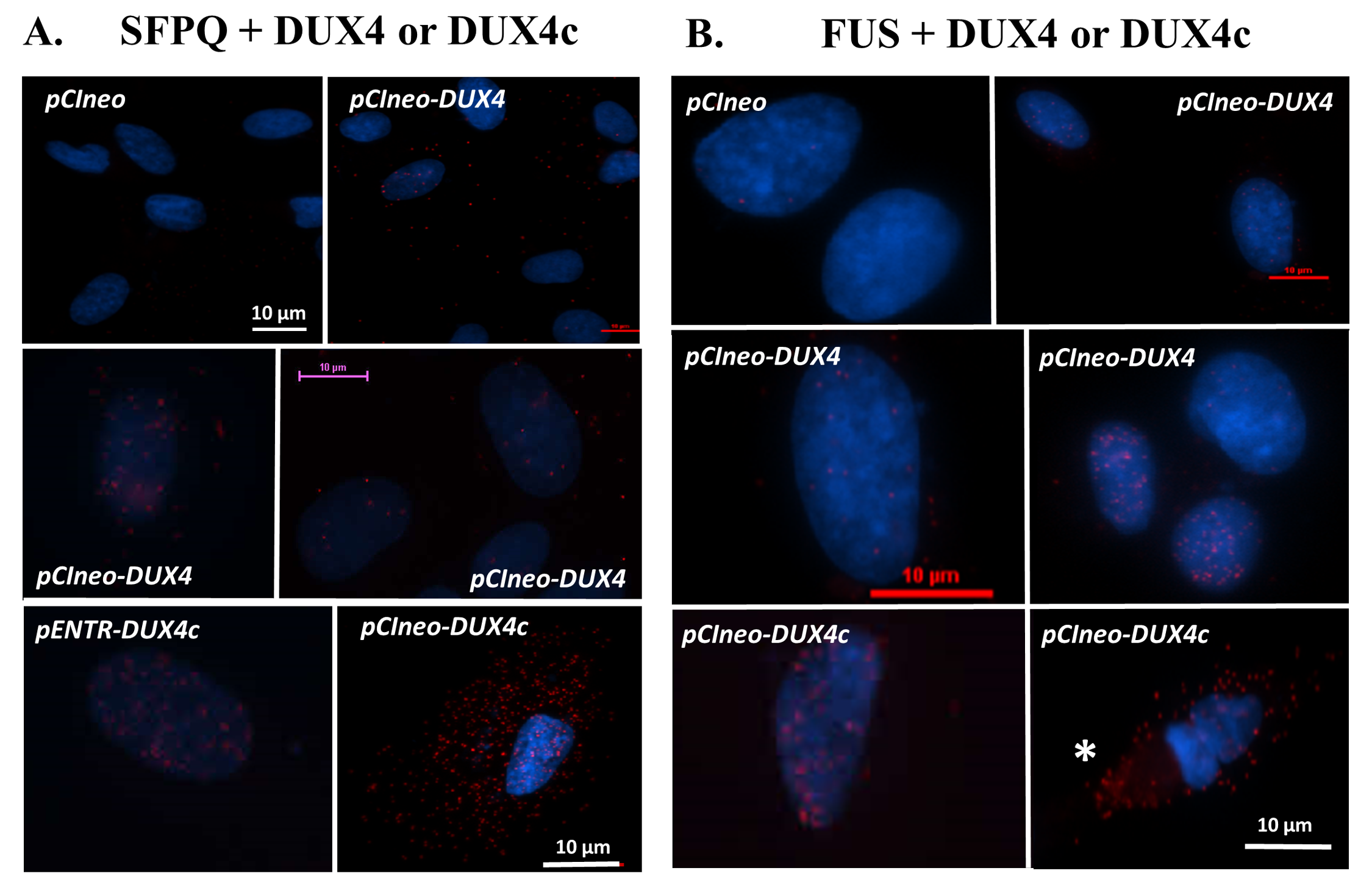

Supplement: S4 Fig — In situ Proximal Ligation Assay (PLA) using antibodies against DUX4 or DUX4c and SFPQ (A) or FUS (B) was performed in healthy myoblasts transfected with a strong DUX4- or DUX4c-expression vector (pCIneo-DUX4 or -DUX4c), the empty parental vector (pCIneo) or a vector with the endogenous promoter (pENTR-DUX4c) as indicated. More interaction spots (red) were detected at or near the nuclear periphery or in the cytoplasm in cells expressing DUX4 or DUX4c. The star (*) points to a high PLA spot density showing DUX4c-FUS interactions at the tip of a myoblast. Scale bar: 10 μm. (TIFF) [file pone.0146893.s004.tiff]

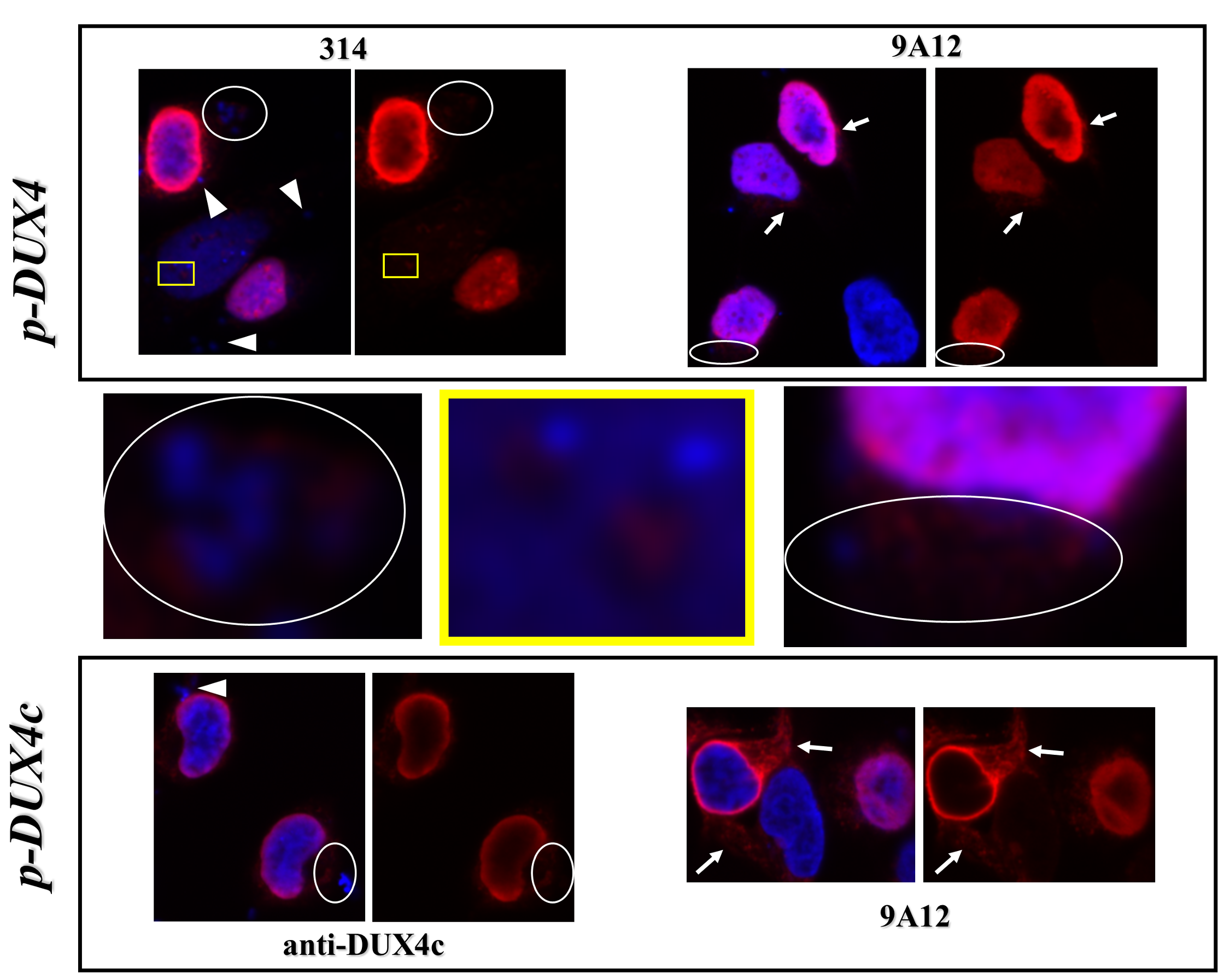

Supplement: S5 Fig — TE671 cells were transfected with the pCIneo-DUX4 (top panel) or -DUX4c (bottom panel) expression vectors. Confocal microscopy analyses were performed on cells immunostained with rabbit anti-DUX4 serum (#314, top left panel) or anti-DUX4c (bottom left panel) or mouse monoclonal anti-DUX4 (9A12, right panels). The nuclei were stained with DAPI (blue). Arrowheads and circles indicate cytoplasmic DAPI staining; arrows and circles indicate DUX4/4c cytoplasmic staining. Magnifications of the circled regions from the top panels are shown in the middle panels (left and right). The yellow box shows nuclear DUX4 staining in regions with low DAPI staining (magnified in the central panel). (TIFF) [file pone.0146893.s005.tiff]

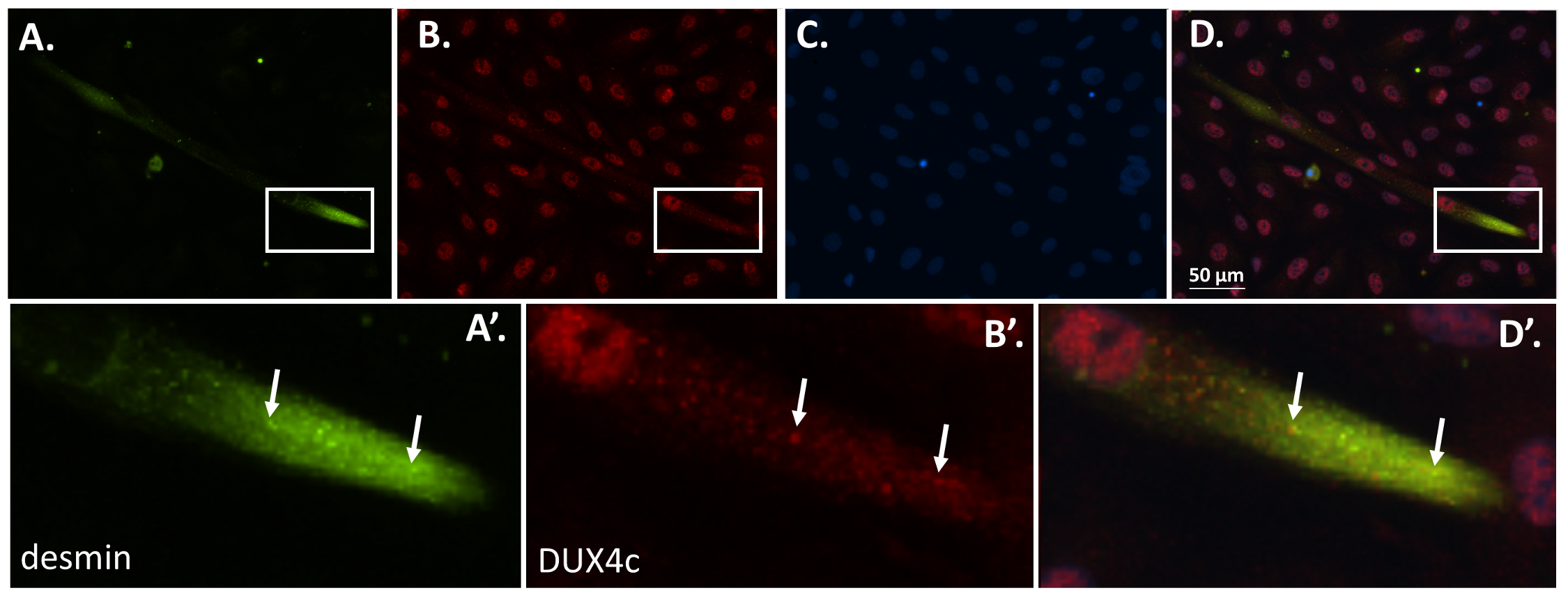

Supplement: S6 Fig — DUX4c (rabbit serum, red) and desmin (mouse monoclonal, green) were detected in an immortalized myoblast line by immunofluorescence. Desmin was concentrated at the tips of an early myotube after 1 day of differentiation (A). This myotube exhibited nuclear as well as cytoplasmic DUX4c staining (B; D). The nuclei were stained with DAPI (C). The accumulation of DUX4c spots was denser in the elongating myotube tips and partially co-localized with desmin (A). Two arrows point to intense DUX4c spots in the boxed myotube tip that was enlarged in (A’,B’,D’). Merged pictures are shown (D,D’). Scale bar: 50 μm. (TIFF) [file pone.0146893.s006.tiff]

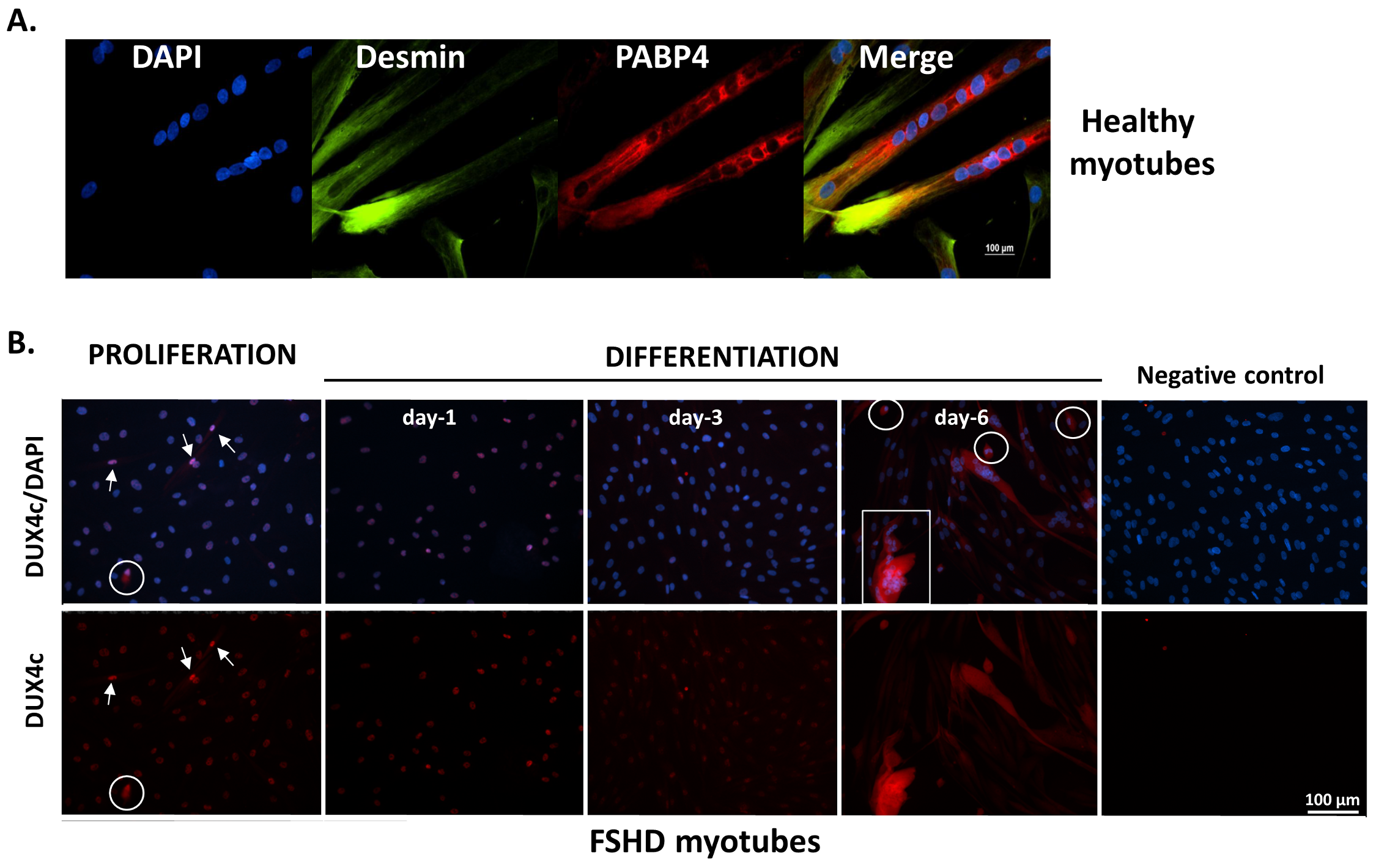

Supplement: S7 Fig — DUX4c was immunodetected in proliferating immortalized myoblasts and during a differentiation time-course. Nuclear staining was observed in almost all nuclei in myoblasts and after one day in the differentiation medium, as in healthy cells but with variable intensities; the more intense nuclear signals are observed in myoblasts showing weak cytoplasmic staining and small nuclei (arrows). Higher cytoplasmic labeling on one side of a cell was also observed in the proliferation medium (circle). During differentiation, DUX4c was progressively detected in the cytoplasm, and the nuclear labeling decreased at day 3. DUX4c nuclear staining was generally lost at day 6, and some myotubes or myoblasts (circles) presented strong cytoplasmic staining. A cluster with a high number of nuclei (boxed) had strong DUX4c labeling in and around the nuclei. This is similar to the DUX4c immunostaining that was observed in FSHD muscle biopsies (Figs 8 and 9) and to the cluster observed in S8 Fig (desmin-DUX4c interaction in DUX4c-overexpressing cells). (TIFF) [file pone.0146893.s007.tiff]

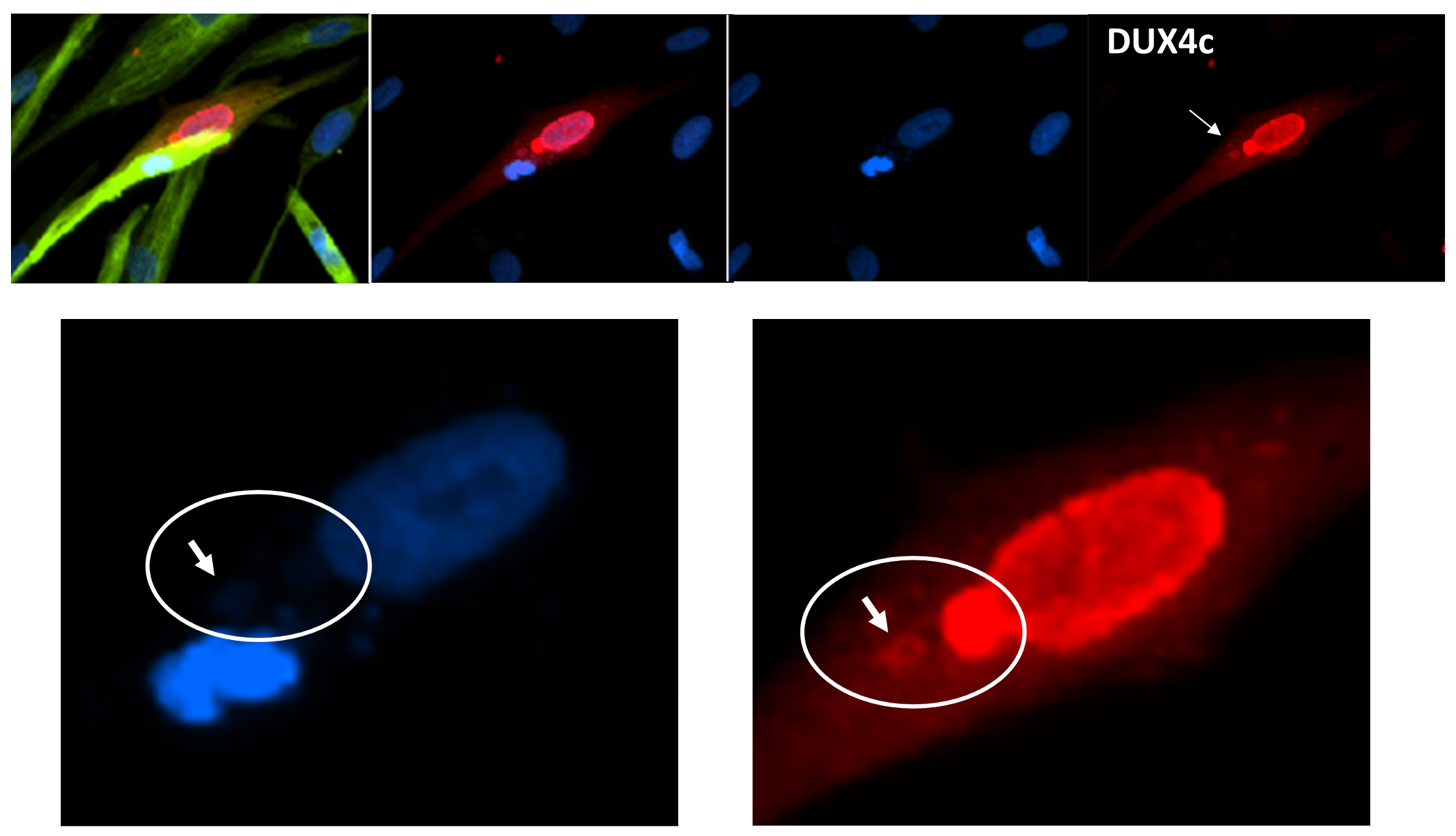

Supplement: S8 Fig — Healthy immortalized myoblasts were transfected with a DUX4c expression vector, and detection of DUX4c (red) and Alpha-tubulin (green) by immunofluorescence was carried out after 6 days in the differentiation medium (as in Fig 7). The nuclei were stained with DAPI. DUX4c was observed at the nuclear periphery (as in S5 Fig). The circle surrounds a nuclear bud containing DUX4c and DAPI staining as well as cytoplasmic DUX4c that co-localized with DAPI staining (indicates by the arrow). (TIFF) [file pone.0146893.s008.tiff]

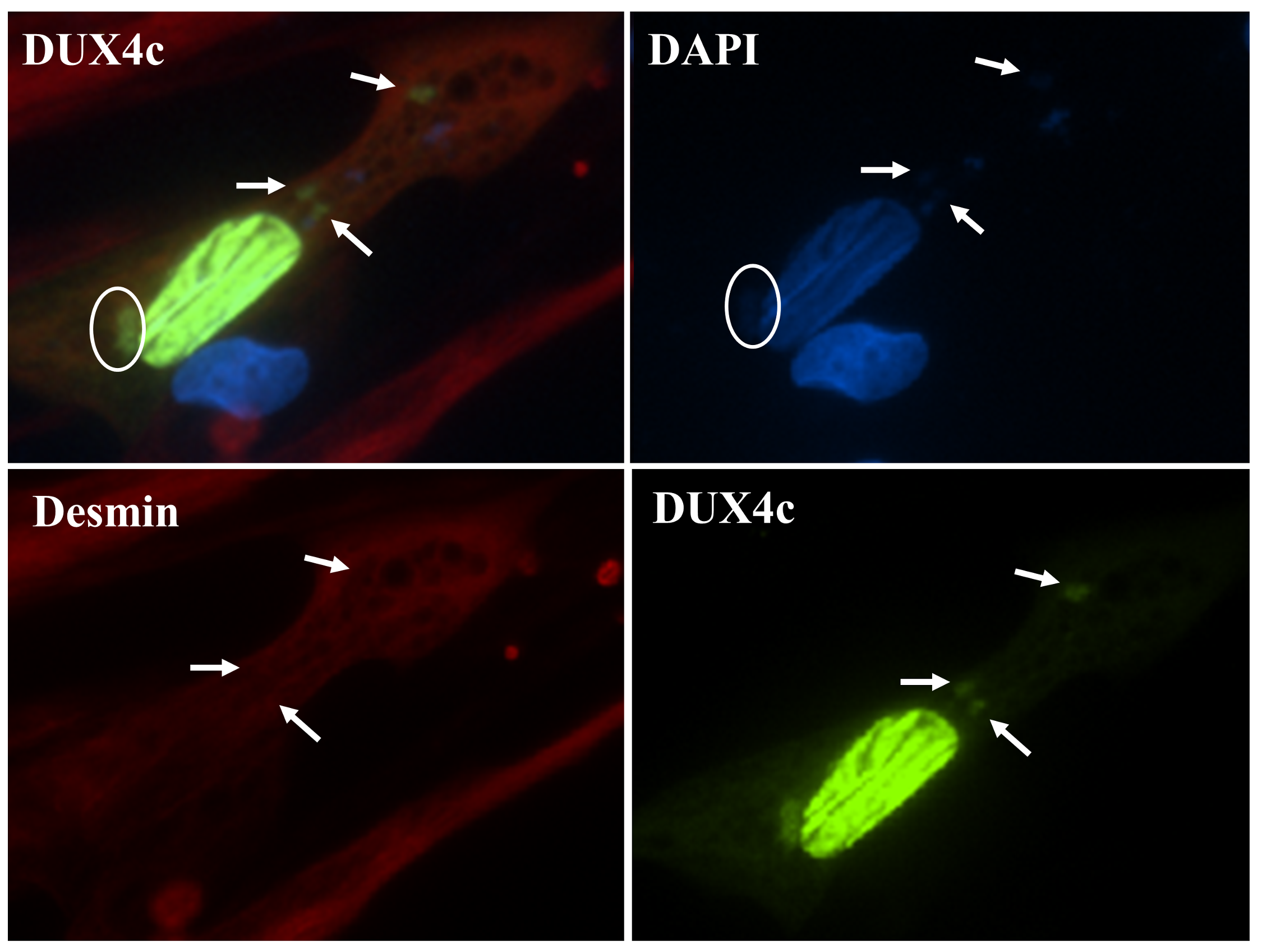

Supplement: S9 Fig — Higher magnification of Fig 7. DUX4c (green) and desmin (red) were detected by immunofluorescence at day 6, and myoblast nuclei were stained with DAPI. Nuclear budding could be observed (circle), and a few cytoplasmic spots (arrows) were stained for DUX4c. The DUX4c nuclear staining presented a pattern of linear stripes that might reflect interactions with the cytoskeleton above the nucleus. (TIFF) [file pone.0146893.s009.tiff]

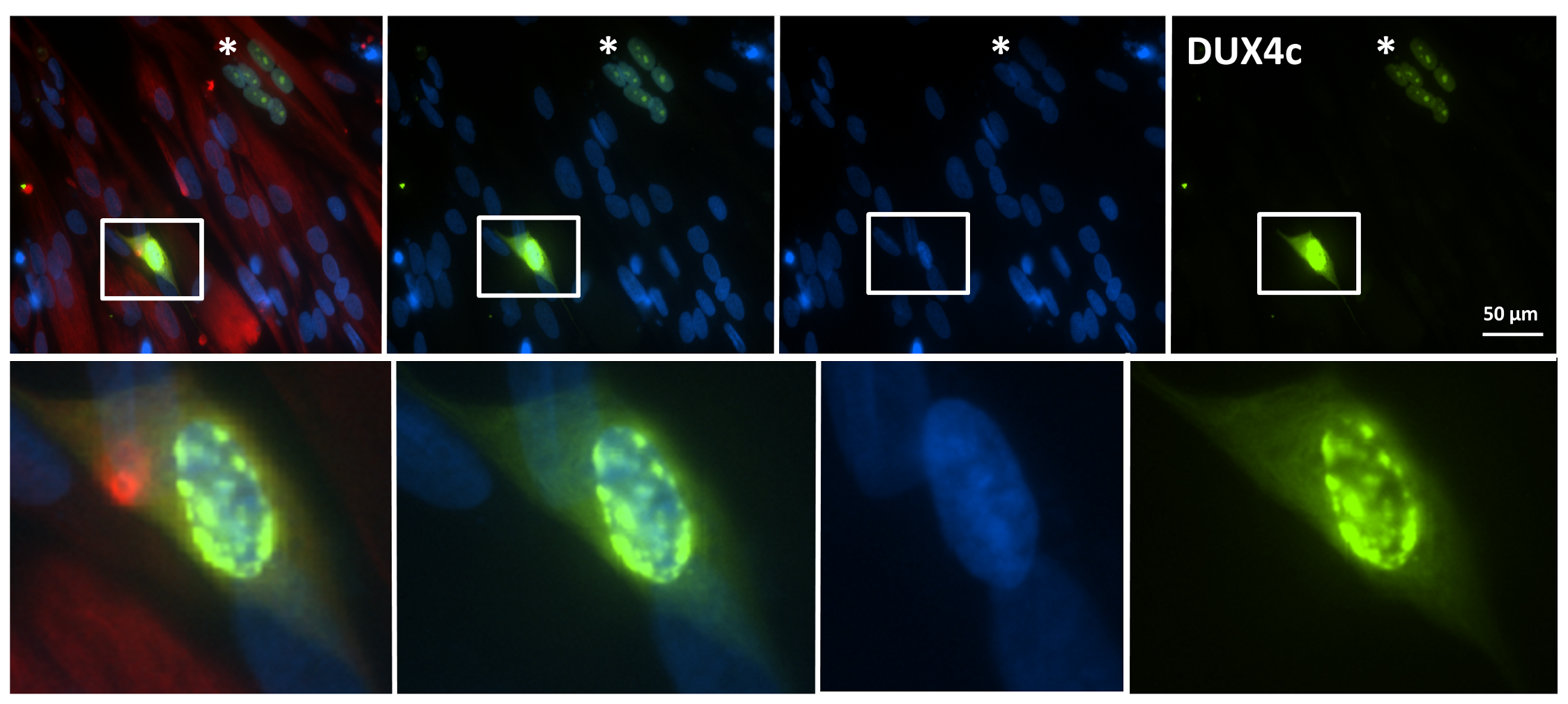

Supplement: S10 Fig — Healthy immortalized myoblasts were transfected with a DUX4c expression vector, and immunodetection was carried out after 6 days in the differentiation medium (as in Fig 7). (Top) A myoblast (boxed) shows nuclear and weak cytoplasmic DUX4c labeling (green). Clusters of nuclei in myotubes (desmin in red) also exhibit a stronger DUX4c staining in regions unstained for DAPI that could be nucleoli (as in S7 Fig*). (Bottom) Magnification of the boxed myoblast showing DUX4c labeling at a lower exposure time in distinct areas of the nuclei, several of them located at the nuclear periphery. (TIFF) [file pone.0146893.s010.tiff]

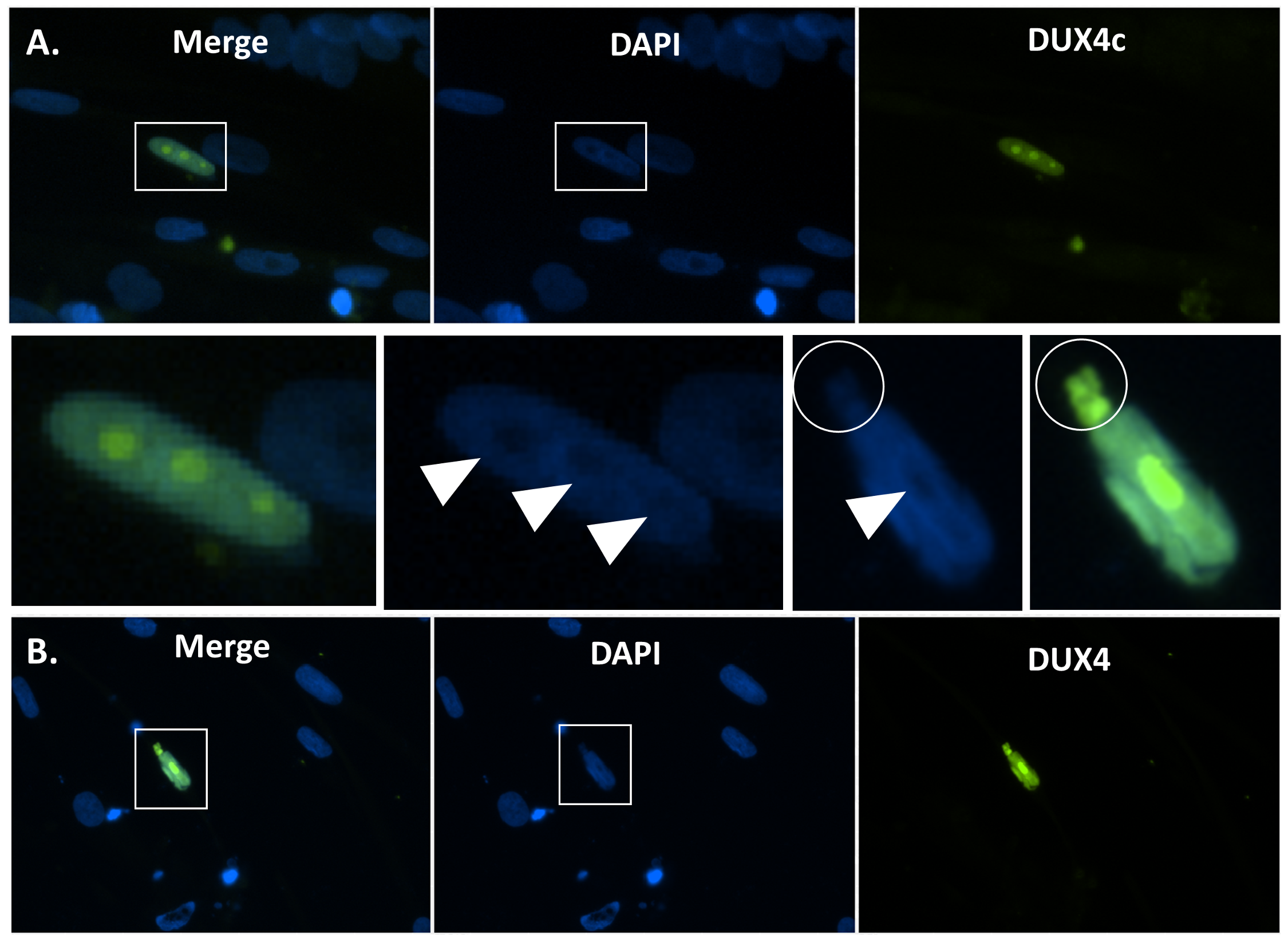

Supplement: S11 Fig — Healthy immortalized myoblasts were transfected with DUX4c (A) or DUX4 (B) expression vectors, and immunofluorescence detection was carried out after 6 days in the differentiation medium (as in Fig 7). In a few nuclei, stronger DUX4 and DUX4c staining was observed in regions unstained for DAPI (arrowheads) that could be nucleoli. Middle panels: magnification of the boxed nucleus (left: DUX4c and DAPI staining) and the circled nuclear bud with strong DUX4 staining (right: DAPI and DUX4 staining). (TIFF) [file pone.0146893.s011.tiff]

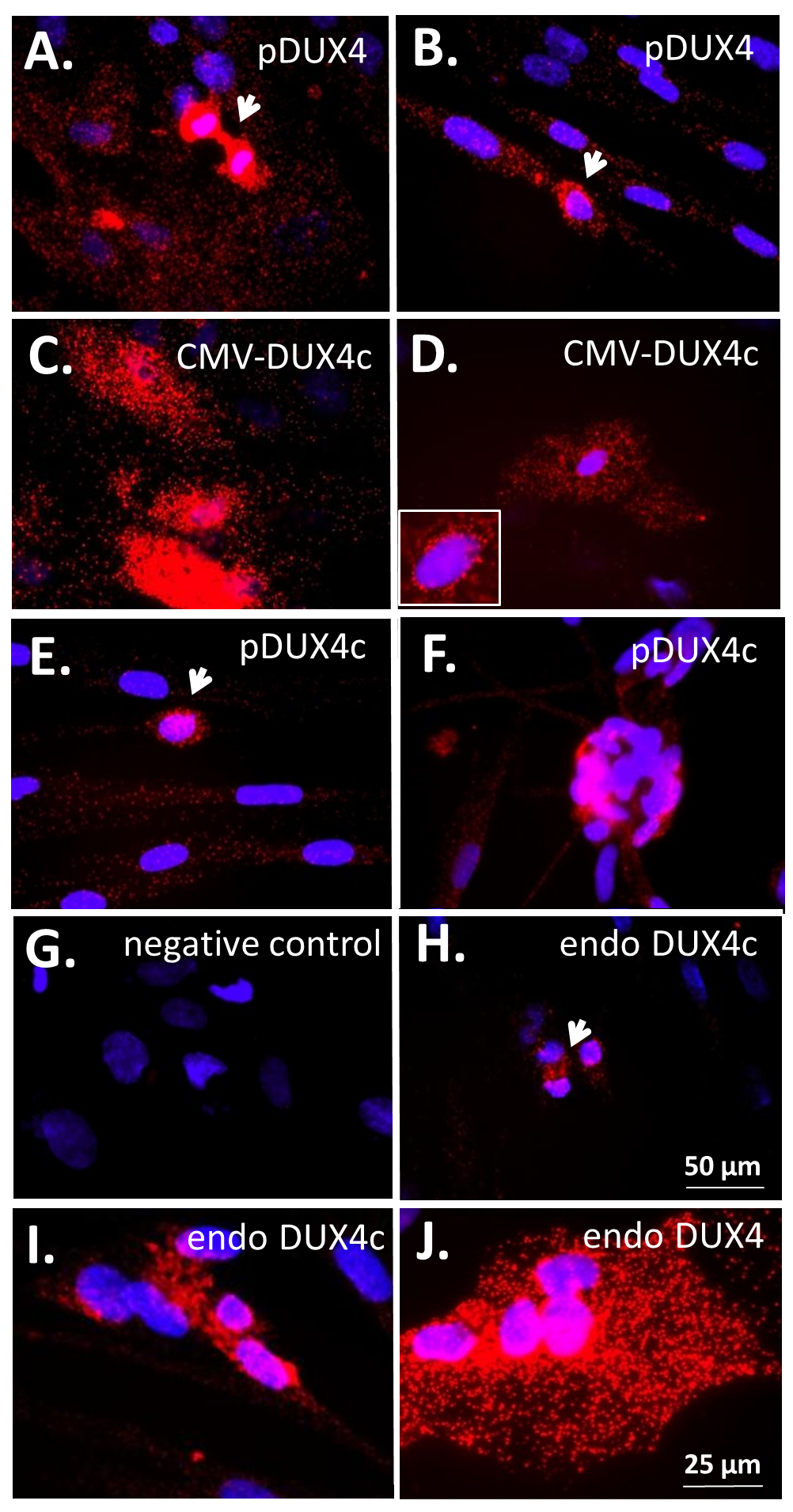

Supplement: S12 Fig — (A-F) Healthy immortalized myoblasts were transfected with vectors expressing DUX4 (A-B) or DUX4c (C-F) under control of the CMV promoter (C-D) or their endogenous promoter (pDUX: A-B, E-F) and fixed either 24 h post transfection (A) or after 4 days in differentiation medium (B-F). (G-J) Untransfected immortalized healthy (G-I) or FSHD (J) myoblasts expressing endogenous DUX4c were differentiated and fixed 4 days later. In situ PLA was performed using the 9A12 mouse MAb to detect DUX4/DUX4c (A-C, F,J) or anti-DUX4c rabbit serum (D-E, G-I) and rabbit or mouse antibodies against desmin, respectively (A-J). The red spots indicate a desmin/DUX interaction, and the nuclei were stained with DAPI. The arrows point to high spot densities close to nuclei. (TIFF) [file pone.0146893.s012.tiff]

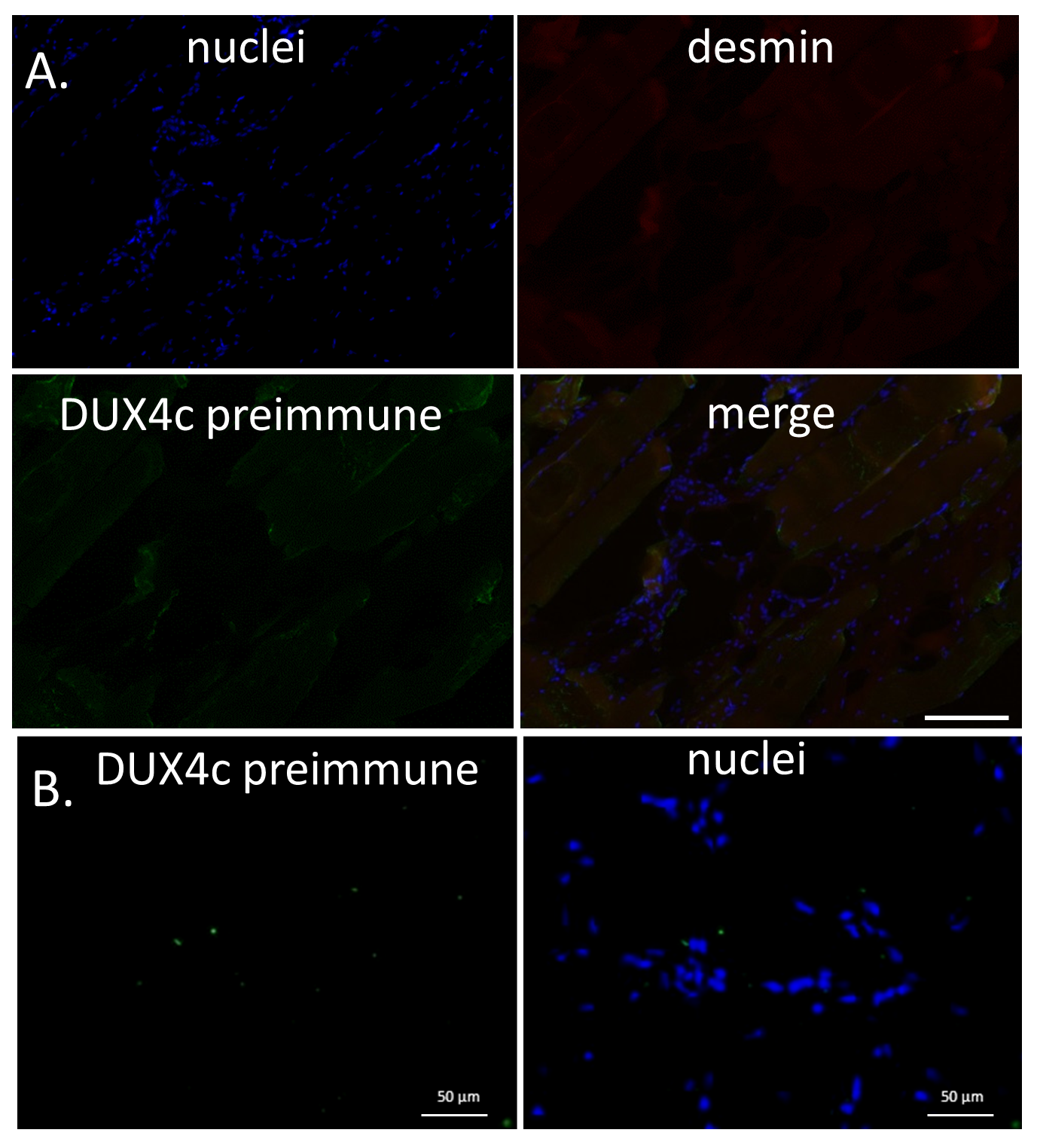

Supplement: S13 Fig — A rabbit preimmune serum was used in place of the anti-DUX4c serum. (A) Longitudinal section adjacent to the one used in Fig 7A–7D. (B) Transverse section adjacent to the one used in Fig 9. (TIFF) [file pone.0146893.s013.tiff]
