# Supplementary material for: Homologous Transcription Factors DUX4 and DUX4c Associate with Cytoplasmic Proteins during Muscle Differentiation
Source: PLoS One. 2016 Jan 27;11(1):e0146893. doi: 10.1371/journal.pone.0146893 (PMC4729438; doi:10.1371/journal.pone.0146893)
Supplement: S1 Table — (PDF) [file pone.0146893.s014.pdf]

S1Table. Primer sequences

#	Orient.	Primer sequence
Yeast-Two-Hybrid constructs		
DUX1	F	5' CGGAATTCGGTTCTGGTTCTGGTTCCCCGTGTCAGTCCGTGAAATTCC 3'
DUX1	R	5' TTCTGCAGCATTGGGGCTGCATTGCAC 3'
DUX1H1	F	5' CGGAATTCGGTTCTGGTTCTGGTTCCCCGTGTCAGTCCGTGAAATTCC 3'
DUX1H1	R	5' TTCTGCAGACAGGGCCGAGATTGCCGC 3'
DUX1H2	F	5' CGGAATTCGGTTCTGGTTCTGGTAACCCGCAAAAAGGCAGAC 3'
DUX1H2	R	5' TTCTGCAGCATTGGGGCTGCATTGCAC 3'
DUX4	F	5' CGGAATTCGGTTCTGGTTCTGGTTCGCGTCCGTCCGTGAAATTCC 3'
DUX4	R	5' TTGGATTCATCACCGGGCCTAGACCTAGAA 3'
DUX4H1	F	5' CGGAATTCGGTTCTGGTTCTGGTTCGCGTCCGTCCGTGAAATTCC 3'
DUX4H1	R	5' TTCTGCAGGGGCCAGGGCCGAGATTC 3'
DUX4H2	F	5' CGGAATTCGGTTCTGGTTCTGGTAGACGCGGCCCGCCAGAAG 3'
DUX4H2	R	5' TTCTGCAGGGCGACCCACGAGGGAGCAG 3'
DUX4t	F	5' CGCAATTCGGTTCTGGTTCTGGTAGTCACCCTGCTCCCTCGT 3'
DUX4t	R	5' TTGGATTCATCACCGGGCCTAGACCTAGAA 3'
DUX4c	F	5' CGGTTCGGTTCTGGTTCTGGTTCGCGTCCGTCCGTGAAATTCC 3'
DUX4c	R	5' CGGAATTCCAGAGCGGGCTCTTCCAC 3'
DUX4ct	F	5' CGGAATTCGGTTCTGGTTCTGGTAGTCACCCTGCTCCCTCGT 3'
DUX4ct	R	5' CGCGGATCCCAGAGCGGGCTCTTCCAC 3'
pENTR1A-DUX4c construct		
DUX4c	F	5' CACCATGGCCCTCCCGACACCCT 3'
DUX4c	R	5' ATAGTTTAGCGGCCGCTGGTGTCTACAGGAGCTCAT 3'
HaloTag constructs		
DUX4c	F	5' ATATGCGATCGCCATGGCCCTCCCGACACCTCC 3'
DUX4c	R	5' ACGCGTTTAAACCAGGAGCTCATATAACAGGCTGGAGGTG 3'
DUX4	F	5' GGCTGCGATCGCCATGGCCCTCCCGACACCCTC 3'
DUX4	R	5' GTCGGTTTAAACAAGCTCCTCCAGCAGAGCCC 3'
DUX4term	F	5' TAAAGCGATCGCCATGCAAGGCATCCCGGCGCC 3'
DUX4term	R	5' GTCGGTTTAAACAAGCTCCTCCAGCAGAGCCC 3'
EGFP	F	5' AGGAGCGATCGCCATGGTGAGCAAGGGCGAGGAG 3'
EGFP	R	5' ATGGGTTTAAACCTTGTACAGCTCGTCCATGCCG 3'

Underlined: a sequence encoding a GSGSG peptide was introduced between the two parts of the hybrid construct to act as a flexible arm; this would allow the construct to avoid steric hindrance and allow optimal protein folding. All constructs were verified by DNA sequencing. t : tail
