# Supplementary material for: Homologous Transcription Factors DUX4 and DUX4c Associate with Cytoplasmic Proteins during Muscle Differentiation
Source: PLoS One. 2016 Jan 27;11(1):e0146893. doi: 10.1371/journal.pone.0146893 (PMC4729438; doi:10.1371/journal.pone.0146893)
Supplement: S2 Table — (PDF) [file pone.0146893.s015.pdf]

S2 Table. Summary of desmin/DUX reconstructions in yeast.

+++: yeast lawn, ++: 10 to 100 colonies, +: 5 to 10 colonies, -: 0 colonies. AT: 3-Aminotriazole/agar

**Selective medium**	**Yeast transformed with *pGAD424-GAL4-AD-desmin***						
	pGBT9	***pGBT9-Pase s.u.***	***pGBT9-DUX1H1***	***pGBT9-DUX1H2***	***pGBT9-DUX1***	***pGBT9-DUX4H1***	***pGBT9-DUX4H2***
**ScαTαL**	+++	+++	+++	+++	+++	+++	+++
**ScαTαLαH+2 mM AT**	-	-	++	-	+++	-	-

Yeast PJ69-4A (*Trp1*$^{-}$, *Leu2*$^{-}$, *His3*$^{-}$, *Ade2*$^{-}$) were grown on medium without tryptophan and leucine (ScαTαL) to select yeast cells that were transformed with both plasmids. The selected yeast was then grown on identical medium without histidine (ScαTαLαH) in the presence of aminotriazol (AT) to inhibit the basal level of the His3 enzyme. Yeast growth (+) corresponds to the activation of the *GAL4-HIS3* reporter gene. Negative controls: *pGBT9* and p*GBT9* encoding a phosphatase subunit (Pase s.u.). Note that the DUX4H1 and DUX4H2 sequences are identical to DUX4cH1 and DUX4cH2.
