# Supplementary material for: Homologous Transcription Factors DUX4 and DUX4c Associate with Cytoplasmic Proteins during Muscle Differentiation
Source: PLoS One. 2016 Jan 27;11(1):e0146893. doi: 10.1371/journal.pone.0146893 (PMC4729438; doi:10.1371/journal.pone.0146893)
Supplement: S3 Table — (PDF) [file pone.0146893.s016.pdf]

S3 Table. DUX4 partners identified by HaloTag co-purification in human TE671 and LHCN-M2

	HaloTag fusion protein	DUX4 1-424	DUX4term 349-424	DUX4c 1-374
**Function**		**number of identified peptides**		
**Cytoskeletal organization (GO0006996: organelle organization)**				
Uniprot nb	**Actin and actin-associated proteins**			
Q9BTI9	*¶NPM1, nucleophosmin	21 (C)		
F8W7K3	*SPTAN1 spectrin alpha-chain, subunit of fodrin	17 (C)		
D6W5C0	*SPTBN1 Spectrin, beta-chain, subunit of fodrin	6 (C)		
Q53SB5	Desmin, isoform CRA_a	14 (C)		
Q13707	¶Actin, ACTA2 protein			5 (N)
Q6UYC3	Lamin A/C, LMNA<br>Only peptides common to lamin A and C were identified	5 (C)		
Q1HE25	Actinin alpha 1 isoform b, ACTN1	2 (C)		
B4DM97	*highly similar to α-centractin/ARP1	2 (C)		
F8WCF6	*ARPC4 (actin-related protein 2/3 complex, subunit 4) and TTLL3 (tubulin tyrosine ligase-like family, member 3), fusion protein	2 (C)		
Q6FI97	Actin-Like 6A/ARP4	2(C)		
Q5T0I2	Gelsolin, GSN	2 (C)		
Q60FE2	¶MYH9 (non-muscle MHC-IIA) variant protein	9 (C)		
B7Z6Z4	MYL6		1(C)	
F2Z2D5	POF1B, non-muscle	4 (C)		
Q53R15	MYL1, myosin, light chain 1	2 (C)	3 (C)	
Q5VU72	Tropomyosin 3, TPM3, non-muscle	2 (C)		
B5BU28	CTNNB1, Catenin beta 1	4 (C)		
E7ETZ0	CALM1, calmodulin	3 (C)		
Q9BRL5	CALM3, calmodulin		2 (C)	
Q5VXV3	SET	2(C)		
C9JPM4	§ADP-Ribosylation Factor 41, ARF4	2(C)		2(C)
	**Microtubule-associated and centrosomes organization**			
Q9H2R7	* Nuclear Distribution Protein, NUDC	2(C)		
A4D177	Chromobox homolog 3, CBX3	2(C)		
B5MDF5	GTP-binding nuclear protein RAN	5(C)		
Q06S70	*TREX2	2(C)		
* NPM1, ARP1, fodrin and Catenin beta 1		see above*		
**protein folding (GO:0006457)**				
	**Actin and tubulin folding**			
F5H282	§Chaperonin containing TCP1 subunit alpha, CCTA	5 (C)		3 (C)
B7ZAT2	TCP1, subunit beta, isoform	2 (C)		
F8VQ14	TCP1, subunit beta, CCT2			7 (C)
Q5SZY0	TCP1 subunit gamma, CCT3	7 (C)		5 (C)
F5H5W3	TCP1 subunit delta, CCT4	4 (C)		

B7Z9L0	TCP1 subunit delta, CCT4			5 (C)
A8K2X8	highly similar to TCP1 subunit epsilon, CCT5			5 (C)
B4DDU6	highly similar to TCP 1 subunit epsilon	3 (C)		
Q59ET3	TCP1 subunit 6A isoform a variant	3 (C)		
Q53HV2	§TCP1 subunit eta variant	4 (C)		
Q6IBT3	TCP1 subunit eta, CCT7			3 (C)
Q7Z759	TCP1 subunit theta, CCT8	7 (C)		
Q53HU0	TCP1 subunit theta variant			7 (C)
	**Others**			
Q5CAQ5	HSP90B1, Heat Shock Protein 90 kDa Beta (Grp94), Member 1	18 (C)		9 (C)
Q53RC7	disulfide isomerase family A, member 6, isoform	8 (C)		
B4DQ50	highly similar to Protein-glutamine gamma-glutamyltransferase E	2(C)		
**RNA-associated proteins**				
	**Transcriptional regulation**			
Q86VG2	*SFPQ, Splicing factor proline/glutamine-rich**	8 (C)		3 (N)
F5GYZ3	NONO, Non-POU Domain Containing, Octamer-Binding1**	2 (C)		
B5BUE6	*¶DDX5, DEAD box polypeptide 5	6 (C)		4 (C)
Q59F66	*DDX17, DEAD box polypeptide 17 isoform p82 variant	2 (C)		
Q6FHQ0	RBBP4/ RbAp48**	3 (C)		
Q6FHQ0	RBBP7**	4 (C)		
F4ZW66	&*¶ILF3, Interleukin enhancer-binding factor 2 variant (90 kDa NFAT subunit)	5 (C)		
Q53FG3	&*¶ILF2 (45 kDa NFAT subunit)	2 (C)		
B3KRS5	HDAC2**	2 (C)		
Q59EB5	*CSDA protein variant	5 (C)		
	**Splicing regulation**			
B2R8Z8	&Similar to SYNCRIP, hnRNP Q	7 (C)		
Q8TBR3	&FUS, hnRNP P2	3 (C)		1 (N)
B4DZZ1	MYBBP1 nucleolar transcriptional regulator	2 (C)		
A8K651	*¶C1QBP, p32 subunit of splicing factor 2 (SF2)	5 (C)		2 (N)
B4DUA4	§SRSF5, Serine/arginine-rich-splicing factor 5	2 (C)		
Q49AN9	SNRPG, small nuclear ribonucleoprotein G (snRNP)	2 (C)		
*SFPQ, NONO, DDX5, DDX17, NMP1, NFAT		* See above		
	**RNA stability**			
Q6IQ30	¶PABPC4, Poly(A)-binding protein 4	14 (C)		
E7ERJ7	&¶PABPC1, Poly(A)-binding protein 1		4 (C)	
B3KT93	highly similar to Poly(A)-binding protein 1	14 (C)		
Q53SS8	PCBP1, Poly(RC)-binding protein 1, hnRNP E1/X	7 (C)	1 (C)	2 (C)
Q6IPF4	&PCBP2, Poly(RC)-binding protein 2, hnRNP E2	5 (C)		3 (C)

B4DVB8	&*Similar to ELAV-like 1	3 (C)		
*NFAT		* See above		
	**mRNA and mRNP granules export to the cytoplasm,**			
C9JT33	&¶IGF2BP1, IGF2 mRNA-binding protein 1	5(C)		
D3DTW3	&IGF2BP1, IGF2 mRNA-binding protein 1, isoform CRA	7 (C)		
*NFAT, DDX5, FUS, SFPQ, NONO, TREX2, RAN		* See above		
	**Translational regulation**			
A8K7F6	highly similar to EIF4A1, eukaryotic translation initiation factor 4A, isoform 1	2 (C)		6 (C)
Q53HK3	EIF2, Eukaryotic translation initiation factor 2, subunit 3 gamma, 52 kDa variant	5 (C)		
Q96G38	EIF3B, Eukaryotic translation initiation factor 3, subunit beta	4 (C)		
Q5U0F4	EIF3I, Eukaryotic translation initiation factor 3, subunit 2 beta, 36 kDa	4 (C)		
Q6IAM0	EIF3S4, Eukaryotic translation initiation factor 3 subunit G	4 (C)		
Q24JU4	EIF3A, Eukaryotic translation initiation factor 3, subunit A	3 (C)		
F5H425	EIF3J	3 (C)		
B3KXU8	*Highly similar to CAPRIN 1, Cell Cycle-Associated Protein 1	3 (C)		
E9PD14	*GNB2L1, Guanine Nucleotide-Binding Protein (G Protein), Beta Polypeptide 2-Like			3 (C)
*CSDA protein variant, IGF2BP1		*See above		
	**Ribosome biogenesis**			
Q3KQS4	*NOP2 nucleolar protein	2 (C)		
*NPM1, DDX5, DDX17, C1BPQ, MYBBP1A		*See above		
	**Ribosomal proteins**			
Q6NXR8	&¶Ribosomal protein S3A	9(C)		
Q9NY85	¶Ribosomal protein L3 (Fragment)	8(C)		
A9C4C1	&¶Ribosomal protein S9	5(C)		
Q5T8U4	¶Ribosomal protein L7a	7(C)		
E5RI99	¶Uncharacterized protein GN=RPL30	8(C)		
E7EQV9	¶Ribosomal protein L15	4(C)		
B7Z4K2	¶Ribosomal protein L31, isoform CRA_c	4(C)		
A8MUD9	¶Uncharacterized protein GN=RPL7	3(C)		
B2R4D8	¶60S ribosomal protein L27	5(C)		
Q96IR1	&¶RPS4X protein (Fragment)	2(C)		2(C)
E9PKZ0	¶Uncharacterized protein GN=RPL8	3(C)		
Q59GY2	¶Ribosomal protein L4 variant (Fragment)	3(C)		
Q5JR94	&¶40S ribosomal protein S8	2(C)		
Q9HBB3	¶60S ribosomal protein L6	2(C)		
Q53G25	Ribosomal protein S5 variant (Fragment)	2(C)		
Q53G83	¶Ribosomal protein S3 variant (Fragment)	2(C)		5(C)
A8MUS3	¶Ribosomal protein L23a, isoform CRA_a	2(C)		
Q5VVD0	Ribosomal protein L11	3(C)		
Q59FS3	¶Ribosomal protein S7 variant (Fragment)	2(C)		
Q6IPH7	RPL14 protein	2(C)		

Q9BSW5	RPS2 protein (Fragment)			2(C)
Q7Z4W8	Heparin-binding protein HBp15, Ribosomal protein RPL22	7(C)		
Q96RS2	Ribosomal Protein SA Pseudogene 151, LAMR1P15	2(C)		
Q5MK14	Leukemia-associated protein	2(C)	1(C)	
*GNB2L1		*see above		
	***TP53* regulation**			
*NPM1, Similar to ELAV-like 1, NOP2		*See above		
	**Neuronal differentiation**			
B5MCE7	BZW2, Basic leucine zipper and W2 domains 2, isoform CRA_b	3 (C)		
Q8N274	Similar to AHNAK	4 (C)		
B7Z9Y9	Weakly similar to Paraneoplastic Ma Antigen 2	11(C)		
*NFAT, similar to CAPRIN1, NUDC, SFPQ		*See above		
	**other**			
A8K0N0	SRP9	2(C)		
**WNT signaling**				
*Catenin beta 1, NFAT, GNB2L1, DDX5		* See above		
**Oxidative stress**				
B2R4P2	highly similar to Peroxiredoxin-1	8 (C)	3 (C)	4 (C)
B4DF70	highly similar to Peroxiredoxin-2	8 (C)	2 (C)	4 (C)
A8MX94	GSTP1		3(C)	
**Others**				
	**transcription**			
P52926	[§]HMGA2	2(N)		1(N)
B1AHC7	[¶]XRCC6	3(C)		
	**translation**			
B4DQY1	highly similar to Serine/threonine-protein phosphatase 2A 65 kDa regulatory subunit A alpha	3(C)		
Q6ZTI3	highly similar to Lysyl-tRNA synthetase	6(C)		
B4E266	highly similar to Leucyl-tRNA synthetase, cytoplasmic	2(C)		
	**proteasome**			
Q6IBM2	Proteasome (Prosome, Macropain) Activator Subunit 1, PSME1	5(C)		
Q05DH1	PSMA7	2(C)		2(C)
Q6IAT9	PSMB6	2(C)	1(C)	
Q5TZN3	UBE2C	2(C)		
Q5UGI3	UBC, ubiquitin C splice variant			2(C)
E9KL27	Epididymis tissue protein Li 174	2(C)		
B4DXJ9	PSMA6	2(C)		
Q96IF9	VCP	2(C)		
	**Extracellular(?), membrane proteins and membrane vesicle trafficking**			
B4DIN1	[§]Clathrin light chain A, CLTA	3(C)		
B2R4M6	highly similar to Homo sapiens S100 calcium-binding protein A9	11(C)	2(C)	
	**others**			

B4DUU6	¶Pyruvate kinase, PKM	14(C)		8(C)
B7Z5E3	¶L-lactate dehydrogenase, LDHA	8(C)		5(C)
Q5U077	L-lactate dehydrogenase, LDHB			8(C)
Q75MT9	Malate dehydrogenase, MDH2			4(C)
Q5SZU1	Phosphoglycerate dehydrogenase, PHGDH			2(C)
B2R721	FLJ93240, highly similar to insulin-degrading enzyme (IDE)	3(C)		
A8K008	cDNA FLJ78387	4(C)		
B4DE76	highly similar to Ran-specific GTPase-activating protein	3(C)		
Q6FIG4	RAB1A	2(C)		
Q6FI42	RAB11B		3(C)	
C9JQV0	Uncharacterized Protein C7orf502	6(C)		
B7ZLF8	Uncharacterized protein, cytoplasmic	4(C)		
Q5EFE6	Anti-RhD monoclonal T125 kappa light chain	2(C)		

Scaffold Viewer settings summary: protein threshold 95%, min # peptides 2, peptide threshold 95%

GO: gene ontology

Any peptide of the proteins identified were found in the control HaloTag-EGFP co-purification.

Table data represent all the identified proteins from 7 co-purifications experiments (2 with HaloTag-DUX4, 4 with HaloTag-DUX4c and 1 with HaloTag-DUX4term realized each time in parallel with the control HaloTag-EGFP). **We always found C1QBP, SFPQ and FUS in each HaloTag-DUX4 or -DUX4c co-purifications.**

We found many peptides from different **tubulin** forms in each conditions. However, fewer peptides corresponding to conserved regions were found following HaloTag-EGFP co-immunoprecipitation. In contrast, more peptides in non-conserved regions were retrieved following HaloTag-DUX4, -DUX4term or -DUX4c co-immunoprecipitation.

* Involved in different functions: some are depicted in the table.

**The heterodimers SFPQ/NONO and RBBP4/RBBP7 are involved in transcriptional repression by association with HDAC.

¶ Identified (or highly similar) as putative DUX4 partner using GST pull-down assays in human myoblasts or HEK293 cells (see S4 Table)

§ Isoform (or similar function) identified in other approaches (see other tables)

& Known to be associated in IGF2BP1-dependent mRNP granules
