# Supplementary material for: Homologous Transcription Factors DUX4 and DUX4c Associate with Cytoplasmic Proteins during Muscle Differentiation
Source: PLoS One. 2016 Jan 27;11(1):e0146893. doi: 10.1371/journal.pone.0146893 (PMC4729438; doi:10.1371/journal.pone.0146893)
Supplement: S4 Table — (PDF) [file pone.0146893.s017.pdf]

Supplemental Table 4

	Function	DUX4<br>human myoblasts	DUX4<br>HEK293		
Uniprot ID	Actin-associated protein	number of identified peptides		Gene ontology function	Gene ontology ID
Q13885	Tubulin beta-2A chain	2	0	microtubule family cytoskeletal protein	PC00157
P07437	Tubulin beta chain	18	16	microtubule family cytoskeletal protein	PC00157
P35580	Myosin-10	0	16	actin-binding motor protein	PC00040
P63261	[¶]Actin, cytoplasmic 2	9	0	actin and actin related protein	PC00039
P21333	Filamin-A	7	0	actin cytoskeleton	GO:0015629
Q13509	Tubulin beta-3 chain	2	1	microtubule family cytoskeletal protein	PC00157
P14649	Myosin light chain 6B	4	1	actin cytoskeleton	GO:0015629
Q9BUF5	Tubulin beta-6 chain	2	0	microtubule family cytoskeletal protein	PC00157
P08670	Vimentin	11	3	intermediate filament	PC00129
Q14204	Cytoplasmic dynein 1 heavy chain 1	5	3	microtubule family cytoskeletal activity	PC00157
P35579	[¶]Myosin heavy chain 9	41	10	actin-binding motor protein	PC00040
P06748	[¶]Nucleophosmin	2	5	Organelle organization<br>RNA metabolic process	GO:0006996<br>GO:0016070
P84077	[§]ADP-ribosylation factor 1	2	0	Organelle organization<br>hydrolase activity	GO:0006996<br>GO:0016787
	**RNP complex**				
P46781	[&¶]40S ribosomal protein S9	2	0	ribonucleoprotein complex	GO:0030529
P62753	40S ribosomal protein S6	2	0	ribonucleoprotein complex	GO:0030529
P62847	40S ribosomal protein S24	2	1	ribonucleoprotein complex	GO:0030529
P62917	[¶]60S ribosomal protein L8	2	1	ribonucleoprotein complex	GO:0030529
P62750	[¶]60S ribosomal protein L23a	2	1	ribonucleoprotein complex	GO:0030529

P62081	[¶]40S ribosomal protein S7	0	2	ribonucleoprotein complex	GO:0030529
P61247	[&¶]40S ribosomal protein S3a	4	2	ribonucleoprotein complex	GO:0030529
P62906	60S ribosomal protein L10a	3	2	ribonucleoprotein complex	GO:0030529
P32969	60S ribosomal protein L9	3	2	ribonucleoprotein complex	GO:0030529
P62244	40S ribosomal protein S15a	3	2	ribonucleoprotein complex	GO:0030529
P62841	40S ribosomal protein S15	2	2	ribonucleoprotein complex	GO:0030529
P84098	60S ribosomal protein L19	2	2	ribonucleoprotein complex	GO:0030529
P46776	[¶]60S ribosomal protein L27a	1	2	ribonucleoprotein complex	GO:0030529
P46783	40S ribosomal protein S10	1	2	ribonucleoprotein complex	GO:0030529
P60866	40S ribosomal protein S20	1	2	ribonucleoprotein complex	GO:0030529
P62249	[¶]40S ribosomal protein S16	0	2	ribonucleoprotein complex	GO:0030529
P15880	40S ribosomal protein S2	2	2	ribonucleoprotein complex	GO:0030529
P62899	[¶]60S ribosomal protein L31	2	2	ribonucleoprotein complex	GO:0030529
P62829	60S ribosomal protein L23	2	2	ribonucleoprotein complex	GO:0030529
P62269	40S ribosomal protein S18	4	4	ribonucleoprotein complex	GO:0030529
P26373	60S ribosomal protein L13	4	4	ribonucleoprotein complex	GO:0030529
P62241	[&¶]40S ribosomal protein S8	4	4	ribonucleoprotein complex	GO:0030529
P62888	[¶]60S ribosomal protein L30	3	4	ribonucleoprotein complex	GO:0030529
P62263	40S ribosomal protein S14	3	4	ribonucleoprotein complex	GO:0030529
P62277	40S ribosomal protein S13	3	4	ribonucleoprotein complex	GO:0030529
P18621	60S ribosomal protein L17	2	4	ribonucleoprotein complex	GO:0030529
P36578	[¶]60S ribosomal protein L4	1	4	ribonucleoprotein complex	GO:0030529
P62851	40S ribosomal protein S25	3	3	ribonucleoprotein complex	GO:0030529
Q9Y3U8	60S ribosomal protein L36	3	3	ribonucleoprotein complex	GO:0030529
P62424	[¶]60S ribosomal protein L7a	2	3	ribonucleoprotein complex	GO:0030529
P18124	[¶]60S ribosomal protein L7	6	5	ribonucleoprotein complex	GO:0030529
P39023	[¶]60S ribosomal protein L3	6	4	ribonucleoprotein complex	GO:0030529
P62701	[&¶]40S ribosomal protein S4, X isoform	5	3	ribonucleoprotein complex	GO:0030529
P300050	60S ribosomal protein L12	4	3	ribonucleoprotein complex	GO:0030529

P27635	60S ribosomal protein L10	4	3	ribonucleoprotein complex	GO:0030529
Q07020	60S ribosomal protein L18	1	3	ribonucleoprotein complex	GO:0030529
P61313	[¶]60S ribosomal protein L15	1	2	ribonucleoprotein complex	GO:0030529
P46778	60S ribosomal protein L21	1	2	ribonucleoprotein complex	GO:0030529
P42766	[¶]60S ribosomal protein L35	2	3	ribonucleoprotein complex	GO:0030529
P35268	[¶]60S ribosomal protein L22	2	3	ribonucleoprotein complex	GO:0030529
P23396	[&¶]40S ribosomal protein S3	6	4	ribonucleoprotein complex	GO:0030529
P0CW22	40S ribosomal protein S17-like	5	3	ribonucleoprotein complex	GO:0030529
Q02878	[¶]60S ribosomal protein L6	1	3	ribonucleoprotein complex	GO:0030529
	**Transcription-related protein**				
Q6NZI2	Polymerase I and transcript release factor	4	0	transcription cofactor	PC00218
P09429	High mobility group protein B1	2	0	transcription cofactor	PC00218
Q9BQG0	[¶]Myb-binding protein 1A	0	2	transcription factor	PC00218
Q86VP6	Cullin-associated NEDD8-dissociated protein 1	0	3	transcription factor	PC00218
P67809	Nuclease-sensitive element-binding protein 1	4	5	regulation of transcription	GO:0006355
Q12906	[&¶]Interleukin enhancer-binding factor 3, ILF3	0	3	transcription factor<br>immune system process	PC00218<br>GO:0002376
	**RNA processing**				
A8K651	[*#¶]Complement component 1 Q subcomponent-binding protein	7	11	apoptotic process, mRNA processing, immune response, ribosome biogenesis	GO:0006915, GO:0006397, GO:0006955, GO:0042254
Q00839	[&]Heterogeneous nuclear ribonucleoprotein U	4	8	mRNA processing	GO:0006397

O75643	U5 small nuclear ribonucleoprotein 200 kDa helicase	0	6	mRNA processing via splicesome	GO:0000398
P52272	Heterogeneous nuclear ribonucleoprotein M	2	5	mRNA processing	GO:0006397
Q9NZI8	[&¶]Insulin-like growth factor 2 mRNA-binding protein 1	0	4	mRNA processing	GO:0006397
Q6P2Q9	Pre-mRNA-processing-splicing factor 8	5	3	mRNA processing	GO:0006397
P52597	Heterogeneous nuclear ribonucleoprotein F	1	3	mRNA processing	GO:0006397
O00425	[&¶]Insulin-like growth factor 2 mRNA-binding protein 3	0	2	mRNA processing	GO:0006397
P61978	Heterogeneous nuclear ribonucleoprotein K	0	2	mRNA processing	GO:0006397
P84103	[§]Serine/arginine-rich splicing factor 3	1	3	mRNA processing via splicesome	GO:0000398
Q32P51	Heterogeneous nuclear ribonucleoprotein A1-like 2	0	3	mRNA processing	GO:0006397
O75533	Splicing factor 3B subunit 1	0	3	mRNA processing	GO:0006397
Q13310	[¶]Polyadenylate-binding protein 4	0	4	RNA processing	GO:0006396
P19338	[&]Nucleolin	4	14	developmental process<br>ribosome biogenesis	GO:0032502<br>GO:0042254
	**Helicase**				
P33993	DNA replication licensing factor MCM7	0	2	helicase activity	GO:0004386
Q9H2U1	Probable ATP-dependent RNA helicase DHX36	0	2	helicase activity	GO:0004386
Q08211	[&]ATP-dependent RNA helicase A DHX9	9	10	helicase activity	GO:0004386
Q7L2E3	Putative ATP-dependent RNA helicase DHX30	3	5	helicase activity	GO:0004386

Q9NR30	Nucleolar RNA helicase 2	1	4	helicase activity	GO:0004386
P12956	[¶]X-ray repair cross-complementing protein 6	3	3	helicase activity	GO:0004386
O00571	ATP-dependent RNA helicase DDX3X	2	3	helicase activity	GO:0004386
P17844	[¶]Probable ATP-dependent RNA helicase DDX5	1	3	helicase activity	GO:0004386
	**Translation**				
P13639	Elongation factor 2	0	2	translation factor activity	GO:0008135
	**Metabolic processes**				
P49327	Fatty acid synthase	2	10	metabolic process	GO:0008152
P11586	C-1-tetrahydrofolate synthase, cytoplasmic	0	4	metabolic process	GO:0008152
P36542	ATP synthase subunit gamma, mitochondrial	1	3	metabolic process	GO:0008152
P07814	[§]Bifunctional glutamate/proline--tRNA ligase	0	3	metabolic process	GO:0008152
P05141	ADP/ATP translocase 2	0	3	metabolic process	GO:0008152
Q15029	116 kDa U5 small nuclear ribonucleoprotein component	2	2	metabolic process	GO:0008152
P31943	[&]Heterogeneous nuclear ribonucleoprotein H	1	2	metabolic process	GO:0008152
O15067	Phosphoribosylformylglycinamidine synthase	0	2	metabolic process	GO:0008152
P00338	[¶]L-lactate dehydrogenase A chain	4	1	metabolic process	GO:0008152
P06733	Alpha-enolase	0	6	metabolic process	GO:0008152
	**Endocytosis**				
Q00610	[¶]Clathrin heavy chain 1	2	3	vesicle mediated transport	GO:0006897
P53621	Coatomer subunit alpha	4	1	vesicle mediated transport	GO:0006897

O95782	AP-2 complex subunit alpha-1	2	0	vesicle mediated transport	GO:0006897
	**Molecule transport**				
P21796	Voltage-dependent anion-selective channel protein 1	0	3	voltage-gated ion channel	PC00241
P45880	[¶]Voltage-dependent anion-selective channel protein 2	0	2	transporter activity	GO:0005215
Q00325	Phosphate carrier protein, mitochondrial	2	2	transporter	PC00227
P12236	ADP/ATP translocase 3	2	0	transporter activity	GO:0005215
	**Protein transport**				
O14617	AP-3 complex subunit delta-1	1	2	intracellular protein transport	GO:0006886
P55060	Exportin-2	0	2	transporter activity	GO:0005215
	**Additonal enzymatic function**				
P13010	X-ray repair cross-complementing protein 5	2	6	double-strand break repair via nonhomologous end joining	GO:0006303
P09874	Poly [ADP-ribose] polymerase 1	0	5	ligase activity	GO:0003909
P14618	[¶]Pyruvate kinase PKM	6	4	glycolytic process	GO:0006096
P41250	Glycine--tRNA ligase	0	4	aminoacyl-tRNA synthetase	PC00047
P17987	[§]T-complex protein 1 subunit alpha	0	3	chaperone	PC00072
P41252	[§]Isoleucine--tRNA ligase, cytoplasmic	0	3	aminoacyl-tRNA ligase activity	GO:0004812
P11940	[&¶]Polyadenylate-binding protein 1	4	2	developmental process	GO:0032502
P40227	[§]T-complex protein 1 subunit zeta	0	2	chaperone	PC00072
P56192	[§]Methionine--tRNA ligase, cytoplasmic	0	2	aminoacyl-tRNA synthetase	PC00047
P34897	Serine hydroxymethyltransferase, mitochondrial	0	2	transferase activity	GO:0016749
P30153	[¶]Serine/threonine-protein phosphatase 2A 65 kDa regulatory subunit A alpha isoform	0	2	phosphoprotein phosphatase activity	GO:0004721

P46940	Ras GTPase-activating-like protein IQGAP1	12	0	small GTPase regulator activity	GO:0005083
P62826	[§]GTP-binding nuclear protein Ran	2	0	hydrolase activity	GO:0016787
P04843	Dolichyl-diphosphooligosaccharide--protein glycosyltransferase subunit 1	2	0	transferase activity	GO:0016740
P17655	Calpain-2 catalytic subunit	2	0	hydrolase activity	GO:0016787
P04406	Glyceraldehyde-3-phosphate dehydrogenase	2	0	oxidoreductase activity	GO:0016491
P10809	60 kDa heat shock protein, mitochondrial	0	7	protein binding	GO:0005515
P78527	DNA-dependent protein kinase catalytic subunit	2	7	protein kinase activity	GO:0004672

Display options: Exclusive Unique Peptide Count, No filter selected for Req Mods

Scaffold Viewer settings summary: protein threshold 95%, min # peptides 2, peptide threshold 95%

Note: desmin, Interleukin enhancer binding factor 2 (ILF2), DDX17 and peroxiredoxin are not mentioned as only one peptide was identified.

*Identified as a putative DUX4-binding partner using yeast two-hybrid (see table 3)

[¶]Identified (or highly similar) as a putative DUX4 binding partner using HaloTag co-purification (see S3 Table)

[§]Isoform (or similar function) identified in other approaches (see other tables)

Validated interaction by co-immunoprecipitation in HEK293 cells

[&]Known to be associated in an IGF2BP1-dependent mRNP complex

Any peptide of the proteins identified were found in the different controls made in parallel (non-specific binding peptides purified following incubation of Glutathione Sepharose 4B with total protein lysate without addition of GST-DUX4), except for tubulin beta-chain (8 peptides found with HEK293 lysate but no peptide found with myoblast lysate); actin 2 (8 peptides found with myoblast lysate but no peptide found with HEK293 lysate); C1QBP, alpha-enolase and 60 kDa heat shock protein (1 peptide found with HEK293 lysate alone but no peptide found with myoblast lysate); and myosin 9, vimentin and Ribosomal Protein P2 (1 peptide found with myoblast lysate but no peptide found with HEK293 lysate).
