# Supplementary material for: Homologous Transcription Factors DUX4 and DUX4c Associate with Cytoplasmic Proteins during Muscle Differentiation
Source: PLoS One. 2016 Jan 27;11(1):e0146893. doi: 10.1371/journal.pone.0146893 (PMC4729438; doi:10.1371/journal.pone.0146893)
Supplement: S1 Text — (PDF) [file pone.0146893.s018.pdf]

Supporting Informations

Many putative DUX partners are involved in actin remodeling, which is a crucial step at the beginning of the muscle differentiation process. Moreover, most partners are located at the Z-discs in mature skeletal muscle fibers; Z-discs are dynamic structures that undergo continuous protein exchange with cytoplasmic pools [1]. The major DUX-binding partner that we identified is desmin, a component of type III IF.

Several putative cytoplasmic DUX partners that need confirmations are known to be Z-line proteins or playing role in actin remodeling, such as α-actinins and the two fodrin subunits, among others (see Tables 2-S3 and description hereafter). Alpha-actinins together with synemin are critical in cytoskeletal organization and serve to anchor the actin-containing thin filaments and desmin-containing IFs to both myofibrillar Z-lines and costameres [2]. Fodrin plays a pivotal role in maintaining the integrity of the cytoskeletal structure and interacts with ARP1/alpha-centractin [3,4]. Alpha-fodrin contains a binding site for calmodulin [5], which was also observed among the putative DUX4 partners and regulates the self-association of fodrin and its interaction with F-actin [6]. Cytosolic ARP proteins contribute to actin filament assembly and organization [7, 8]. Gelsolin is a potent actin filament-severing and -capping protein with a major role in cytoskeletal remodeling and in skeletal muscle development 9,10]. Synaptopodin interacts with actin and α-actinin [11]. Nucleophosmin binds to β-actin and vimentin, the desmin homologue expressed in myoblasts [12]. Arf4 function is critical in actin cytoskeletal assembly [13. FHL3 and its paralog LIMS2 link integrin to the actin cytoskeleton [14,15]. In differentiating myoblasts, FHL3 is actively excluded from the nucleus and regulates actin cytoskeletal dynamics [16]. RBBP4 has been reported to regulate cytoskeletal F-actin organization [17].

References :

1. Wang J, Shaner N, Mittal B, Zhou Q, Chen J, Sanger JM, Sanger JW (2005) Dynamics of Z-band based proteins in developing skeletal muscle cells. Cell Motil Cytoskeleton 61:34–48. doi: 10.1002/cm.20063

2. Bellin RM, Huiatt TW, Critchley DR, Robson RM (2001) Synemin may function to directly link muscle cell intermediate filaments to both myofibrillar Z-lines and costameres. J Biol Chem 276:32330–32337. doi: 10.1074/jbc.M104005200

3. Holleran EA, Tokito MK, Karki S, Holzbaur EL (1996) Centractin (ARP1) associates with spectrin revealing a potential mechanism to link dynactin to intracellular organelles. J Cell Biol 135:1815–1829.

4. Shashikala S, Kumar R, Thomas NE, Sivadasan D, James J, Sengupta S (2013) Fodrin in centrosomes: implication of a role of fodrin in the transport of gamma-tubulin complex in brain. PLoS ONE 8:e76613. doi: 10.1371/journal.pone.0076613

5. Simonovic M, Zhang Z, Cianci CD, Steitz TA, Morrow JS (2006) Structure of the calmodulin alphaII-spectrin complex provides insight into the regulation of cell plasticity. J Biol Chem 281:34333–34340. doi: 10.1074/jbc.M604613200

6. Harris AS, Morrow JS (1990) Calmodulin and calcium-dependent protease I coordinately regulate the interaction of fodrin with actin. Proc Natl Acad Sci USA 87:3009–3013.

7. Suetsugu S, Miki H, Yamaguchi H, Obinata T, Takenawa T (2001) Enhancement of branching efficiency by the actin filament-binding activity of N-WASP/WAVE2. J Cell Sci 114:4533–4542.

8. Weaver AM, Karginov AV, Kinley AW, Weed SA, Li Y, Parsons JT, Cooper JA (2001) Cortactin promotes and stabilizes Arp2/3-induced actin filament network formation. Curr Biol 11:370–374.

9. Dissmann E, Hinssen H (1994) Immunocytochemical localization of gelsolin in fibroblasts, myogenic cells, and isolated myofibrils. Eur J Cell Biol 63:336–344.

10. Scholz A, Hinssen H (1995) Biphasic pattern of gelsolin expression and variations in gelsolin-actin interactions during myogenesis. Exp Cell Res 219:384–391. doi: 10.1006/excr.1995.1243

11. Kremerskothen J, Plaas C, Kindler S, Frotscher M, Barnekow A (2005) Synaptopodin, a molecule involved in the formation of the dendritic spine apparatus, is a dual actin/alpha-actinin binding protein. J Neurochem 92:597–606. doi: 10.1111/j.1471-4159.2004.02888.x

12. Cicchillitti L, Della Corte A, Di Michele M, Donati MB, Rotilio D, Scambia G (2010) Characterisation of a multimeric protein complex associated with ERp57 within the nucleus in paclitaxel-sensitive and -resistant epithelial ovarian cancer cells: the involvement of specific conformational states of beta-actin. Int J Oncol 37:445–454.

13. Mazelova J, Astuto-Gribble L, Inoue H, Tam BM, Schonteich E, Prekeris R, Moritz OL, Randazzo PA, Deretic D (2009) Ciliary targeting motif VxPx directs assembly of a trafficking module through Arf4. EMBO J 28:183–192. doi: 10.1038/emboj.2008.267

14. Samson T, Smyth N, Janetzky S, Wendler O, Müller JM, Schüle R, von der Mark H, von der Mark K, Wixler V (2004) The LIM-only proteins FHL2 and FHL3 interact with alpha- and beta-subunits of the muscle alpha7beta1 integrin receptor. J Biol Chem 279:28641–28652. doi: 10.1074/jbc.M312894200

15. Legate KR, Montañez E, Kudlacek O, Fässler R (2006) ILK, PINCH and parvin: the tIPP of integrin signalling. Nat Rev Mol Cell Biol 7:20–31. doi: 10.1038/nrm1789

16. Coghill ID, Brown S, Cottle DL, McGrath MJ, Robinson PA, Nandurkar HH, Dyson JM, Mitchell CA (2003) FHL3 is an actin-binding protein that regulates alpha-actinin-mediated actin bundling: FHL3 localizes to actin stress fibers and enhances cell spreading and stress fiber disassembly. J Biol Chem 278:24139–24152. doi: 10.1074/jbc.M213259200

17. Scuto A, Zhang H, Zhao H, Rivera M, Yeatman TJ, Jove R, Torres-Roca JF (2007) RbAp48 regulates cytoskeletal organization and morphology by increasing K-Ras activity and signaling through mitogen-activated protein kinase. Cancer Res 67:10317–10324. doi: 10.1158/0008-5472.CAN-06-3313
